# Supplementary figures and images for: Paradoxical dominant negative activity of an immunodeficiency-associated activating PIK3R1 variant (part 1 of 2)
Source: eLife. 2025 Jan 21;13:RP94420. doi: 10.7554/eLife.94420 (PMC11750134; doi:10.7554/eLife.94420)

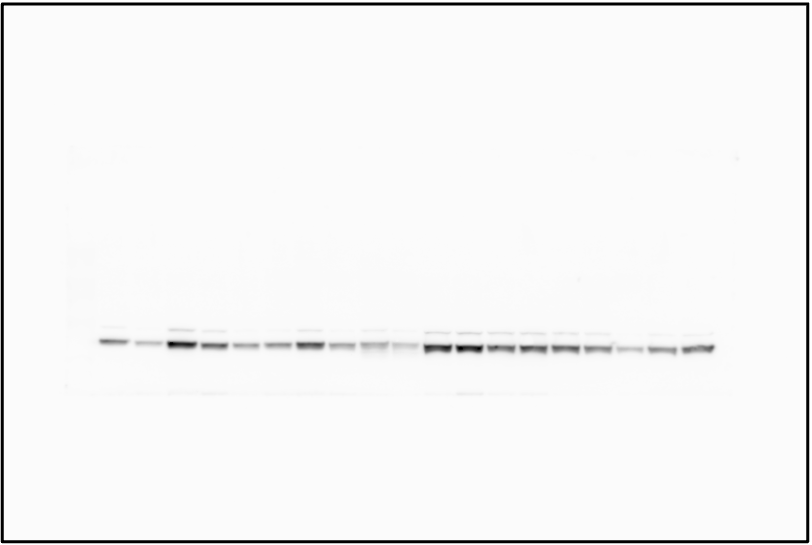

Supplement: Figure 1—source data 1. [file elife-94420-fig1-data1.zip › Figure_1A_and_Figure 1-figure_supplement_2B,C_source_data_1/Fig1A_pAKT_T308_replicate2_source image.png]

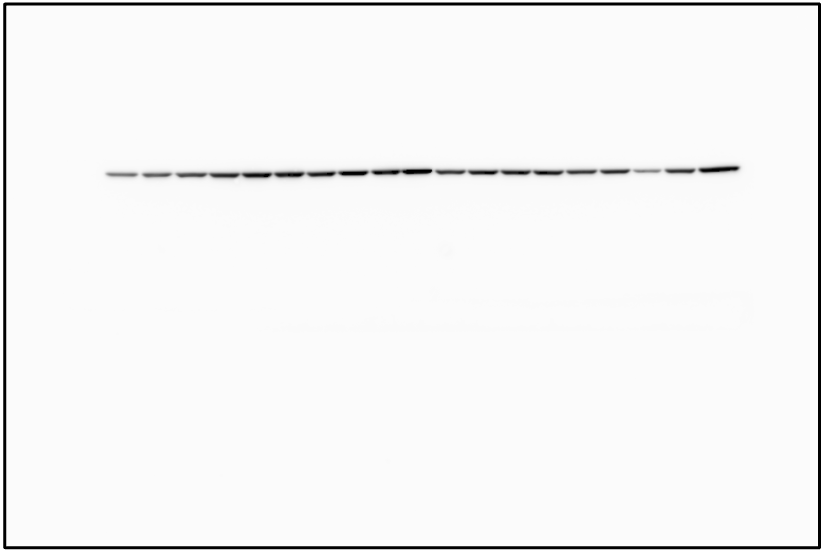

Supplement: Figure 1—source data 1. [file elife-94420-fig1-data1.zip › Figure_1A_and_Figure 1-figure_supplement_2B,C_source_data_1/Fig1A_beta_actin_source_image.png]

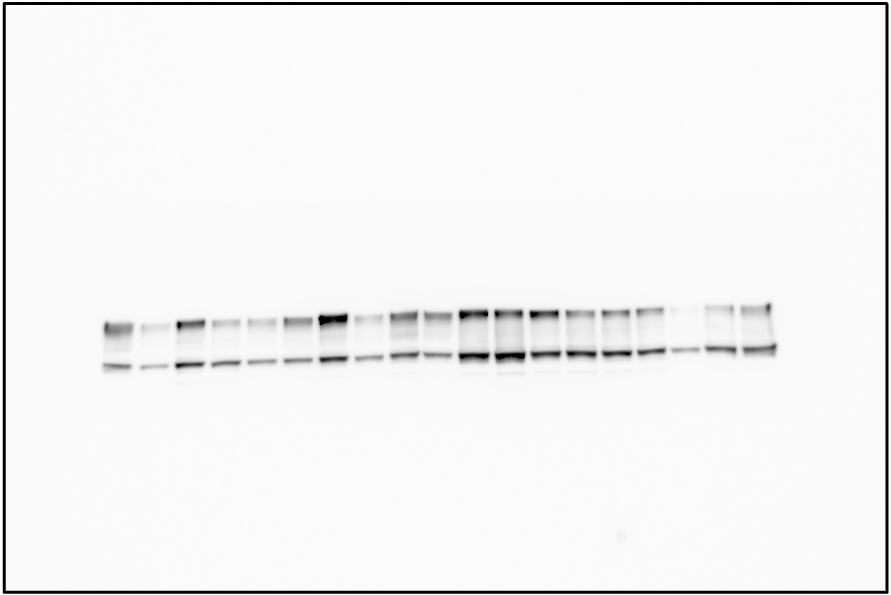

Supplement: Figure 1—source data 1. [file elife-94420-fig1-data1.zip › Figure_1A_and_Figure 1-figure_supplement_2B,C_source_data_1/Fig1A_p110alpha_replicate2_source image.png]

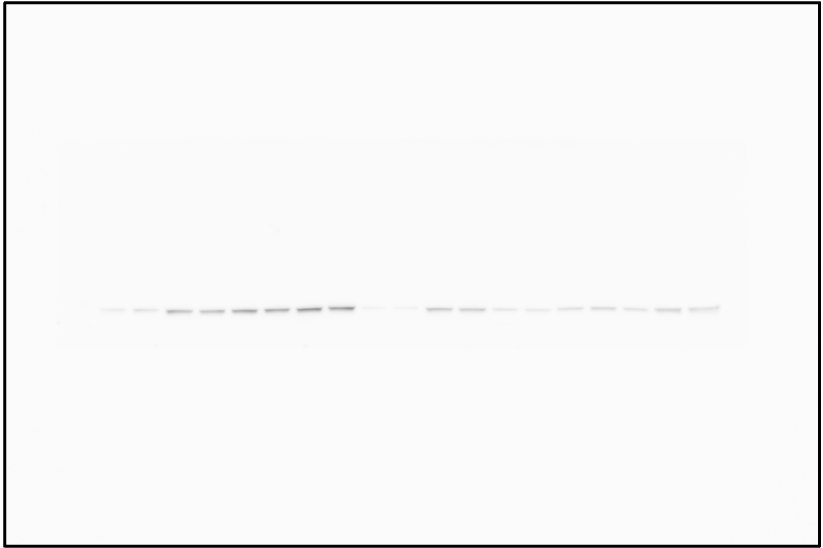

Supplement: Figure 1—source data 1. [file elife-94420-fig1-data1.zip › Figure_1A_and_Figure 1-figure_supplement_2B,C_source_data_1/Fig1A_p110deltaand_Fig1_figure_supplement_2_source_image.png]

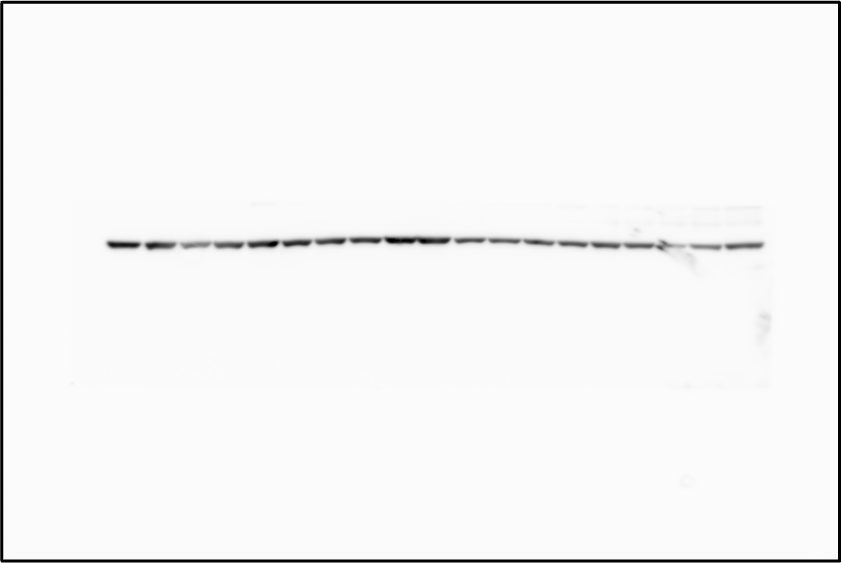

Supplement: Figure 1—source data 1. [file elife-94420-fig1-data1.zip › Figure_1A_and_Figure 1-figure_supplement_2B,C_source_data_1/Fig1A_beta_actin_replicate1_source image.png]

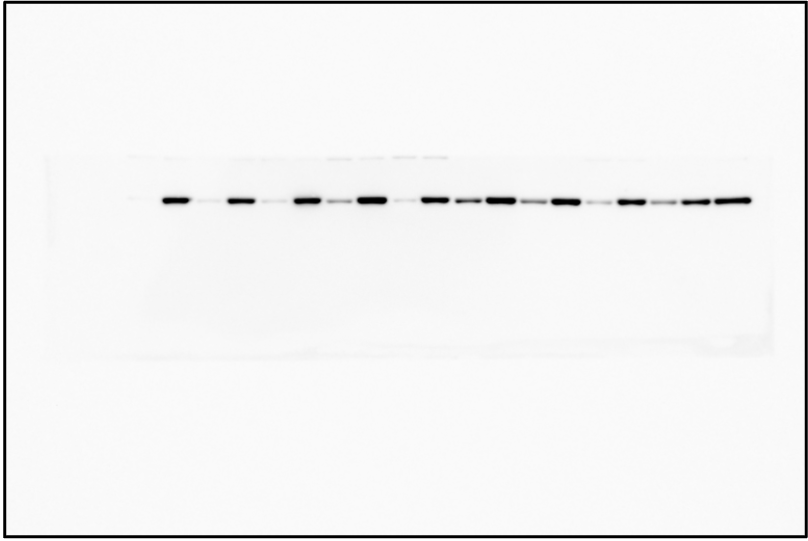

Supplement: Figure 1—source data 1. [file elife-94420-fig1-data1.zip › Figure_1A_and_Figure 1-figure_supplement_2B,C_source_data_1/Fig1A_pAKT_S473_replicate1_source image.png]

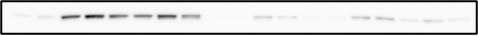

Supplement: Figure 1—source data 1. [file elife-94420-fig1-data1.zip › Figure_1A_and_Figure 1-figure_supplement_2B,C_source_data_1/Fig1A_and_Fig1-figure_supplement_2_p110delta_replicate1_source image.jpg]

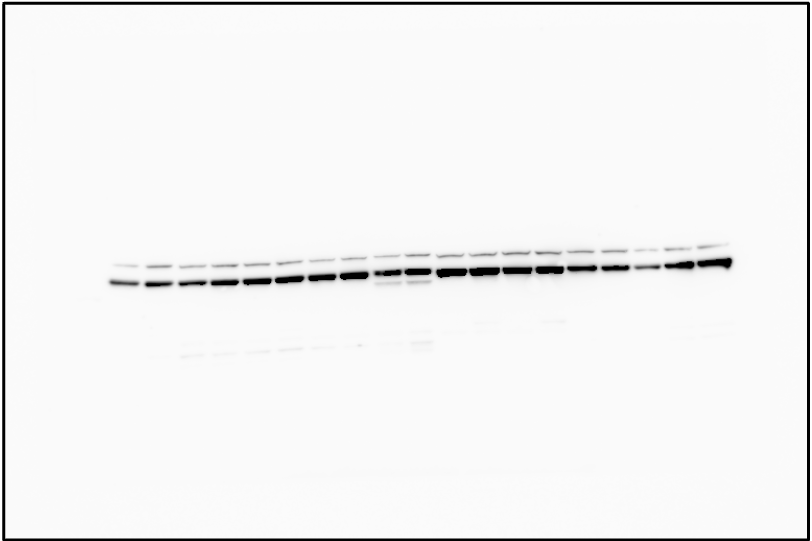

Supplement: Figure 1—source data 1. [file elife-94420-fig1-data1.zip › Figure_1A_and_Figure 1-figure_supplement_2B,C_source_data_1/Fig1A_and_Fig1_figure_supplement_2_p85alpha_source_image.png]

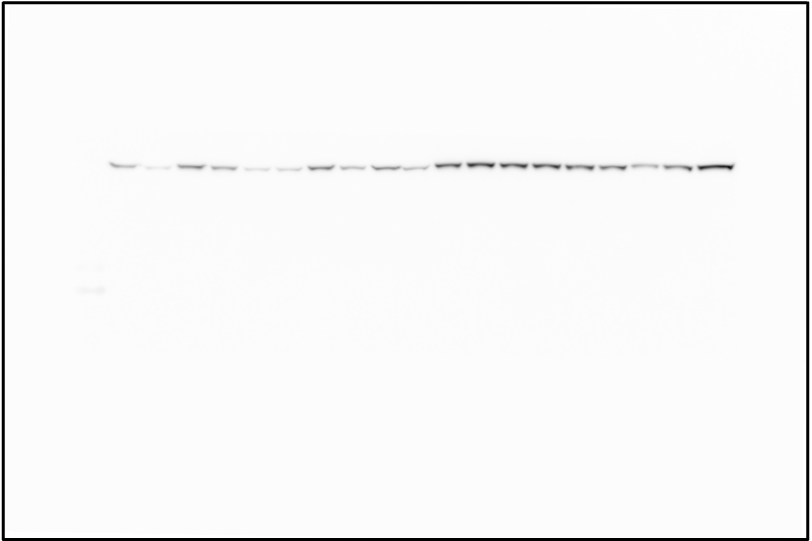

Supplement: Figure 1—source data 1. [file elife-94420-fig1-data1.zip › Figure_1A_and_Figure 1-figure_supplement_2B,C_source_data_1/Fig1A_AKT_replicate2_source image.png]

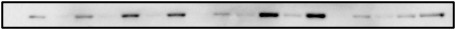

Supplement: Figure 1—source data 1. [file elife-94420-fig1-data1.zip › Figure_1A_and_Figure 1-figure_supplement_2B,C_source_data_1/Fig1A_pAKT_T308_source_image.jpg]

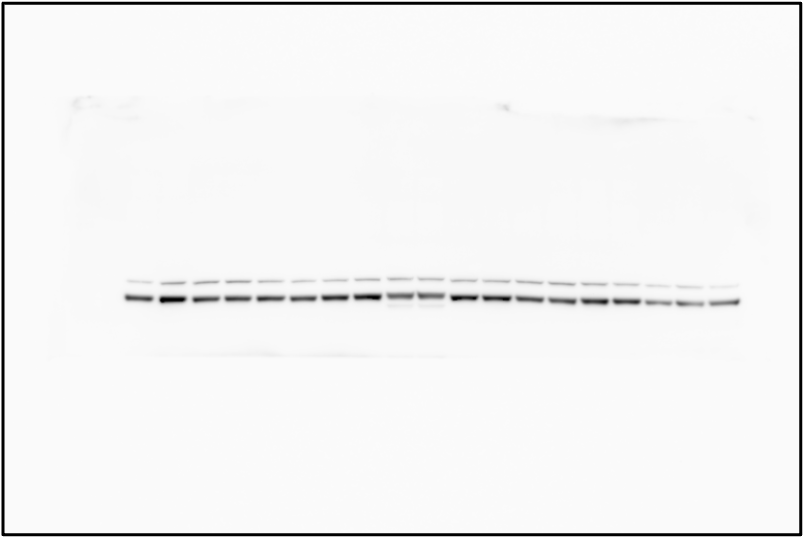

Supplement: Figure 1—source data 1. [file elife-94420-fig1-data1.zip › Figure_1A_and_Figure 1-figure_supplement_2B,C_source_data_1/Fig1A_p95_alpha_replicate1_source image.png]

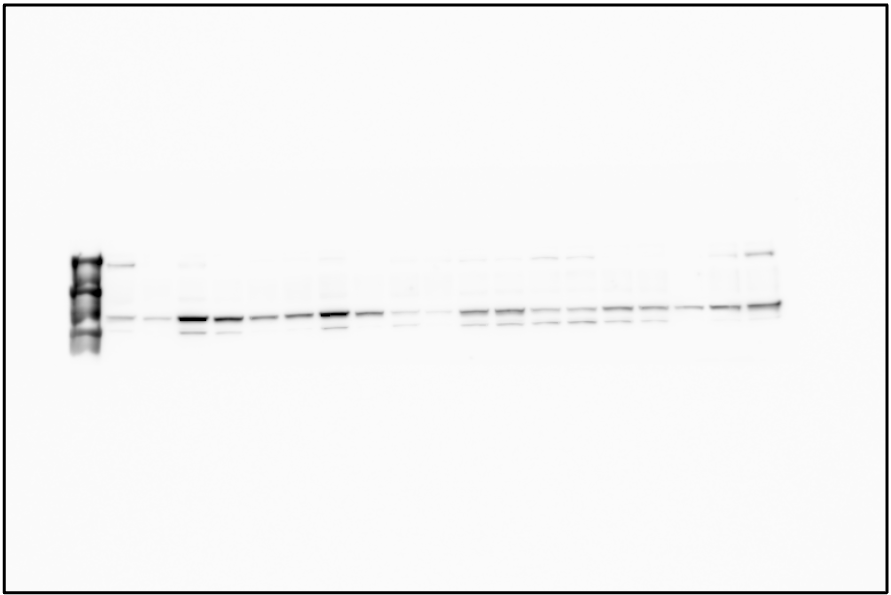

Supplement: Figure 1—source data 1. [file elife-94420-fig1-data1.zip › Figure_1A_and_Figure 1-figure_supplement_2B,C_source_data_1/Fig1A_andFig1-figure_supplement_2_p110delta_replicate2_source image.png]

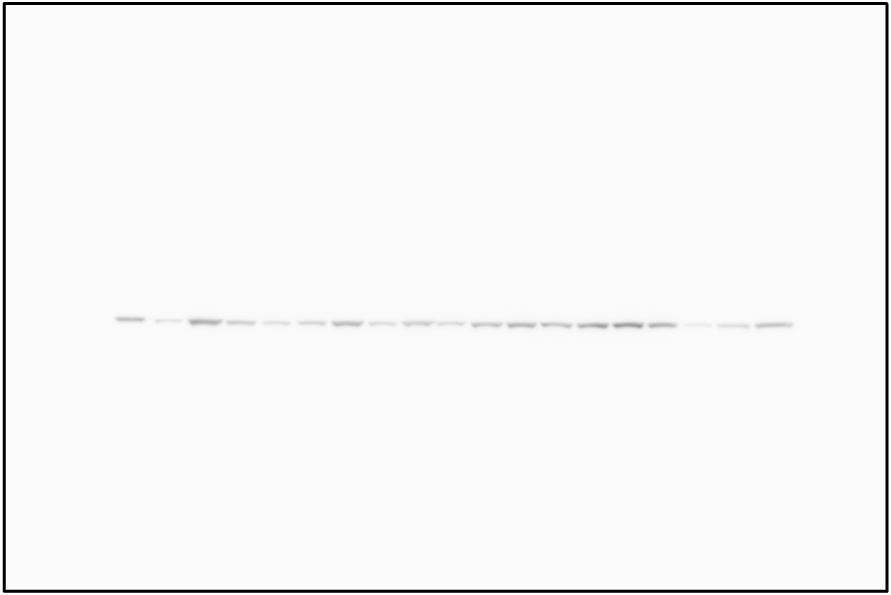

Supplement: Figure 1—source data 1. [file elife-94420-fig1-data1.zip › Figure_1A_and_Figure 1-figure_supplement_2B,C_source_data_1/Fig1A_beta_actin_replicate2_source image.png]

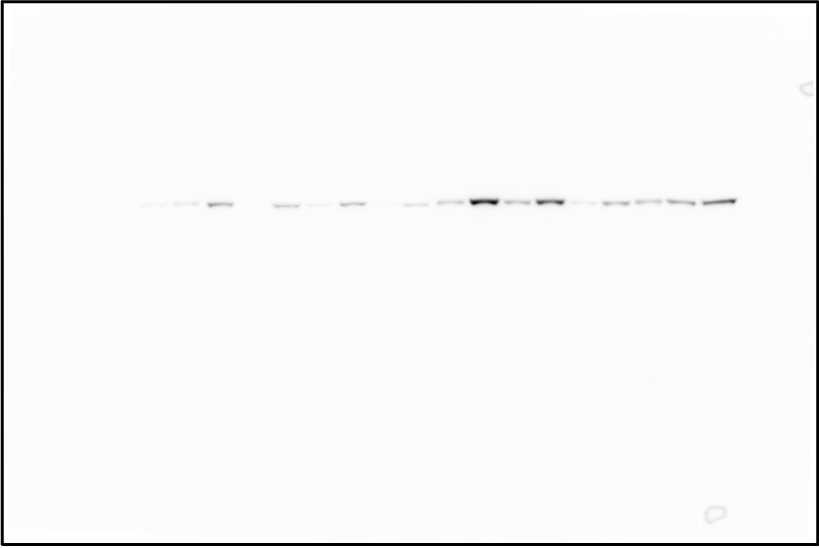

Supplement: Figure 1—source data 1. [file elife-94420-fig1-data1.zip › Figure_1A_and_Figure 1-figure_supplement_2B,C_source_data_1/Fig1A_pAKT_S473_replicate2_source image.png]

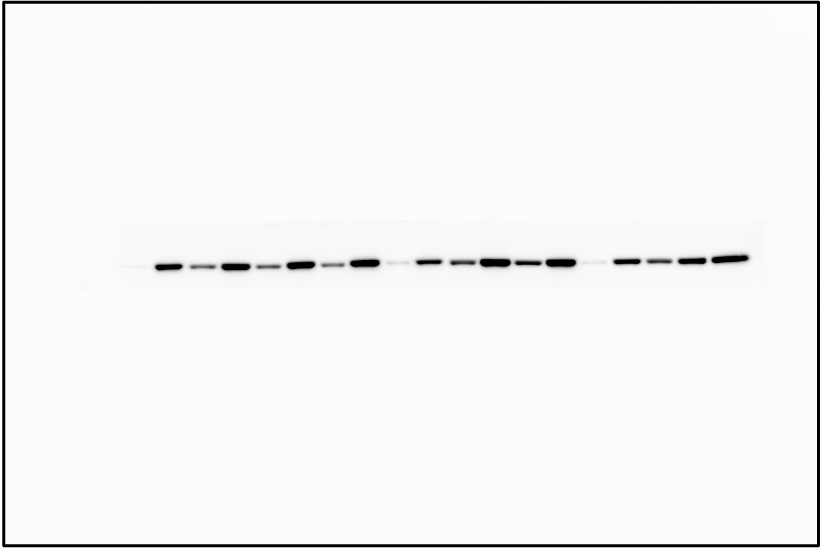

Supplement: Figure 1—source data 1. [file elife-94420-fig1-data1.zip › Figure_1A_and_Figure 1-figure_supplement_2B,C_source_data_1/Fig1A_pAKT_S473_source_image.png]

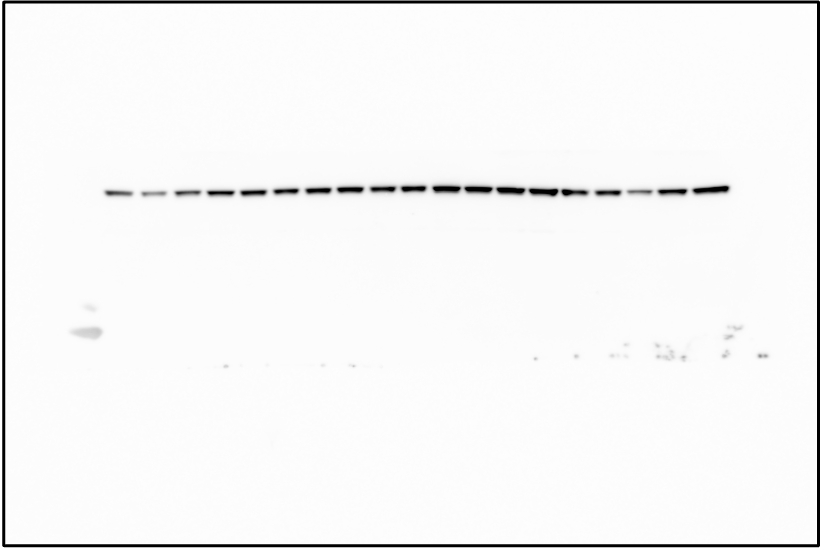

Supplement: Figure 1—source data 1. [file elife-94420-fig1-data1.zip › Figure_1A_and_Figure 1-figure_supplement_2B,C_source_data_1/Fig1A_AKT_source_image.png]

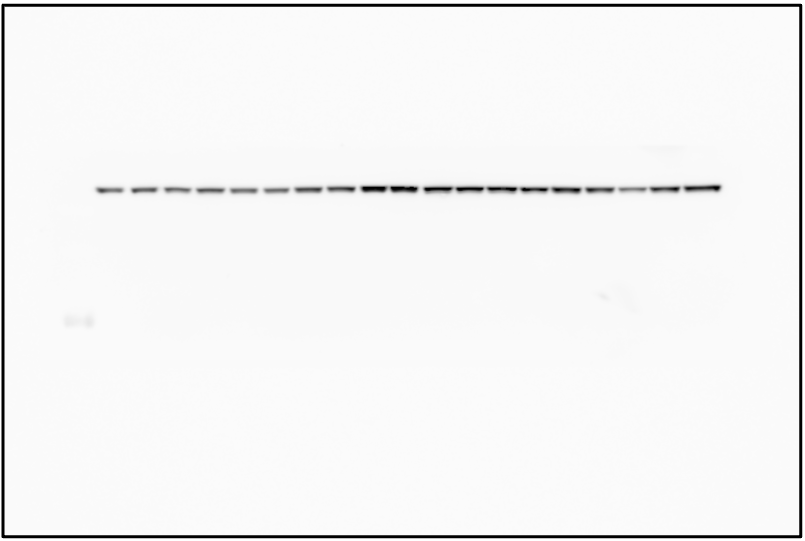

Supplement: Figure 1—source data 1. [file elife-94420-fig1-data1.zip › Figure_1A_and_Figure 1-figure_supplement_2B,C_source_data_1/Fig1A_AKT_replicate1_source image.png]

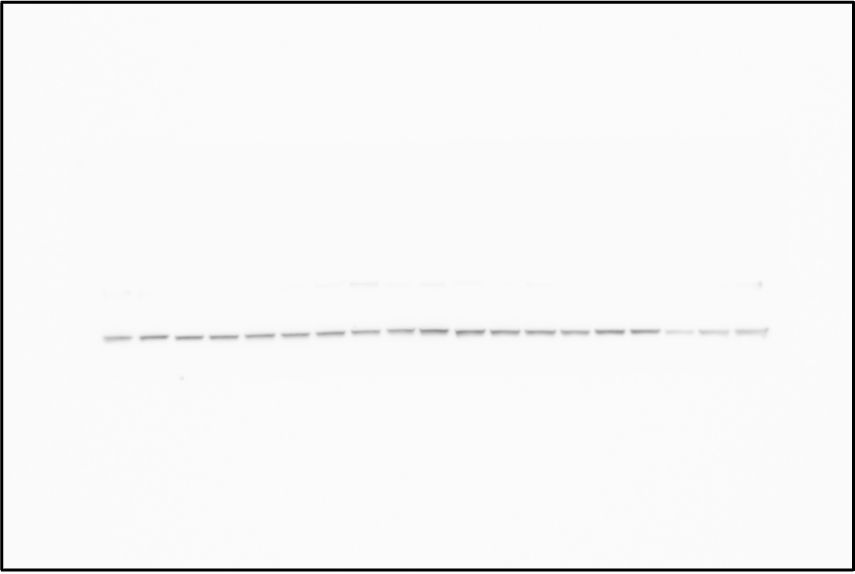

Supplement: Figure 1—source data 1. [file elife-94420-fig1-data1.zip › Figure_1A_and_Figure 1-figure_supplement_2B,C_source_data_1/Fig1A_p110alpha_replicate1_source image.png]

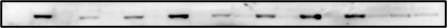

Supplement: Figure 1—source data 1. [file elife-94420-fig1-data1.zip › Figure_1A_and_Figure 1-figure_supplement_2B,C_source_data_1/Fig1A_pAKT_T308_replicate1_source image.jpg]

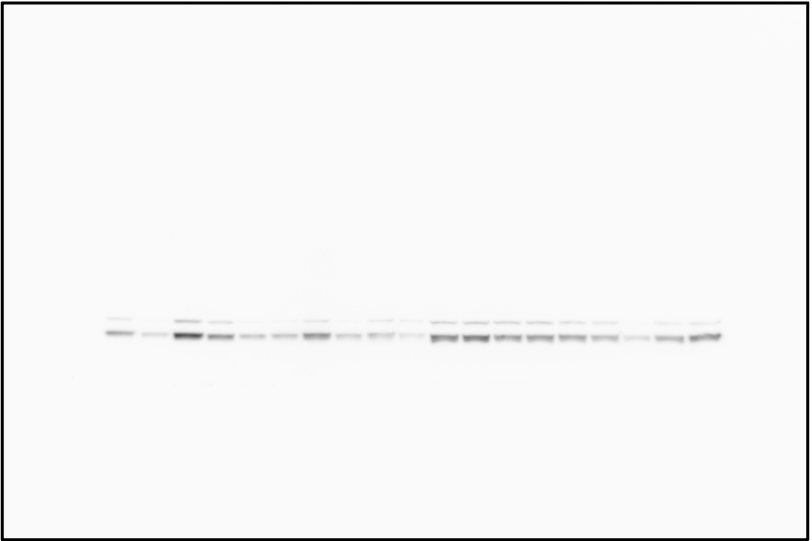

Supplement: Figure 1—source data 1. [file elife-94420-fig1-data1.zip › Figure_1A_and_Figure 1-figure_supplement_2B,C_source_data_1/Fig1A_p85alpha_replicate2_source image.png]

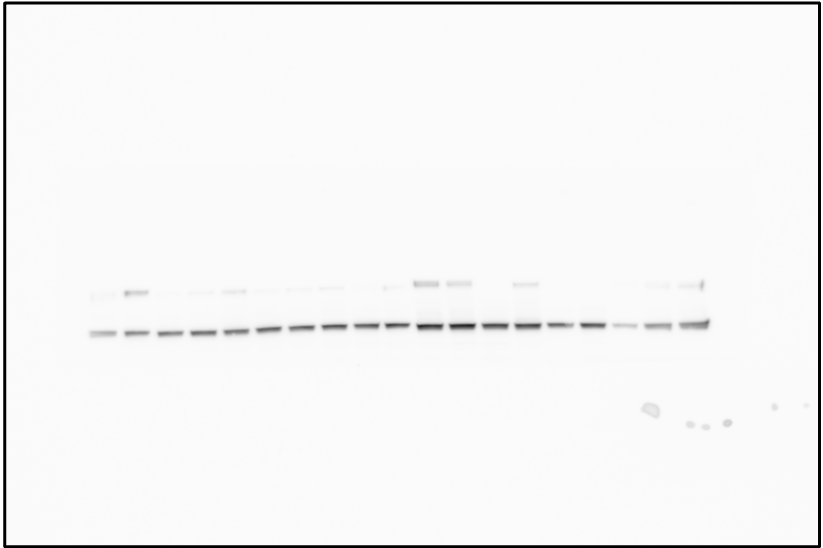

Supplement: Figure 1—source data 1. [file elife-94420-fig1-data1.zip › Figure_1A_and_Figure 1-figure_supplement_2B,C_source_data_1/Fig1A_p110alpha_source_image.png]

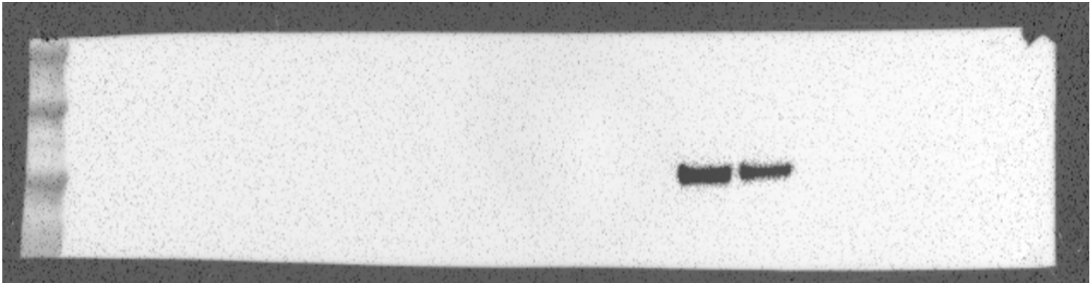

Supplement: Figure 2—source data 1. [file elife-94420-fig2-data1.zip › Figure_2_source_data_1/Fig2A_p110alpha_source_image.png]

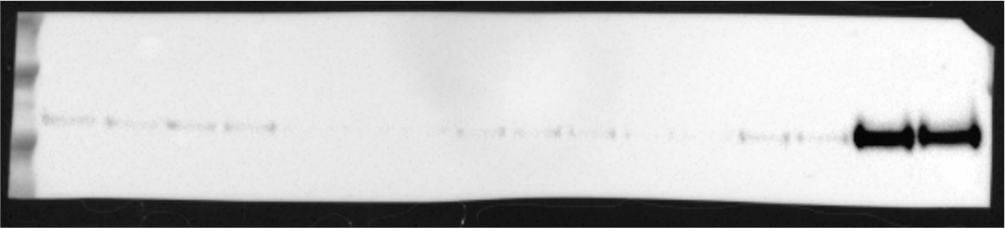

Supplement: Figure 2—source data 1. [file elife-94420-fig2-data1.zip › Figure_2_source_data_1/Fig2A_p110alpha_replicate_1_source_image.png]

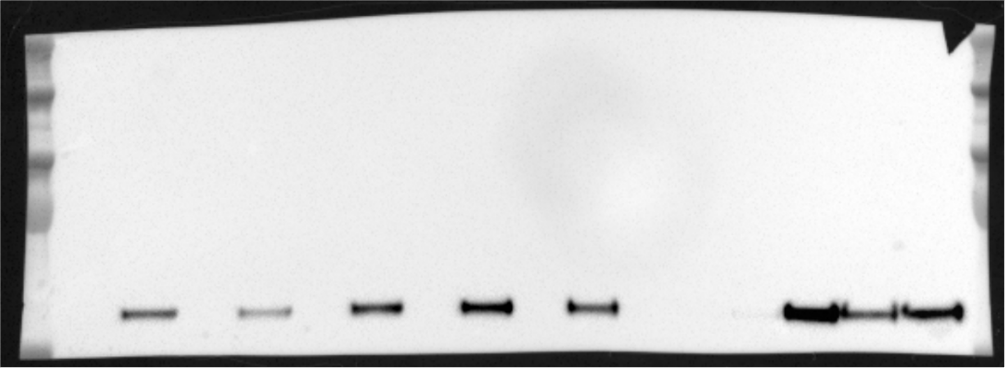

Supplement: Figure 2—source data 1. [file elife-94420-fig2-data1.zip › Figure_2_source_data_1/Fig2A_pAKT_S473_replicate_1_source_image.png]

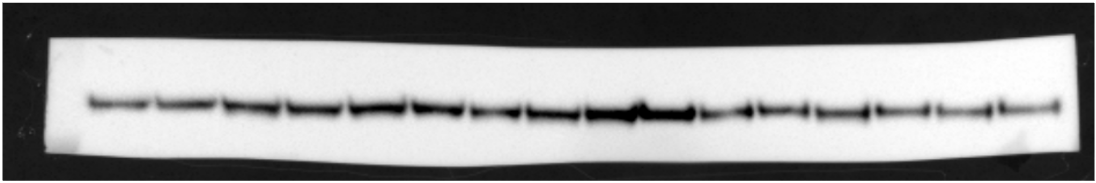

Supplement: Figure 2—source data 1. [file elife-94420-fig2-data1.zip › Figure_2_source_data_1/Fig2A_AKT_source_image.png]

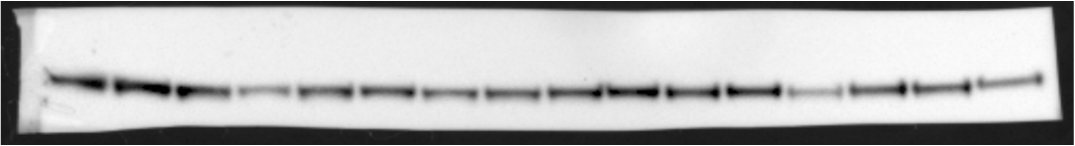

Supplement: Figure 2—source data 1. [file elife-94420-fig2-data1.zip › Figure_2_source_data_1/Fig2A_AKT_replicate_2_source_image.png]

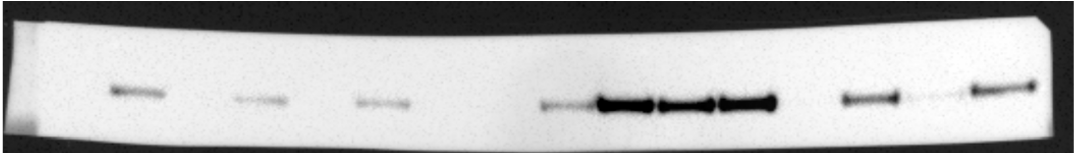

Supplement: Figure 2—source data 1. [file elife-94420-fig2-data1.zip › Figure_2_source_data_1/Fig2A_pAKT_T308_replicate_2_source_image.png]

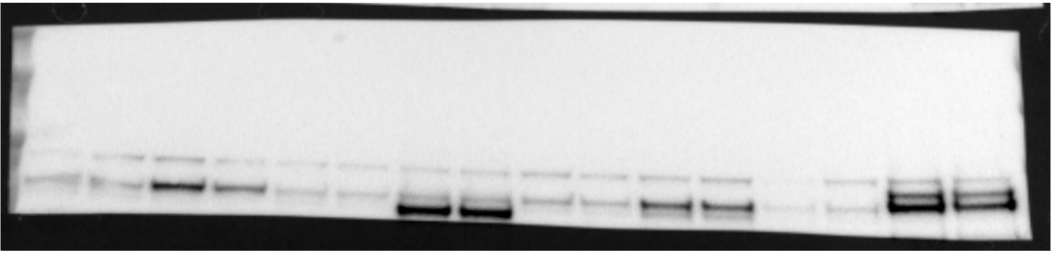

Supplement: Figure 2—source data 1. [file elife-94420-fig2-data1.zip › Figure_2_source_data_1/Fig2A_p85alpha_replicate_2_source_image.png]

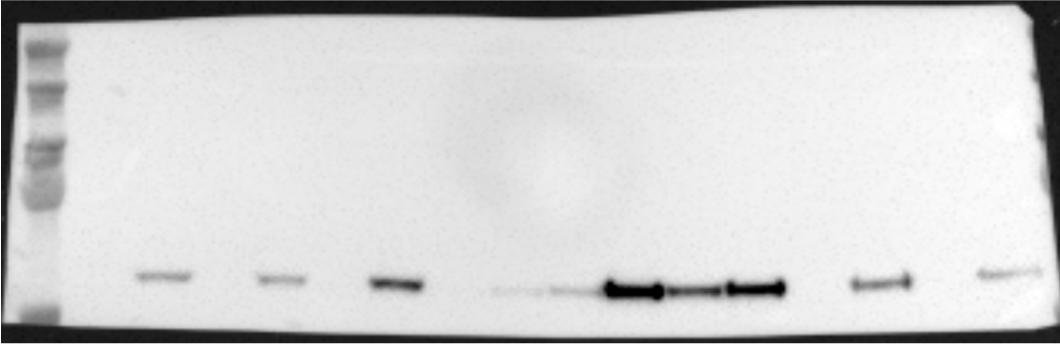

Supplement: Figure 2—source data 1. [file elife-94420-fig2-data1.zip › Figure_2_source_data_1/Fig2A_pAKT_S473_source_image.png]

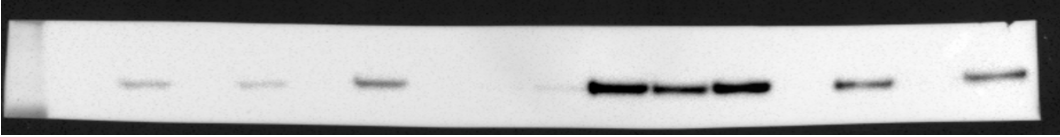

Supplement: Figure 2—source data 1. [file elife-94420-fig2-data1.zip › Figure_2_source_data_1/Fig2A_pAKT_T308_source_image.png]

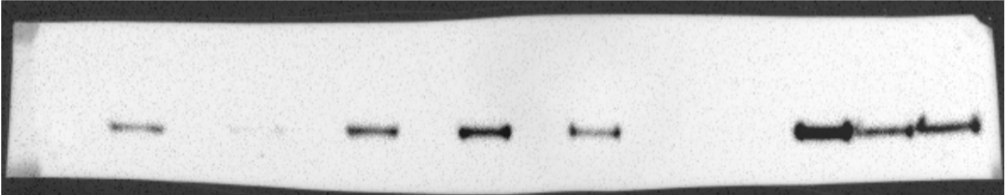

Supplement: Figure 2—source data 1. [file elife-94420-fig2-data1.zip › Figure_2_source_data_1/Fig2A_pAKT_T308_replicate_1_source_image.png]

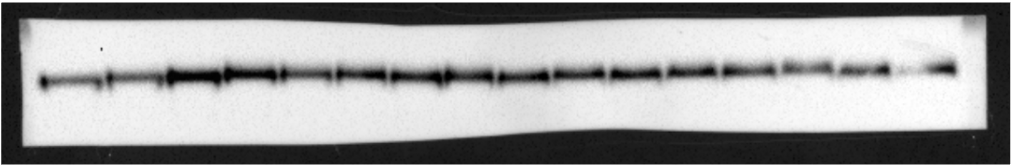

Supplement: Figure 2—source data 1. [file elife-94420-fig2-data1.zip › Figure_2_source_data_1/Fig2A_AKT_replicate_1_source_image.png]

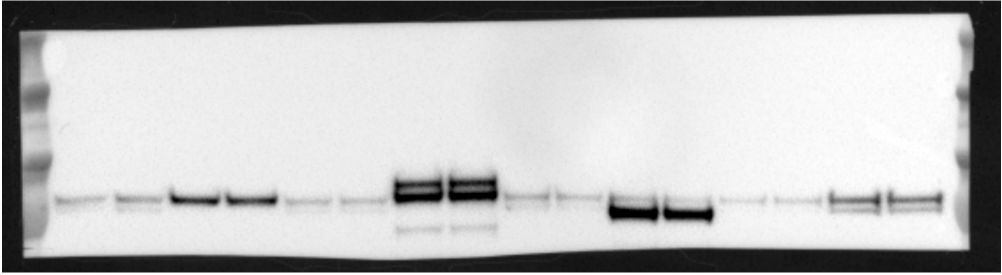

Supplement: Figure 2—source data 1. [file elife-94420-fig2-data1.zip › Figure_2_source_data_1/Fig2A_p85alpha_replicate_1_source_image.png]

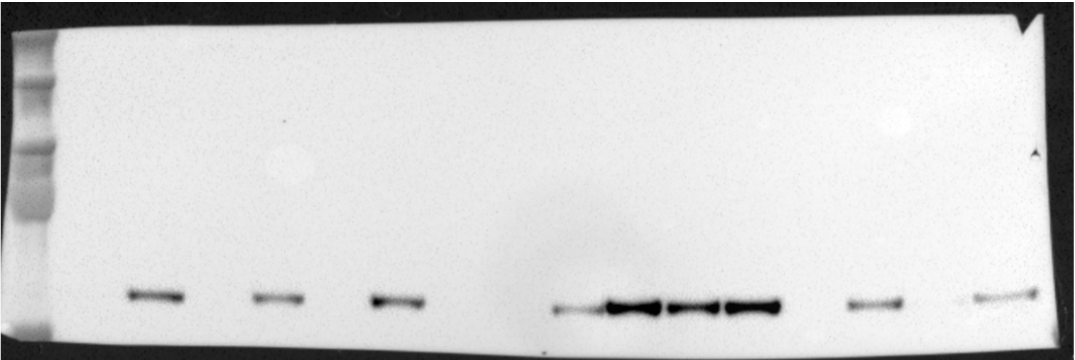

Supplement: Figure 2—source data 1. [file elife-94420-fig2-data1.zip › Figure_2_source_data_1/Fig2A_pAKT_S473_replicate_2_source_image.png]

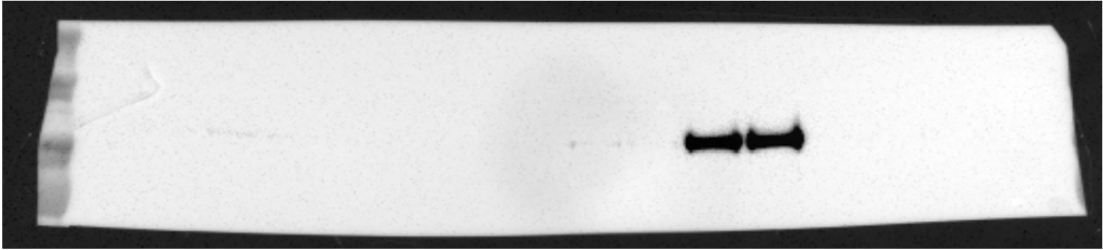

Supplement: Figure 2—source data 1. [file elife-94420-fig2-data1.zip › Figure_2_source_data_1/Fig2A_p110alpha_replicate_2_source_image.png]

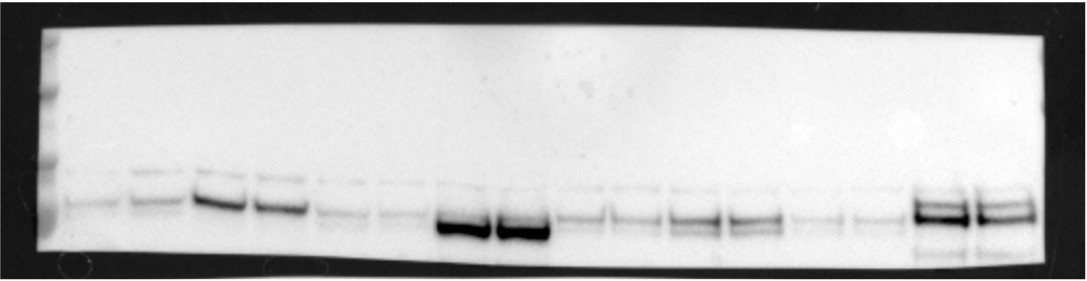

Supplement: Figure 2—source data 1. [file elife-94420-fig2-data1.zip › Figure_2_source_data_1/Fig2A_p85alpha_source_image.png]

Figure 2D – Images shown

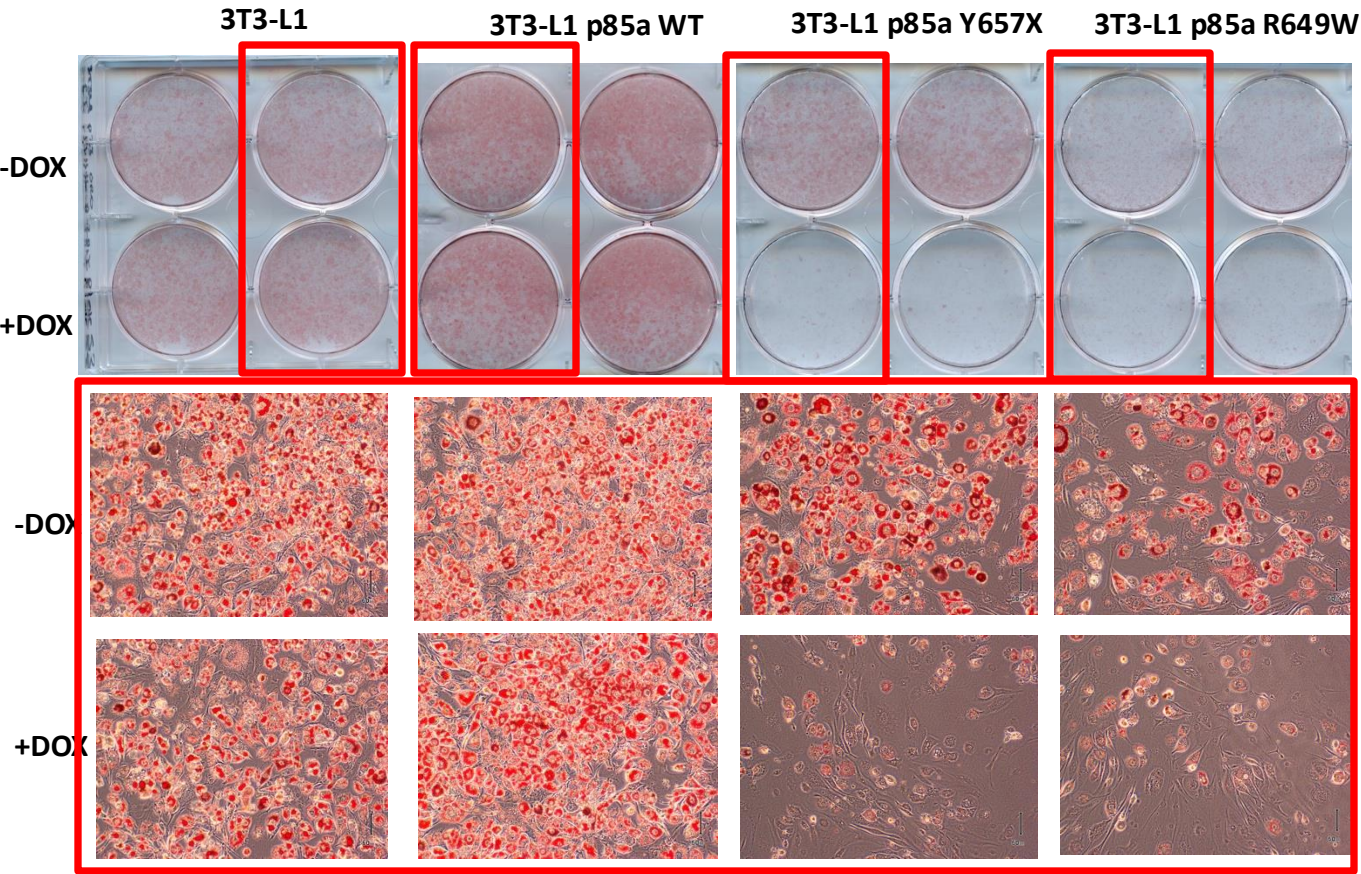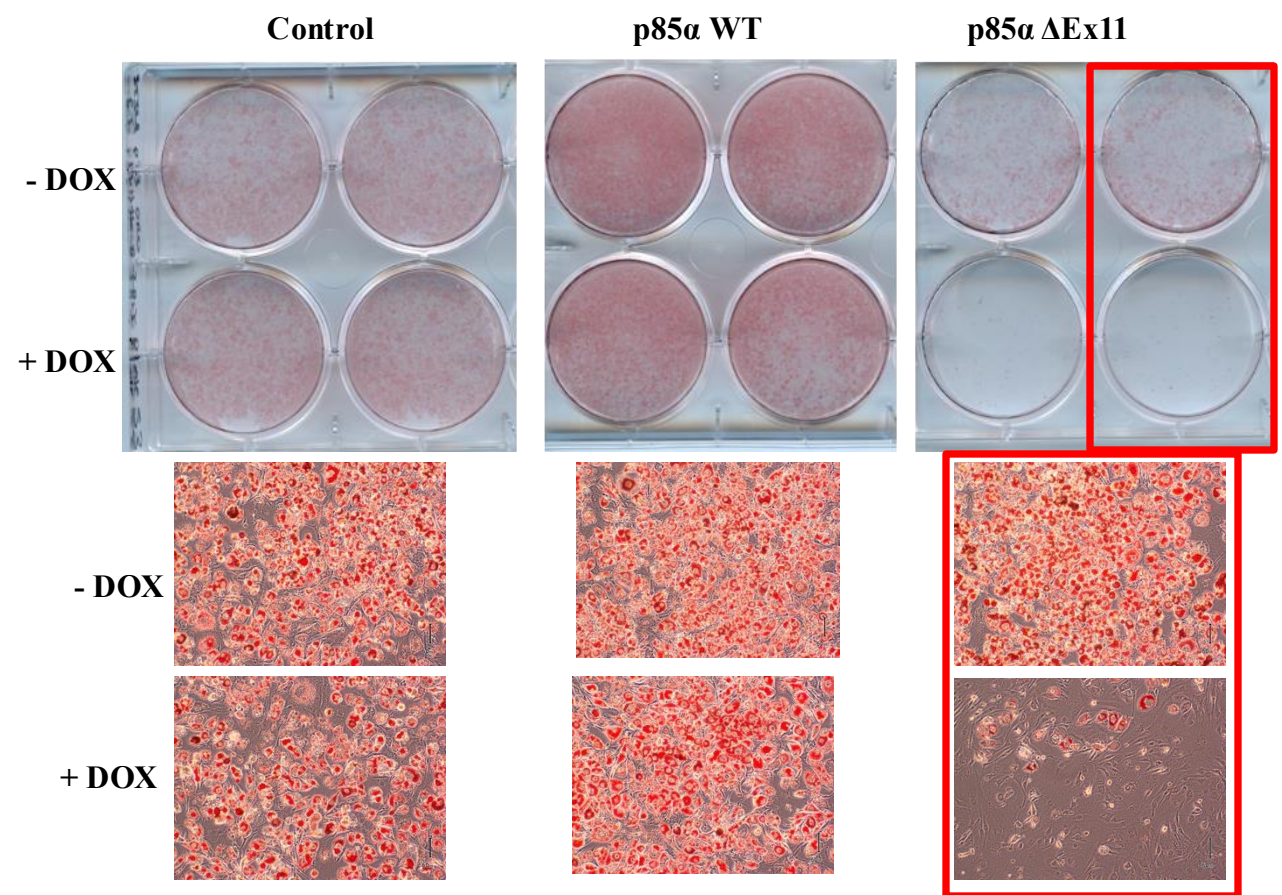

Figure 2D – Replicate 1

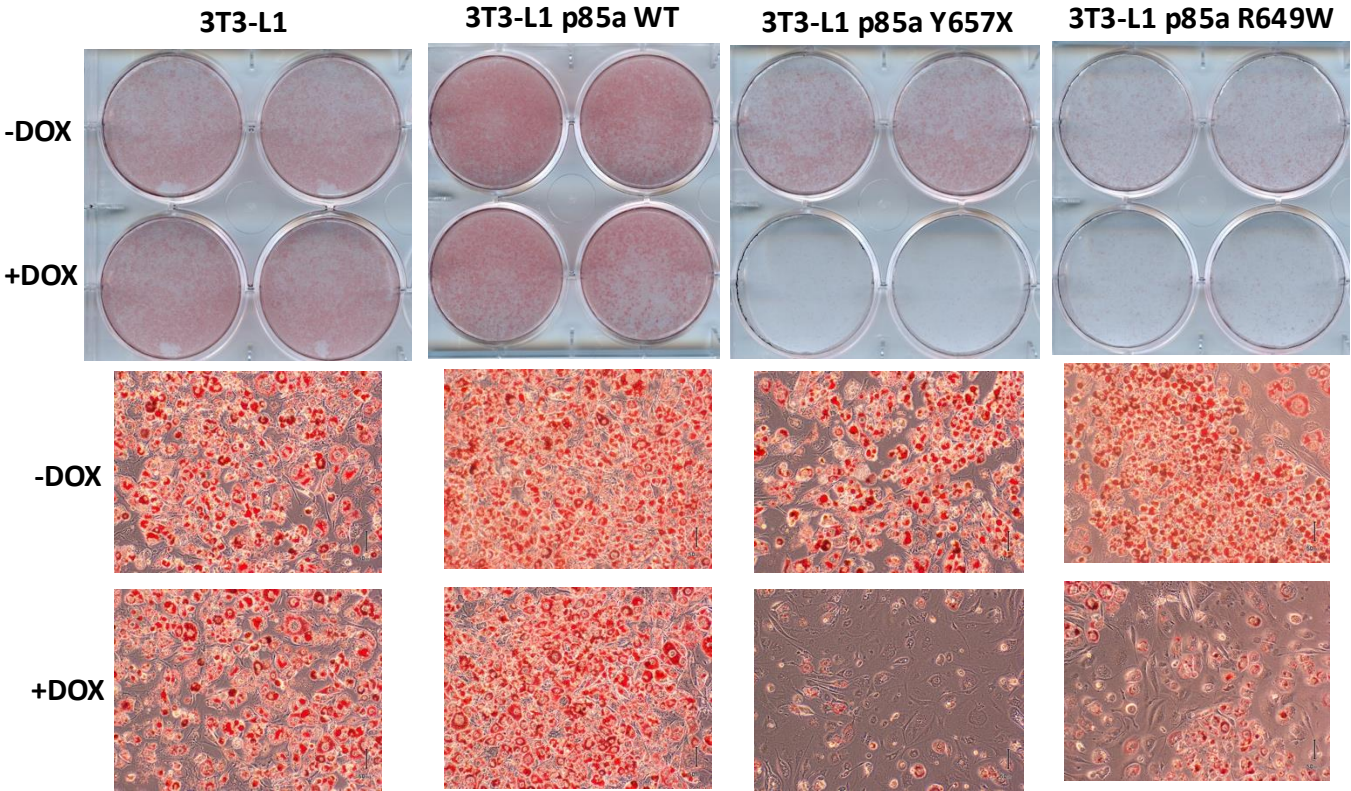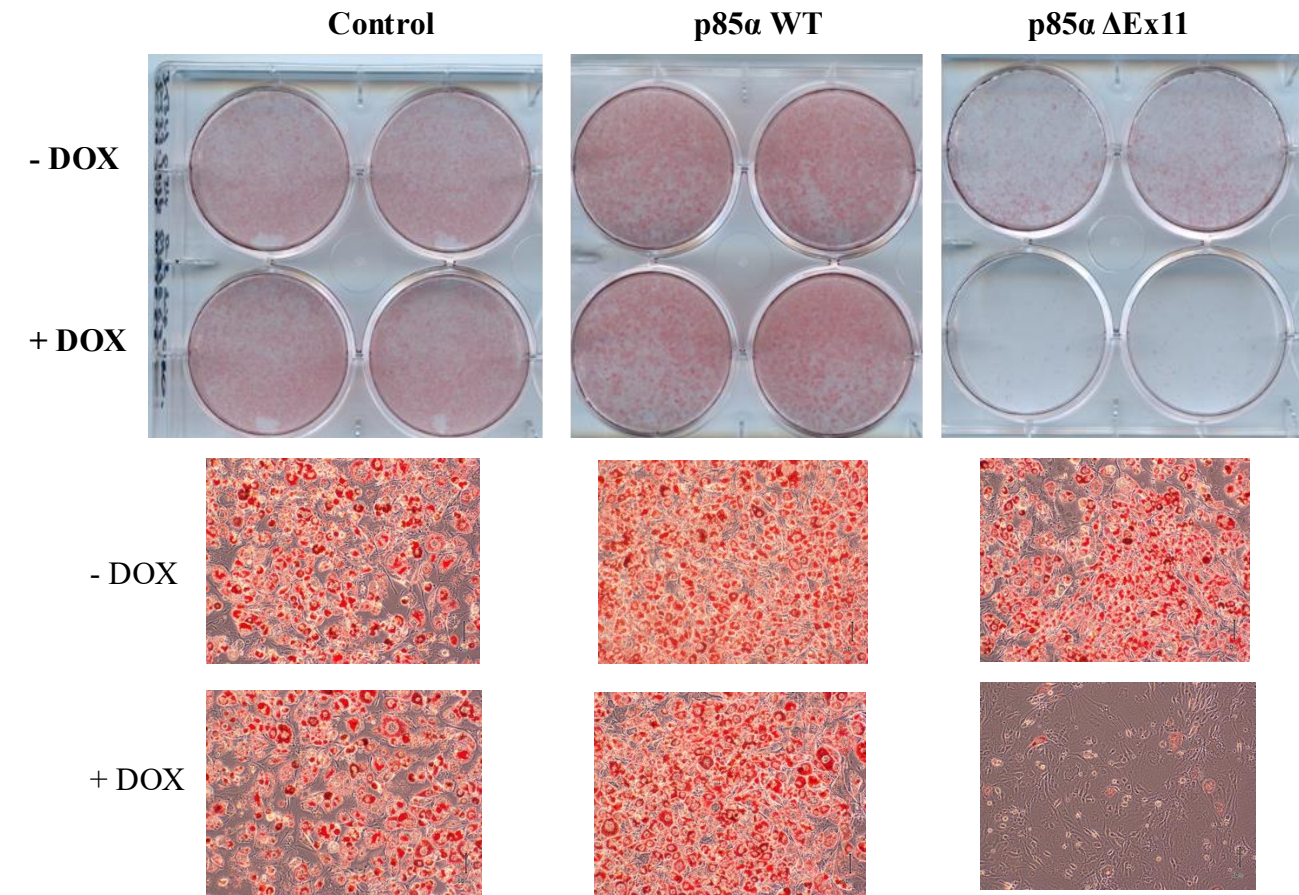

Figure 2D – Replicate 2

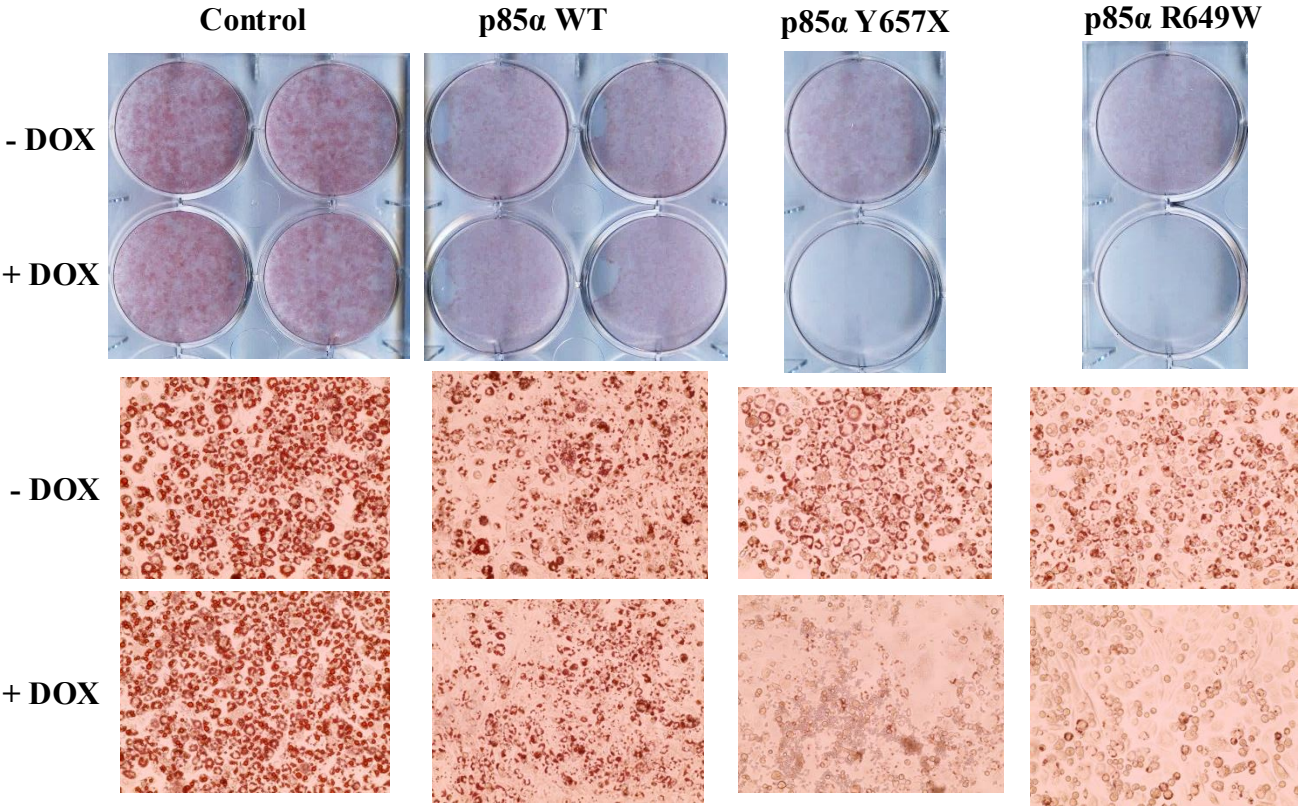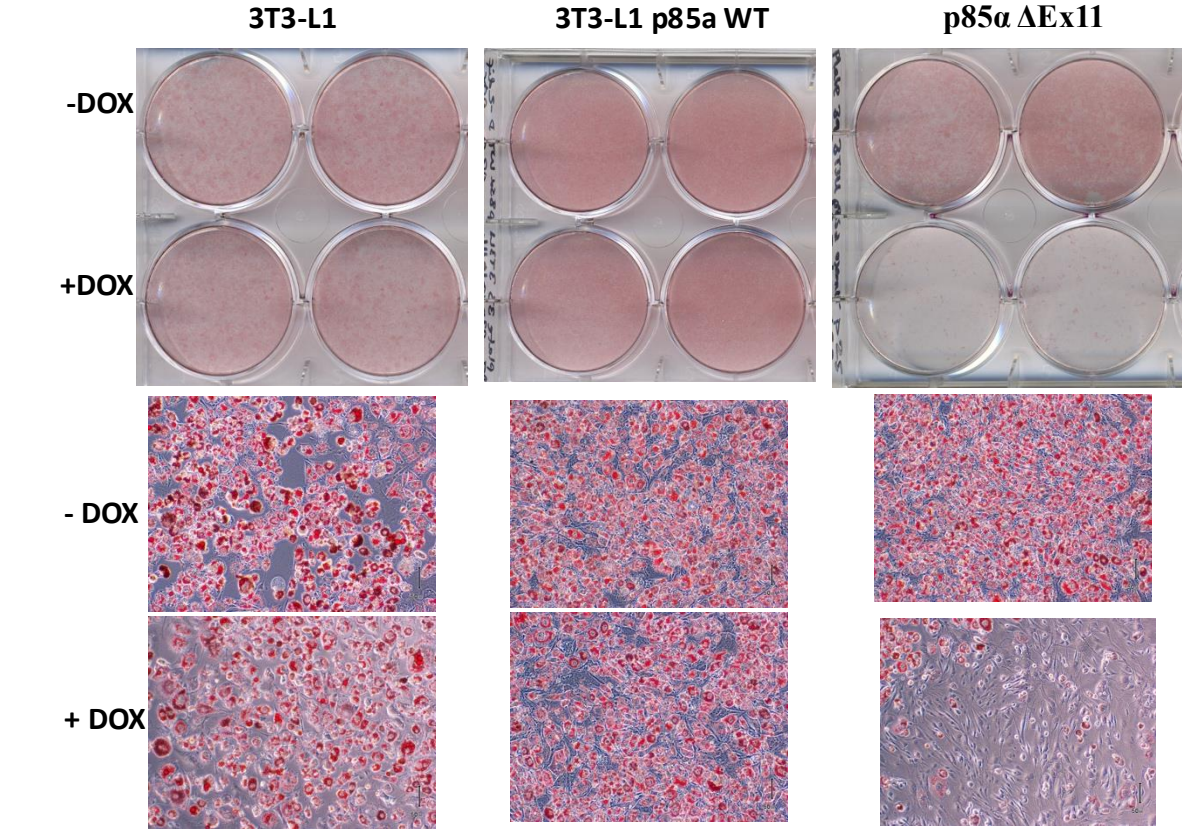

Supplement: Figure 2—source data 3. [file elife-94420-fig2-data3.pdf]

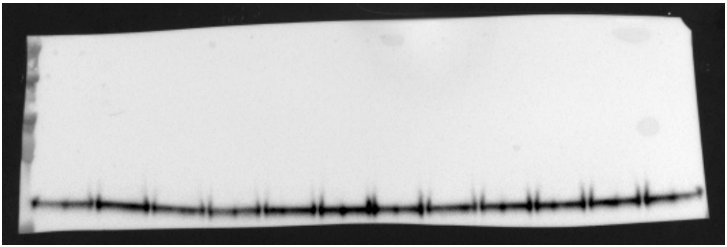

Supplement: Figure 2—figure supplement 3—source data 1. [file elife-94420-fig2-figsupp3-data1.zip › Figure 2-figure_supplement_3_source_data_1/Fig2-figure_supplement_3_p85alpha_WT_control_AKT_replicate_source_image.png]

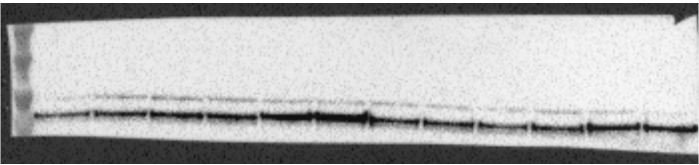

Supplement: Figure 2—figure supplement 3—source data 1. [file elife-94420-fig2-figsupp3-data1.zip › Figure 2-figure_supplement_3_source_data_1/Fig2-figure_supplement_3_p85alpha_WT_p85alpha_overexpression_p85alpha_replicate_source_image.png]

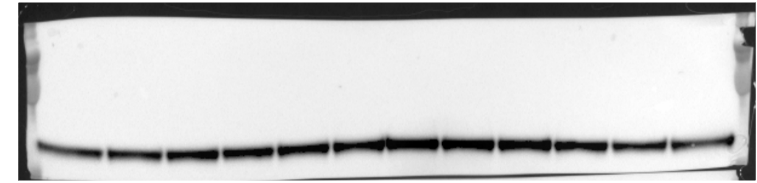

Supplement: Figure 2—figure supplement 3—source data 1. [file elife-94420-fig2-figsupp3-data1.zip › Figure 2-figure_supplement_3_source_data_1/Fig2-figure_supplement_3_p85alpha_deltaEx11_p85alpha_overexpression_AKT_source_image.png]

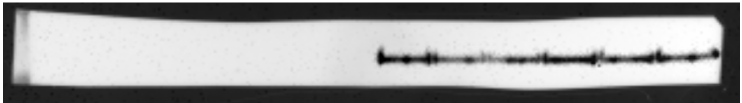

Supplement: Figure 2—figure supplement 3—source data 1. [file elife-94420-fig2-figsupp3-data1.zip › Figure 2-figure_supplement_3_source_data_1/Fig2-figure_supplement_3_p85alpha_control_pAKT_S473_replicate_source_image.png]

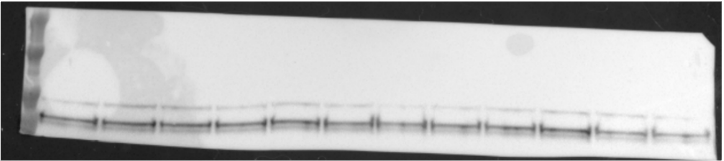

Supplement: Figure 2—figure supplement 3—source data 1. [file elife-94420-fig2-figsupp3-data1.zip › Figure 2-figure_supplement_3_source_data_1/Fig2-figure_supplement_3_p85alpha_control_p85alpha_replicate_source_image.png]

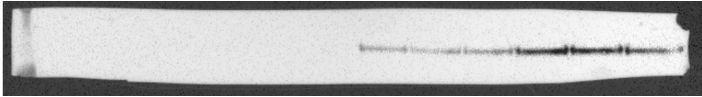

Supplement: Figure 2—figure supplement 3—source data 1. [file elife-94420-fig2-figsupp3-data1.zip › Figure 2-figure_supplement_3_source_data_1/Fig2-figure_supplement_3_p85alpha_WT_p85alpha_overexpression_pAKT_S473_replicate_source_image.png]

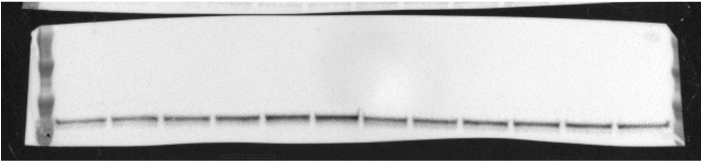

Supplement: Figure 2—figure supplement 3—source data 1. [file elife-94420-fig2-figsupp3-data1.zip › Figure 2-figure_supplement_3_source_data_1/Fig2-figure_supplement_3_p85alpha_WT_p85alpha_overexpression_p85alpha_source_image.png]

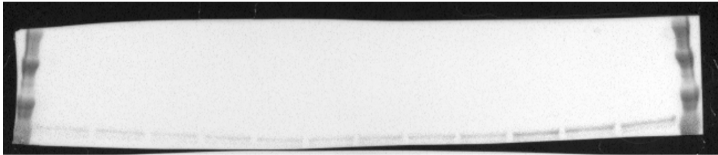

Supplement: Figure 2—figure supplement 3—source data 1. [file elife-94420-fig2-figsupp3-data1.zip › Figure 2-figure_supplement_3_source_data_1/Fig2-figure_supplement_3_p85alpha_control_source_image.png]

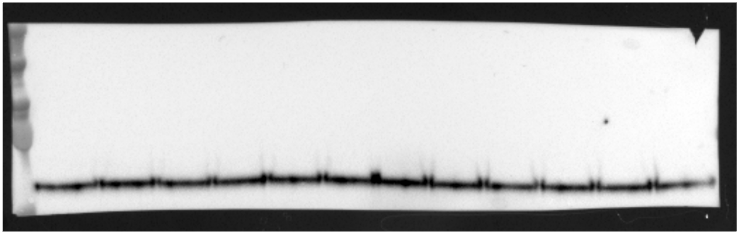

Supplement: Figure 2—figure supplement 3—source data 1. [file elife-94420-fig2-figsupp3-data1.zip › Figure 2-figure_supplement_3_source_data_1/Fig2-figure_supplement_3_p85alpha_WT_p85alpha_overexpression_AKT_replicate_source_image.png]

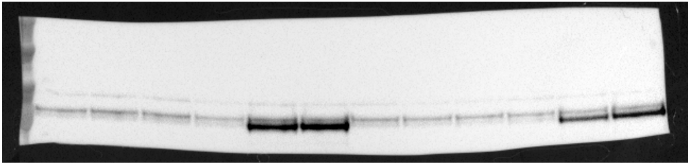

Supplement: Figure 2—figure supplement 3—source data 1. [file elife-94420-fig2-figsupp3-data1.zip › Figure 2-figure_supplement_3_source_data_1/Fig2-figure_supplement_3_p85alpha_deltaEx11_p85alpha_overexpression_p85alpha_replicate_source_image.png]

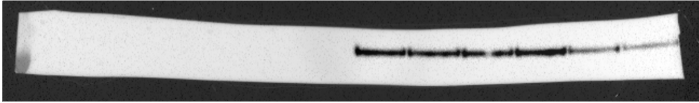

Supplement: Figure 2—figure supplement 3—source data 1. [file elife-94420-fig2-figsupp3-data1.zip › Figure 2-figure_supplement_3_source_data_1/Fig2-figure_supplement_3_p85alpha_deltaEx11_p85alpha_overexpression_pAKT_S473_replicate_source_image.png]

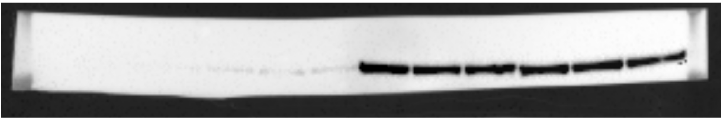

Supplement: Figure 2—figure supplement 3—source data 1. [file elife-94420-fig2-figsupp3-data1.zip › Figure 2-figure_supplement_3_source_data_1/Fig2-figure_supplement_3_pAKT_S473_control_source_image.png]

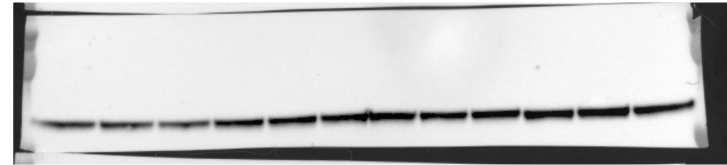

Supplement: Figure 2—figure supplement 3—source data 1. [file elife-94420-fig2-figsupp3-data1.zip › Figure 2-figure_supplement_3_source_data_1/Fig2-figure_supplement_3_AKT_control_source_image.png]

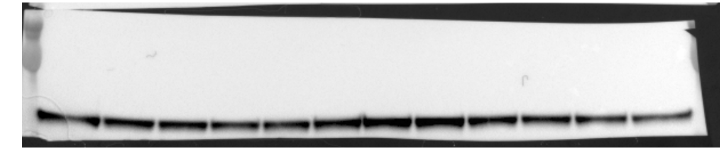

Supplement: Figure 2—figure supplement 3—source data 1. [file elife-94420-fig2-figsupp3-data1.zip › Figure 2-figure_supplement_3_source_data_1/Fig2-figure_supplement_3_p85alpha_WT_p85alpha_overexpression_AKT_source_image.png]

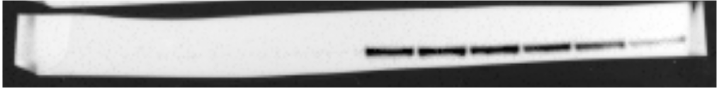

Supplement: Figure 2—figure supplement 3—source data 1. [file elife-94420-fig2-figsupp3-data1.zip › Figure 2-figure_supplement_3_source_data_1/Fig2-figure_supplement_3_p85alpha_deltaEx11_p85alpha_overexpression_pAKT_S473_source_image.png]

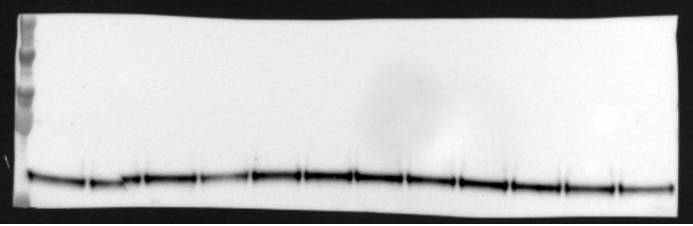

Supplement: Figure 2—figure supplement 3—source data 1. [file elife-94420-fig2-figsupp3-data1.zip › Figure 2-figure_supplement_3_source_data_1/Fig2-figure_supplement_3_p85alpha_deltaEx11_p85alpha_overexpression_AKT_replicate_source_image.png]

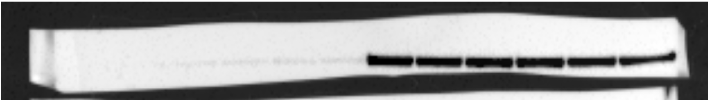

Supplement: Figure 2—figure supplement 3—source data 1. [file elife-94420-fig2-figsupp3-data1.zip › Figure 2-figure_supplement_3_source_data_1/Fig2-figure_supplement_3_p85alpha_WT_p85alpha_overexpression_pAKT_S473_source_image.png]

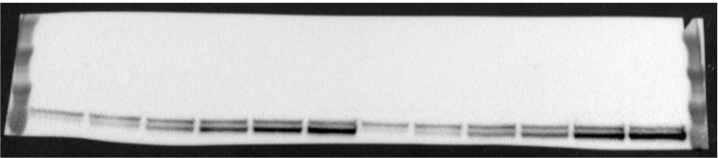

Supplement: Figure 2—figure supplement 3—source data 1. [file elife-94420-fig2-figsupp3-data1.zip › Figure 2-figure_supplement_3_source_data_1/Fig2-figure_supplement_3_p85alpha_deltaEx11_p85alpha_overexpression_p85alpha_source_image.png]

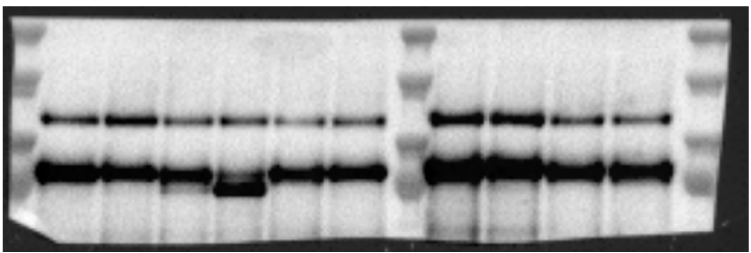

Supplement: Figure 4—source data 1. [file elife-94420-fig4-data1.zip › Figure_4_source_data_1/Fig4_IP_p110alpha_replicate_2_source_image.png]

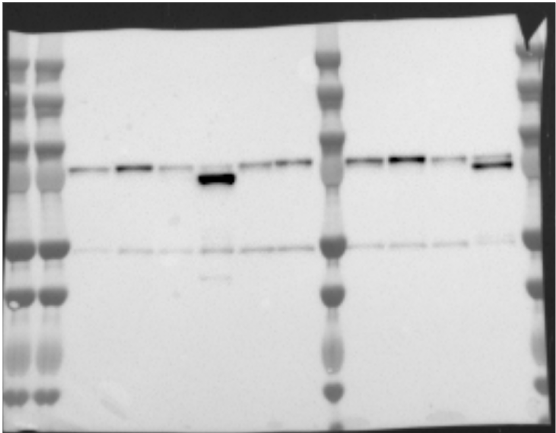

Supplement: Figure 4—source data 1. [file elife-94420-fig4-data1.zip › Figure_4_source_data_1/Fig4_supernatant_p85alpha_replicate_1_source_image.png]

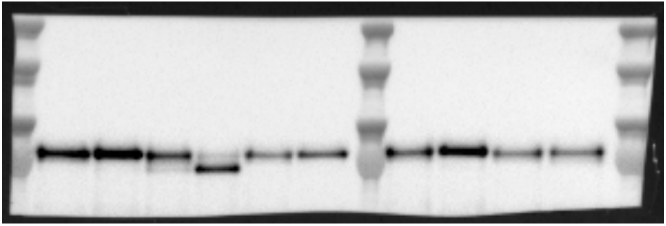

Supplement: Figure 4—source data 1. [file elife-94420-fig4-data1.zip › Figure_4_source_data_1/Fig4_IP_p85alpha_replicate_2_source_image.png]

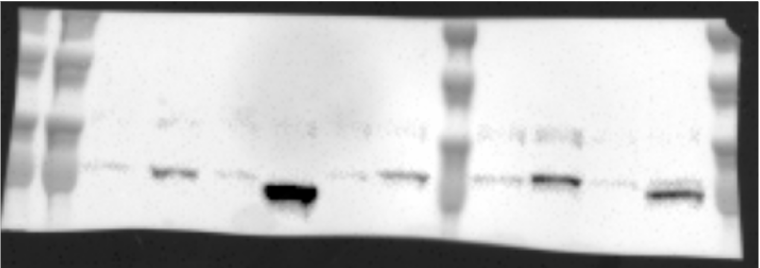

Supplement: Figure 4—source data 1. [file elife-94420-fig4-data1.zip › Figure_4_source_data_1/Fig4_lysate_p110alpha_replicate_1_source_image.png]

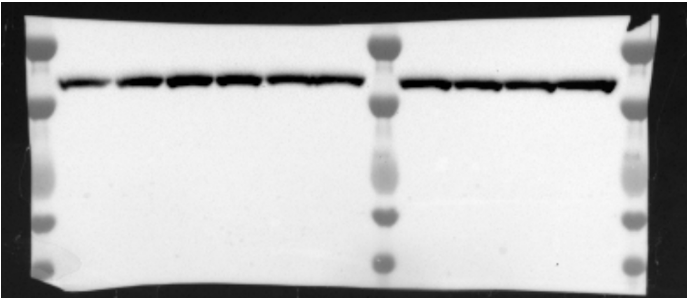

Supplement: Figure 4—source data 1. [file elife-94420-fig4-data1.zip › Figure_4_source_data_1/Fig4_lysate_beta_actin_source_image.png]

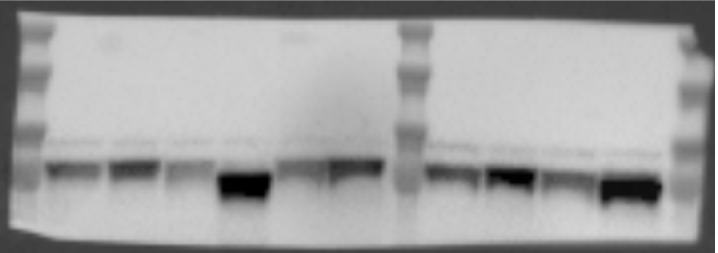

Supplement: Figure 4—source data 1. [file elife-94420-fig4-data1.zip › Figure_4_source_data_1/Fig4_lysate_p85alpha_source_image.png]

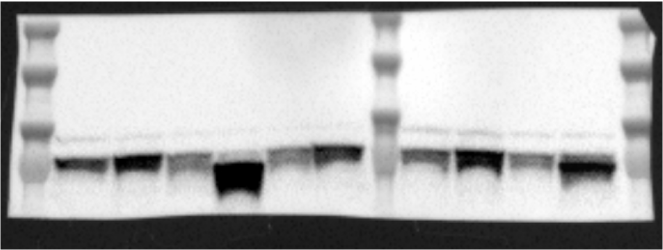

Supplement: Figure 4—source data 1. [file elife-94420-fig4-data1.zip › Figure_4_source_data_1/Fig4_lysate_p85alpha_replicate_2_source_image.png]

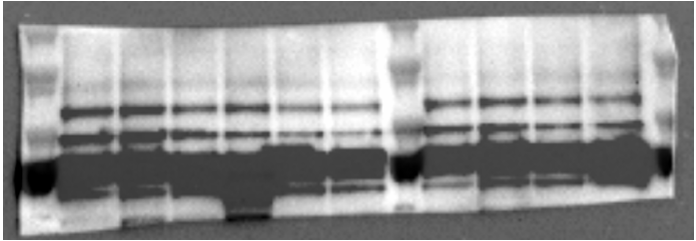

Supplement: Figure 4—source data 1. [file elife-94420-fig4-data1.zip › Figure_4_source_data_1/Fig4_supernatant_p110alpha_replicate_2_source_image.png]

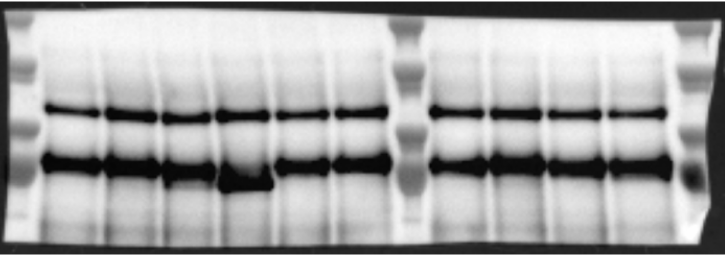

Supplement: Figure 4—source data 1. [file elife-94420-fig4-data1.zip › Figure_4_source_data_1/Fig4_IP_p110alpha_source_image.png]

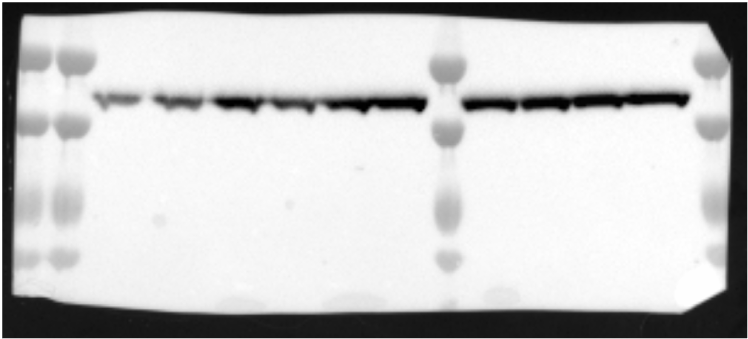

Supplement: Figure 4—source data 1. [file elife-94420-fig4-data1.zip › Figure_4_source_data_1/Fig4_lysate_beta_actin_replicate_1_source_image.png]

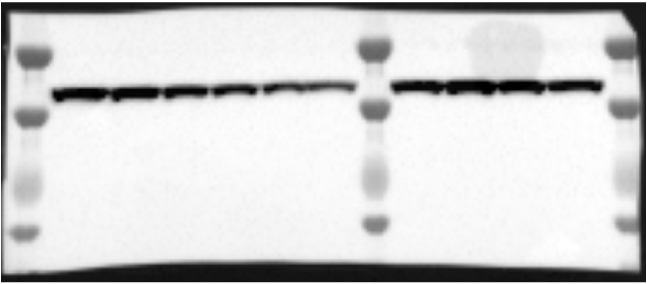

Supplement: Figure 4—source data 1. [file elife-94420-fig4-data1.zip › Figure_4_source_data_1/Fig4_supernatant_beta_actin_replicate_2_source_image.png]

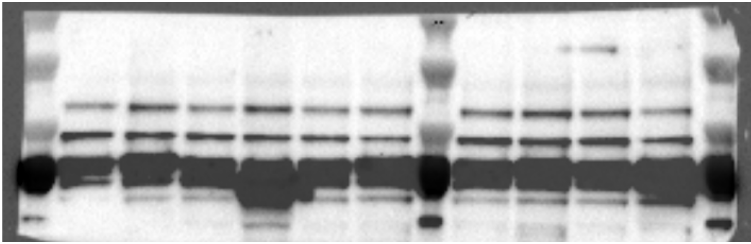

Supplement: Figure 4—source data 1. [file elife-94420-fig4-data1.zip › Figure_4_source_data_1/Fig4_supernatant_p110alpha_source_image.png]

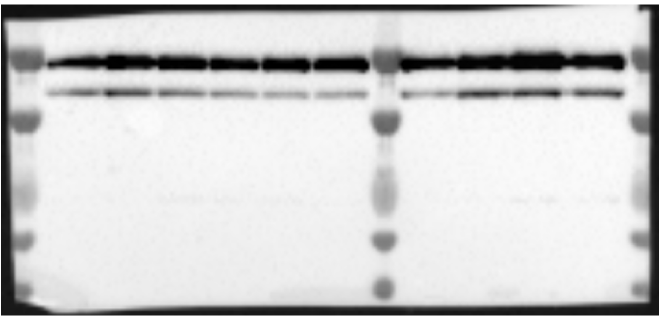

Supplement: Figure 4—source data 1. [file elife-94420-fig4-data1.zip › Figure_4_source_data_1/Fig4_lysate_beta_actin_replicate_2_source_image.png]

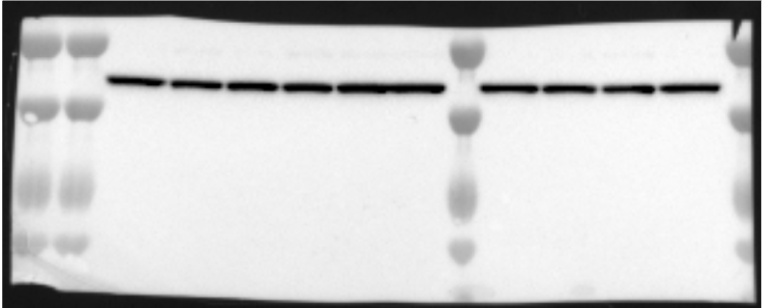

Supplement: Figure 4—source data 1. [file elife-94420-fig4-data1.zip › Figure_4_source_data_1/Fig4_supernatant_beta_actin_replicate_1_source_image.png]

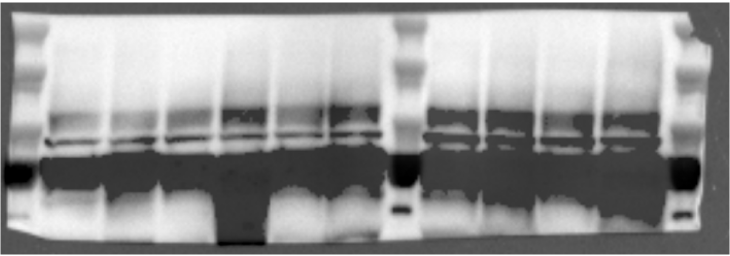

Supplement: Figure 4—source data 1. [file elife-94420-fig4-data1.zip › Figure_4_source_data_1/Fig4_lysate_p110alpha_source_image.png]

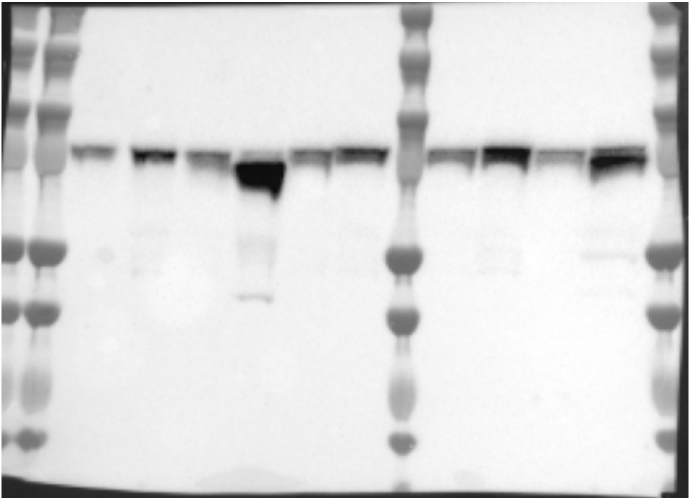

Supplement: Figure 4—source data 1. [file elife-94420-fig4-data1.zip › Figure_4_source_data_1/Fig4_lysate_p85alpha_replicate_1_source_image.png]

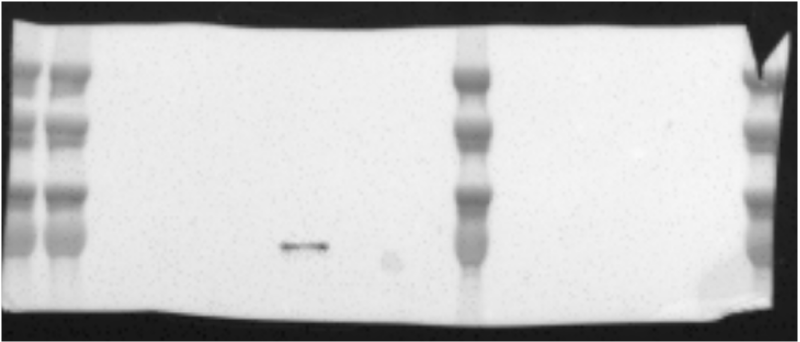

Supplement: Figure 4—source data 1. [file elife-94420-fig4-data1.zip › Figure_4_source_data_1/Fig4_supernatant_p110alpha_replicate_1_source_image.png]

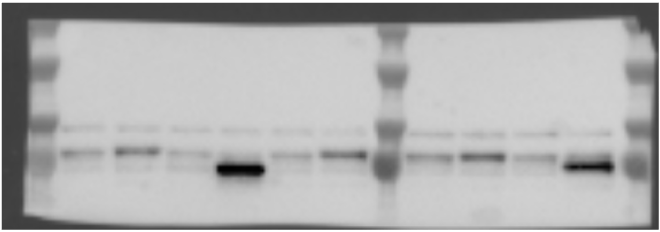

Supplement: Figure 4—source data 1. [file elife-94420-fig4-data1.zip › Figure_4_source_data_1/Fig4_supernatant_p85alpha_source_image.png]

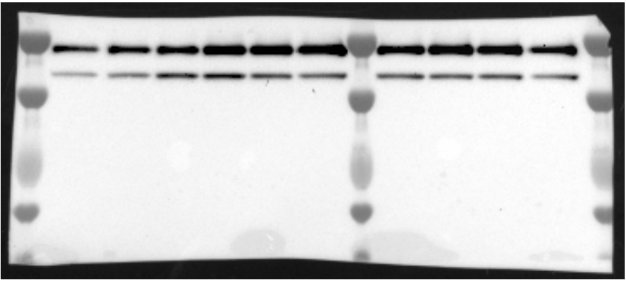

Supplement: Figure 4—source data 1. [file elife-94420-fig4-data1.zip › Figure_4_source_data_1/Fig4_supernatant_beta_actin_source_image.png]

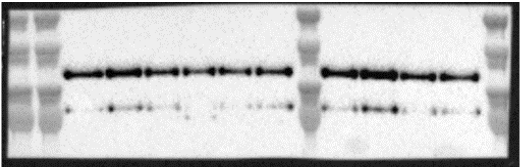

Supplement: Figure 4—source data 1. [file elife-94420-fig4-data1.zip › Figure_4_source_data_1/Fig4_IP_p110alpha_replicate_1_source_image.png]

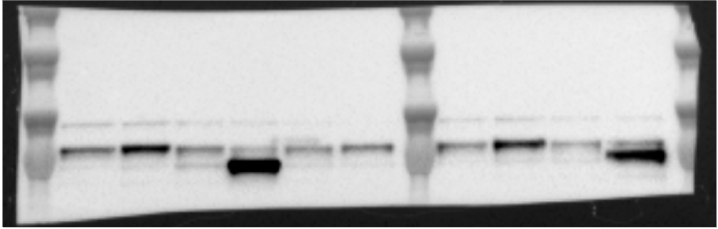

Supplement: Figure 4—source data 1. [file elife-94420-fig4-data1.zip › Figure_4_source_data_1/Fig4_supernatant_p85alpha_replicate_2_source_image.png]

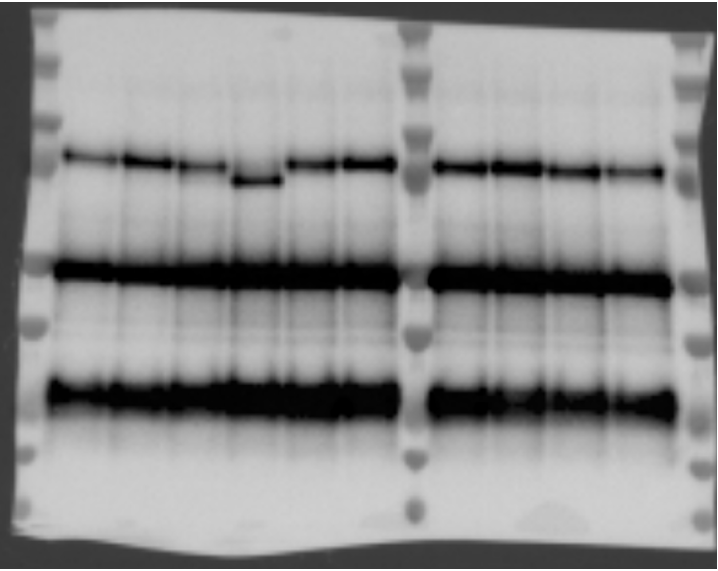

Supplement: Figure 4—source data 1. [file elife-94420-fig4-data1.zip › Figure_4_source_data_1/Fig4_IP_p85alpha_source_image.png]

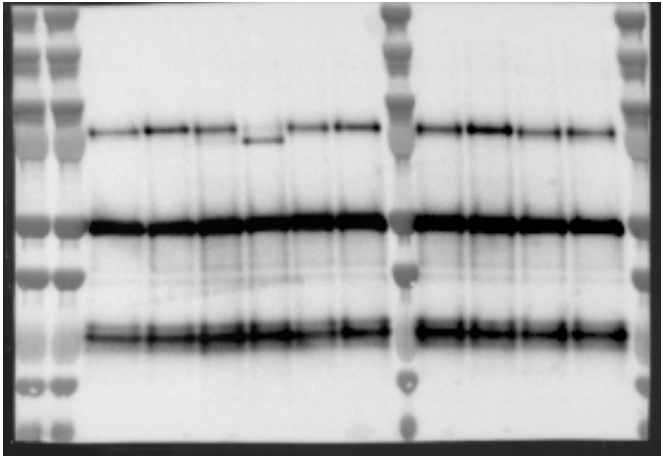

Supplement: Figure 4—source data 1. [file elife-94420-fig4-data1.zip › Figure_4_source_data_1/Fig4_IP_p85alpha_replicate_1_source_image.png]

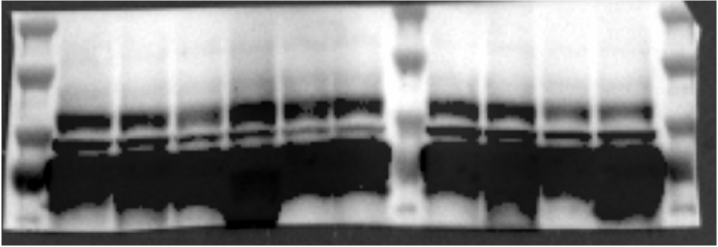

Supplement: Figure 4—source data 1. [file elife-94420-fig4-data1.zip › Figure_4_source_data_1/Fig4_lysate_p110alpha_replicate_2_source_image.png]

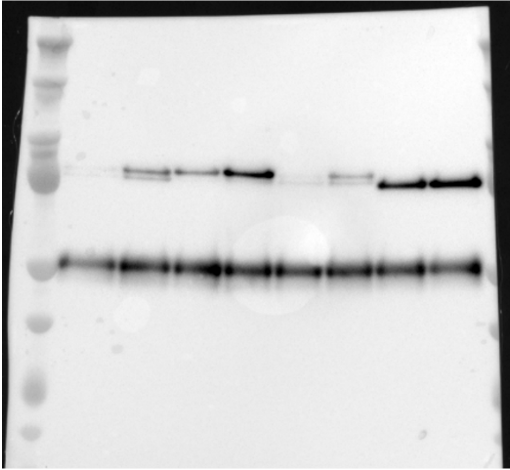

Supplement: Figure 5—source data 1. [file elife-94420-fig5-data1.zip › Figure_5_source_data_1/Fig5A_IP_p85alpha_replicate_2_source_image_2.png]

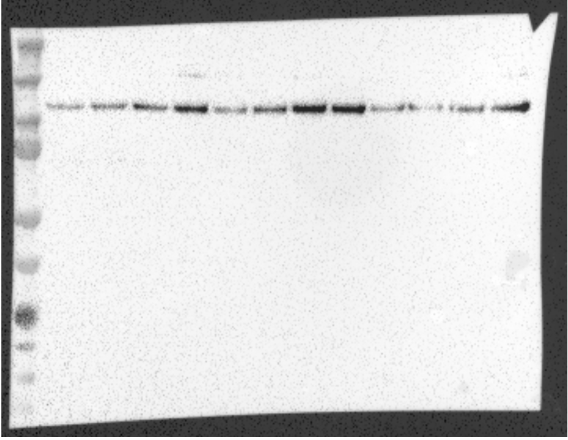

Supplement: Figure 5—source data 1. [file elife-94420-fig5-data1.zip › Figure_5_source_data_1/Fig5A_lysate_p110alpha_source_image_1.png]

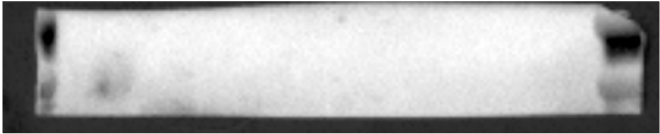

Supplement: Figure 5—source data 1. [file elife-94420-fig5-data1.zip › Figure_5_source_data_1/Fig5A_supernatant_IRS1_source_image_2.png]

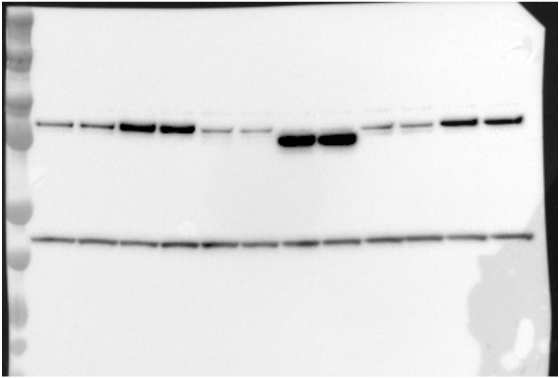

Supplement: Figure 5—source data 1. [file elife-94420-fig5-data1.zip › Figure_5_source_data_1/Fig5A_lysate_p85alpha_replicate_2_source_image_1.png]

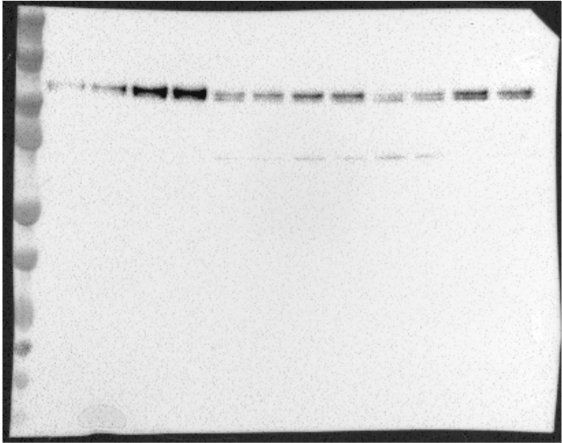

Supplement: Figure 5—source data 1. [file elife-94420-fig5-data1.zip › Figure_5_source_data_1/Fig5A_lysate_p110alpha_replicate_2_source_image_1.png]

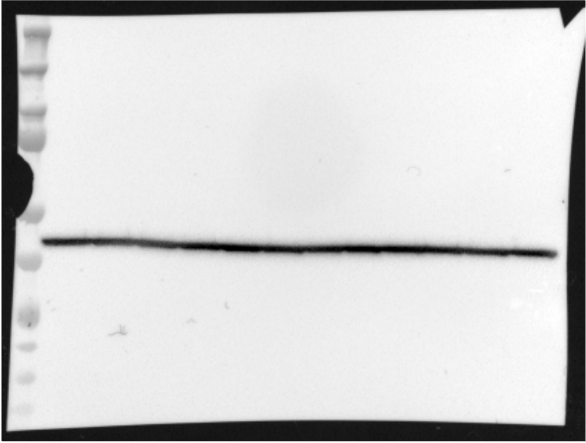

Supplement: Figure 5—source data 1. [file elife-94420-fig5-data1.zip › Figure_5_source_data_1/Fig5A_lysate_beta_actin_source_image_1.png]

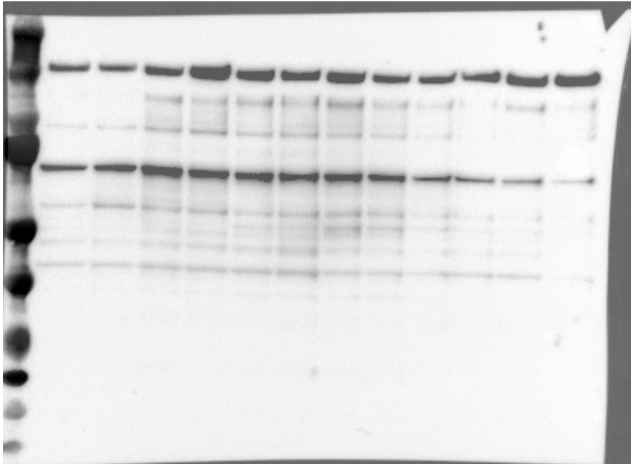

Supplement: Figure 5—source data 1. [file elife-94420-fig5-data1.zip › Figure_5_source_data_1/Fig5A_lysate_IRS1_source_image_1.png]

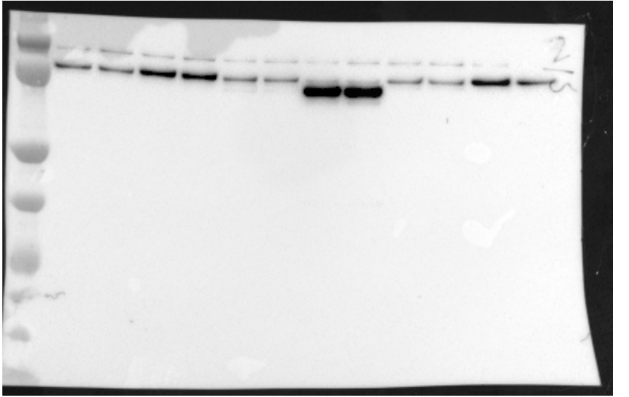

Supplement: Figure 5—source data 1. [file elife-94420-fig5-data1.zip › Figure_5_source_data_1/Fig5A_supernatant_p85alpha_source_image_1.png]

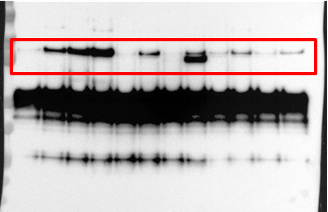

Supplement: Figure 5—source data 1. [file elife-94420-fig5-data1.zip › Figure_5_source_data_1/Fig5A_IP_p85alpha_replicate_2_source_image_1.png]

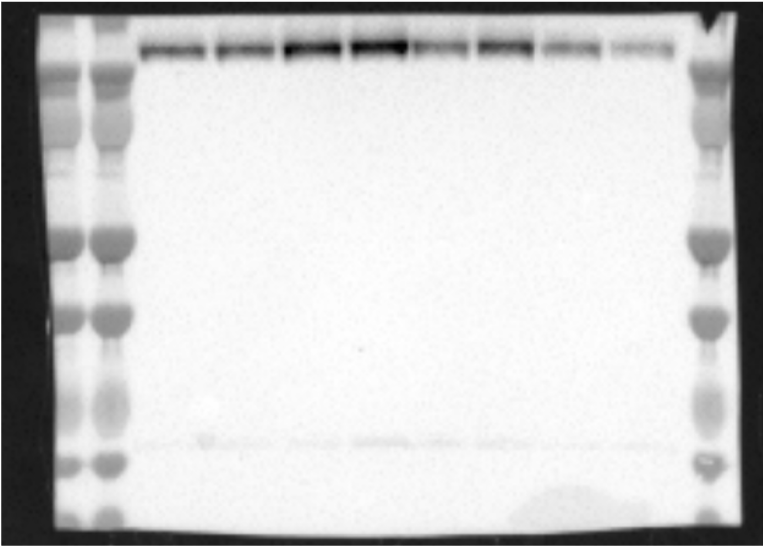

Supplement: Figure 5—source data 1. [file elife-94420-fig5-data1.zip › Figure_5_source_data_1/Fig5A_lysate_p110alpha_source_image_2.png]

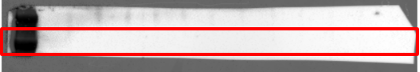

Supplement: Figure 5—source data 1. [file elife-94420-fig5-data1.zip › Figure_5_source_data_1/Fig5A_supernatant_IRS1_source_image_1.png]

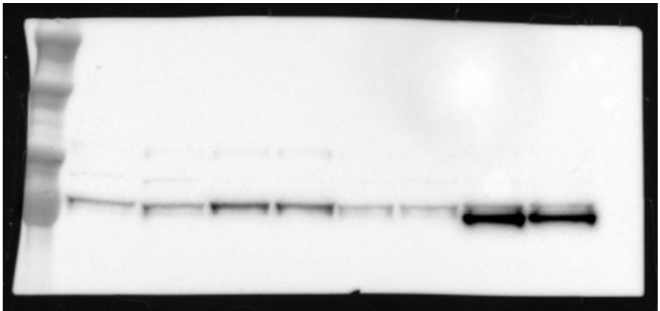

Supplement: Figure 5—source data 1. [file elife-94420-fig5-data1.zip › Figure_5_source_data_1/Fig5A_lysate_p85alpha_replicate_2_source_image_2.png]

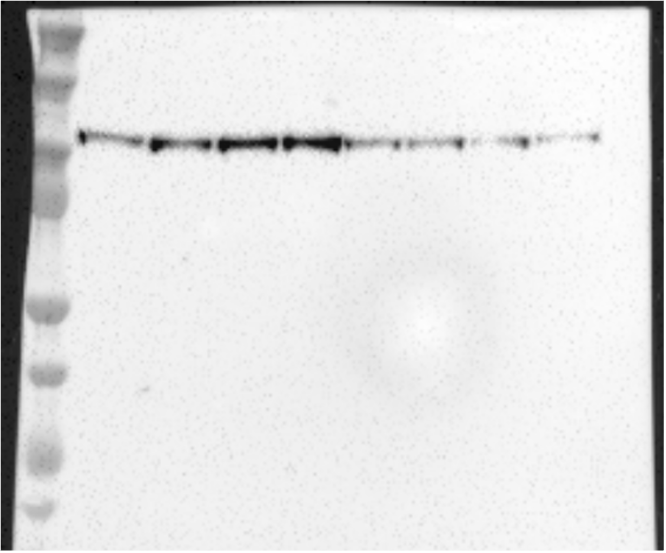

Supplement: Figure 5—source data 1. [file elife-94420-fig5-data1.zip › Figure_5_source_data_1/Fig5A_lysate_p110alpha_replicate_2_source_image_2.png]

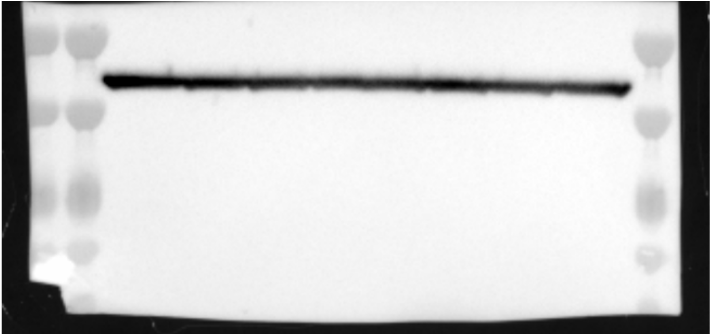

Supplement: Figure 5—source data 1. [file elife-94420-fig5-data1.zip › Figure_5_source_data_1/Fig5A_lysate_beta_actin_source_image_2.png]

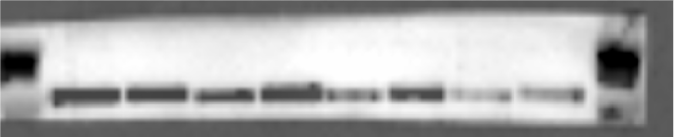

Supplement: Figure 5—source data 1. [file elife-94420-fig5-data1.zip › Figure_5_source_data_1/Fig5A_lysate_IRS1_source_image_2.png]

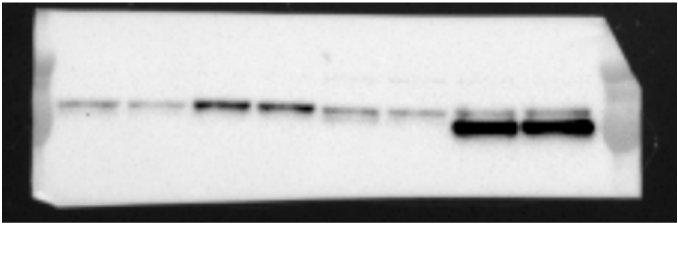

Supplement: Figure 5—source data 1. [file elife-94420-fig5-data1.zip › Figure_5_source_data_1/Fig5A_supernatant_p85alpha_source_image_2.png]

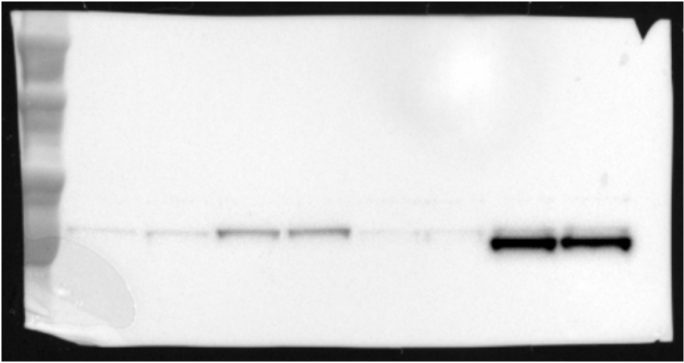

Supplement: Figure 5—source data 1. [file elife-94420-fig5-data1.zip › Figure_5_source_data_1/Fig5A_lysate_p85alpha_replicate_1_source_image_2.png]

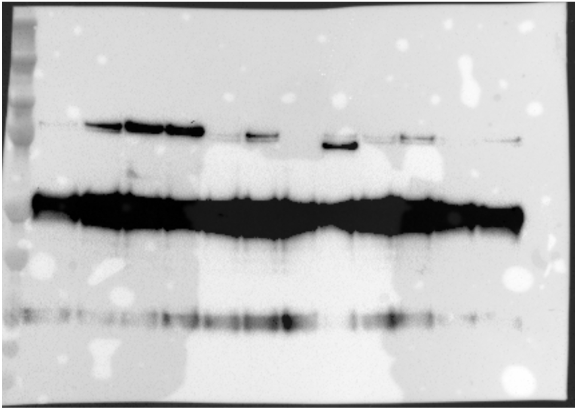

Supplement: Figure 5—source data 1. [file elife-94420-fig5-data1.zip › Figure_5_source_data_1/Fig5A_IP_p85alpha_replicate_1_source_image_1.png]

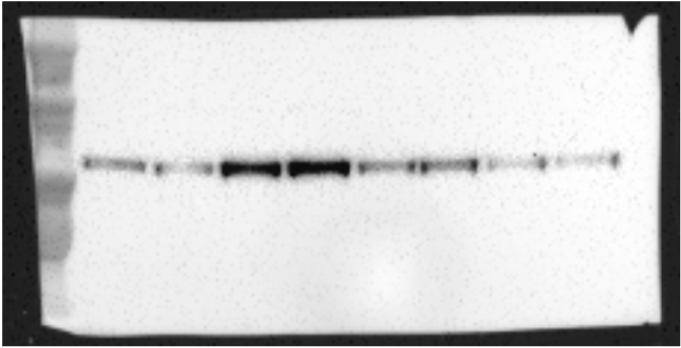

Supplement: Figure 5—source data 1. [file elife-94420-fig5-data1.zip › Figure_5_source_data_1/Fig5A_lysate_p110alpha_replicate_1_source_image_2.png]

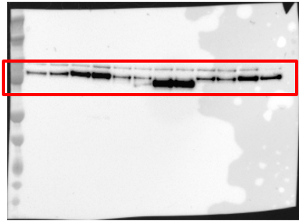

Supplement: Figure 5—source data 1. [file elife-94420-fig5-data1.zip › Figure_5_source_data_1/Fig5A_lysate_p85alpha_replicate_1_source_image_1.png]

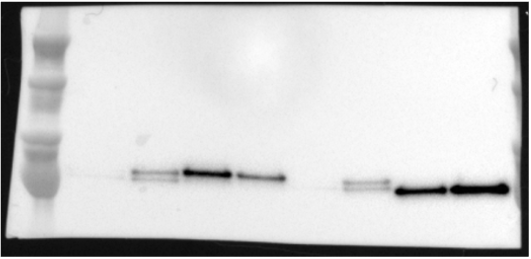

Supplement: Figure 5—source data 1. [file elife-94420-fig5-data1.zip › Figure_5_source_data_1/Fig5A_IP_p85alpha_replicate_1_source_image_2.png]
