# Supplementary material for: Paradoxical dominant negative activity of an immunodeficiency-associated activating PIK3R1 variant
Source: eLife. 2025 Jan 21;13:RP94420. doi: 10.7554/eLife.94420 (PMC11750134; doi:10.7554/eLife.94420)

Figure 1A and Figure 1-figure supplement 2B,C – Images shown

pAkt (S473/4)

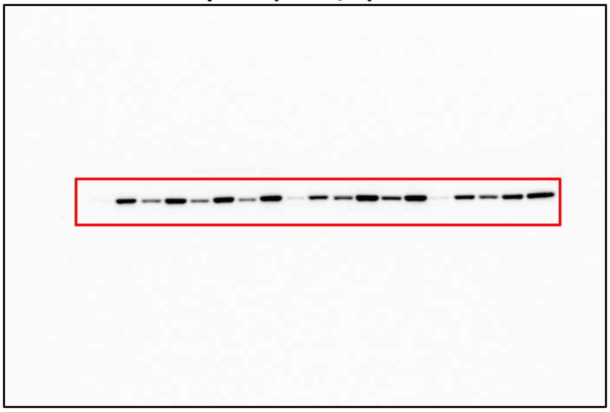

pAkt (T308/9)

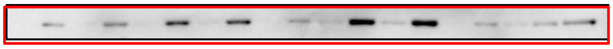

Akt

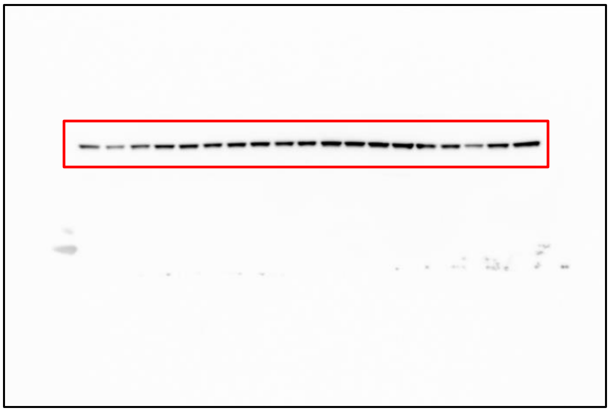

p85α

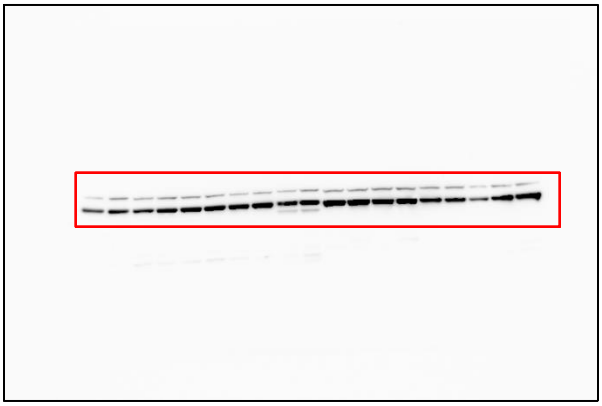

p110α

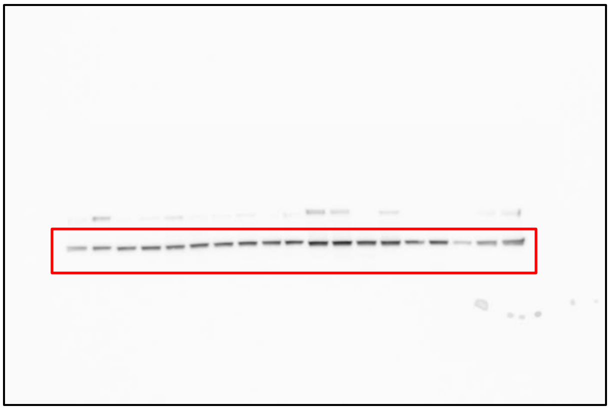

p110δ

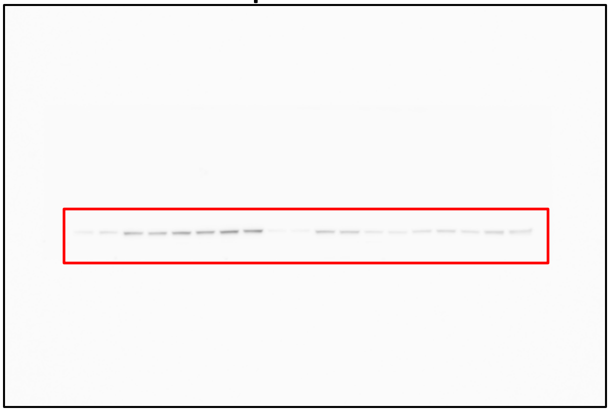

β-actin

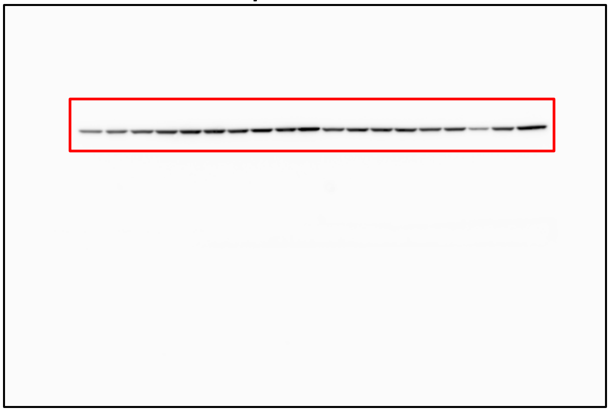

Figure 1A and Figure 1-figure supplement 2B,C – Replicate 1

pAkt (S473/4)

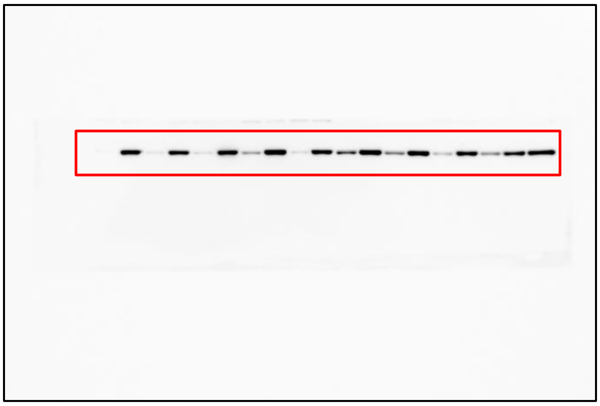

pAkt (T308/9)

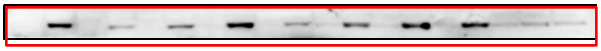

Akt

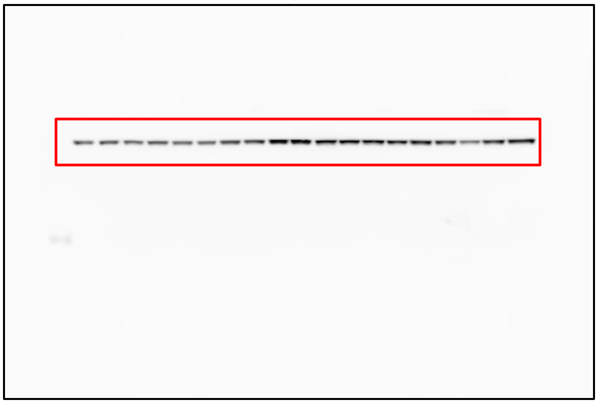

p85α

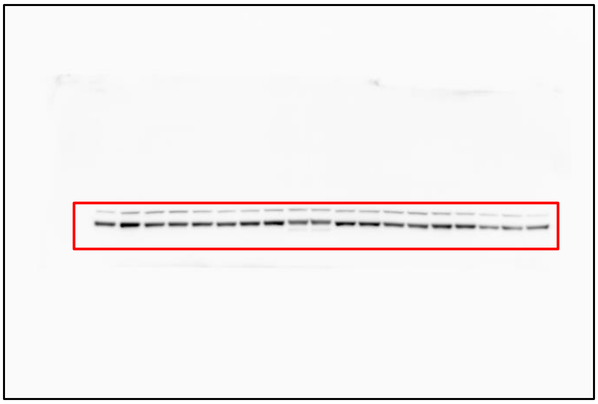

p110α

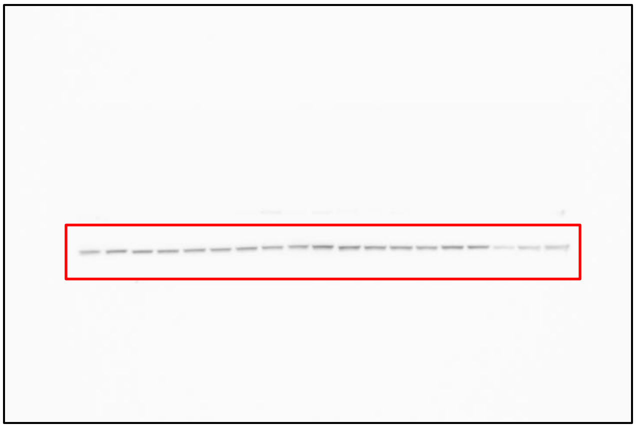

p110δ

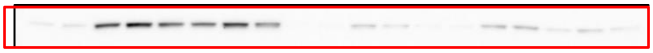

β-actin

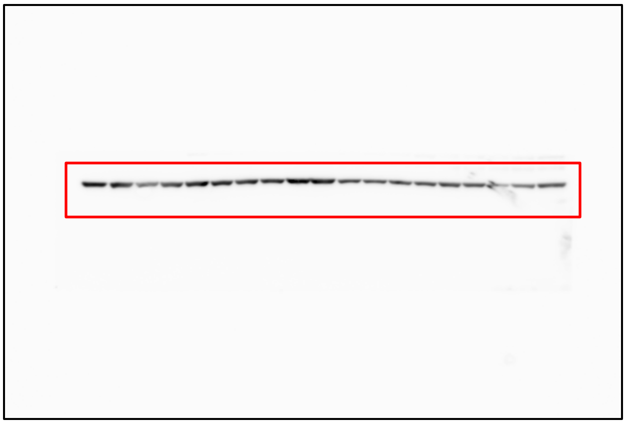

Figure 1A and Figure 1-figure supplement 2B,C – Replicate 2

pAkt (S473/4)

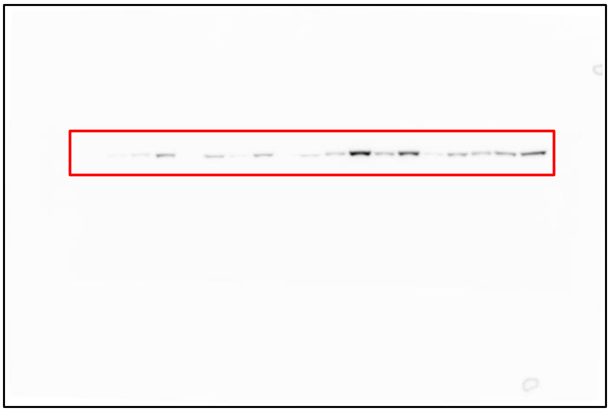

pAkt (T308/9)

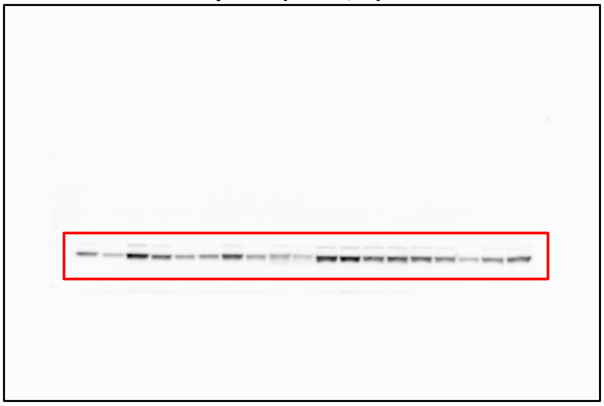

Akt

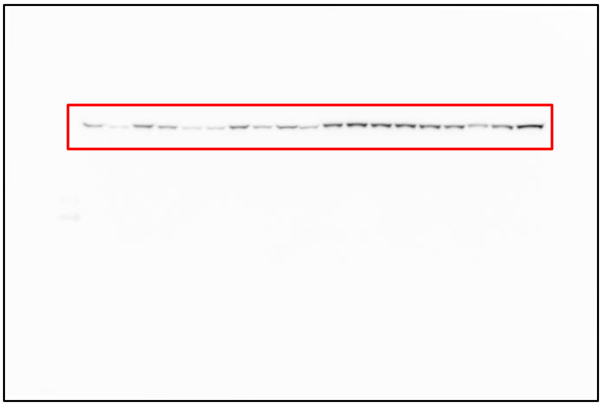

p85α

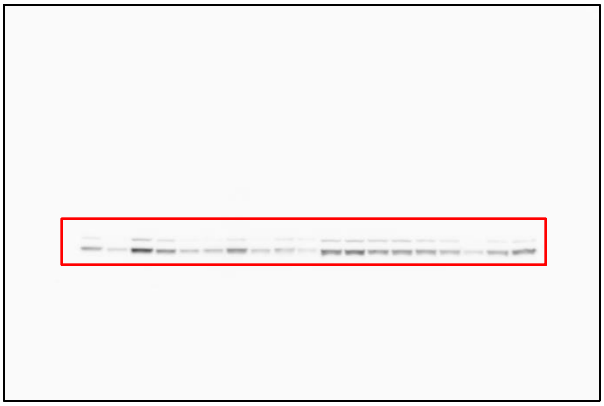

p110α

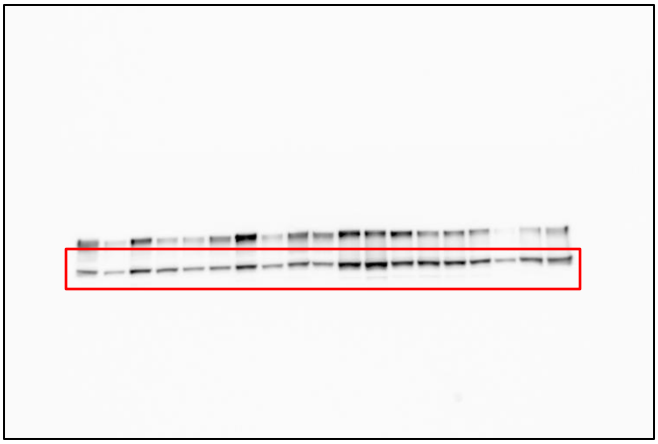

p110δ

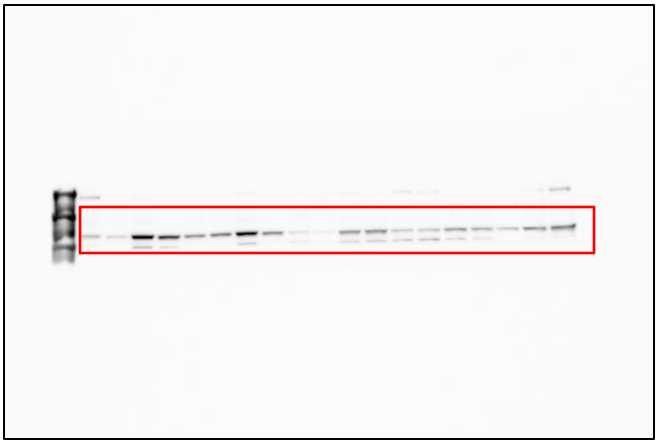

β-actin

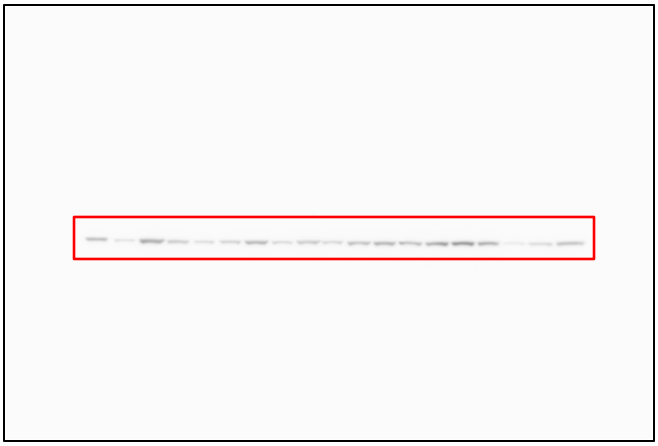

Supplement: Figure 1—source data 2. [file elife-94420-fig1-data2.pdf]
