# Supplementary material for: Paradoxical dominant negative activity of an immunodeficiency-associated activating PIK3R1 variant
Source: eLife. 2025 Jan 21;13:RP94420. doi: 10.7554/eLife.94420 (PMC11750134; doi:10.7554/eLife.94420)

Figure 2A – Images shown

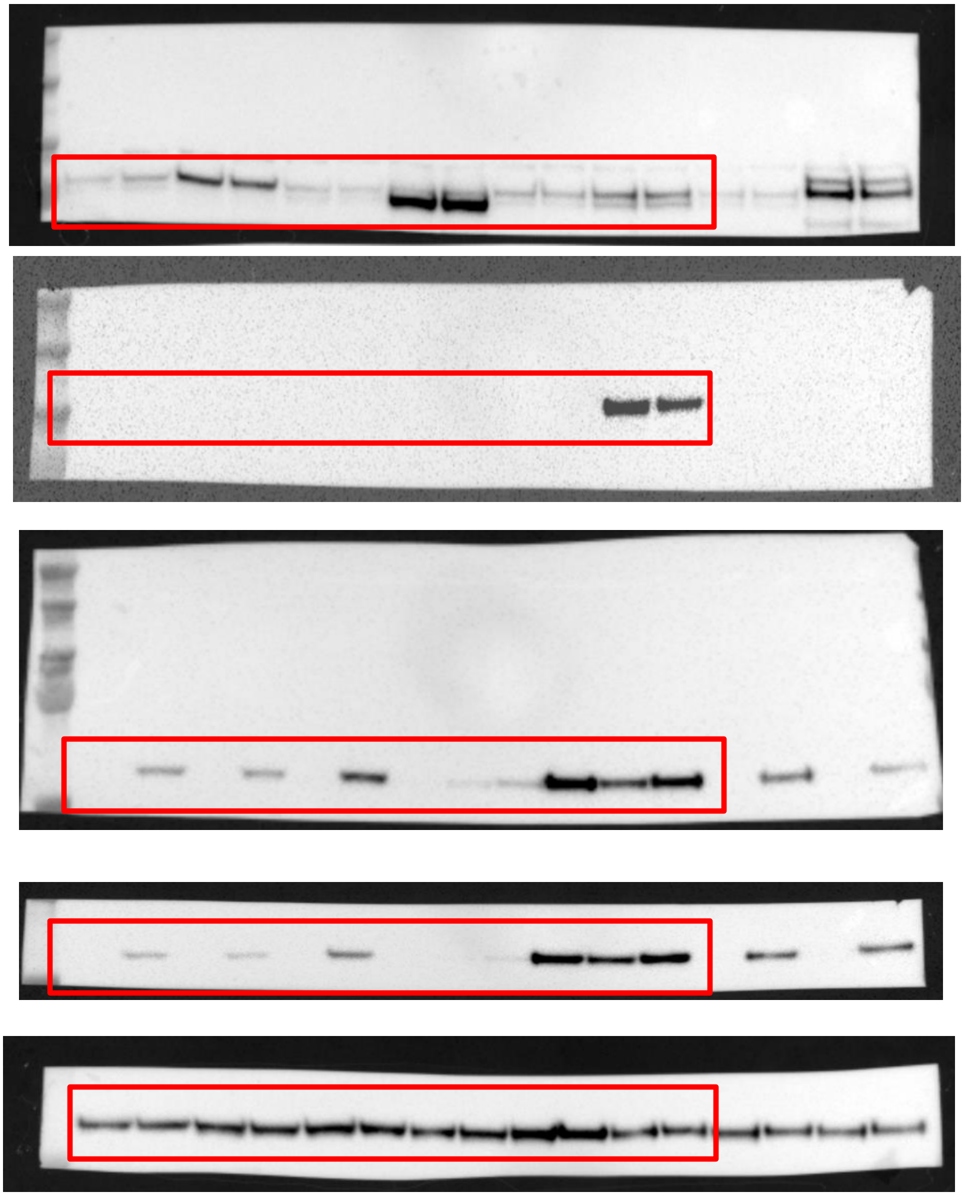

Total p85α

p110α

pAkt (Ser473)

pAkt (Thr308)

Total Akt

Figure 2A – Replicate 1

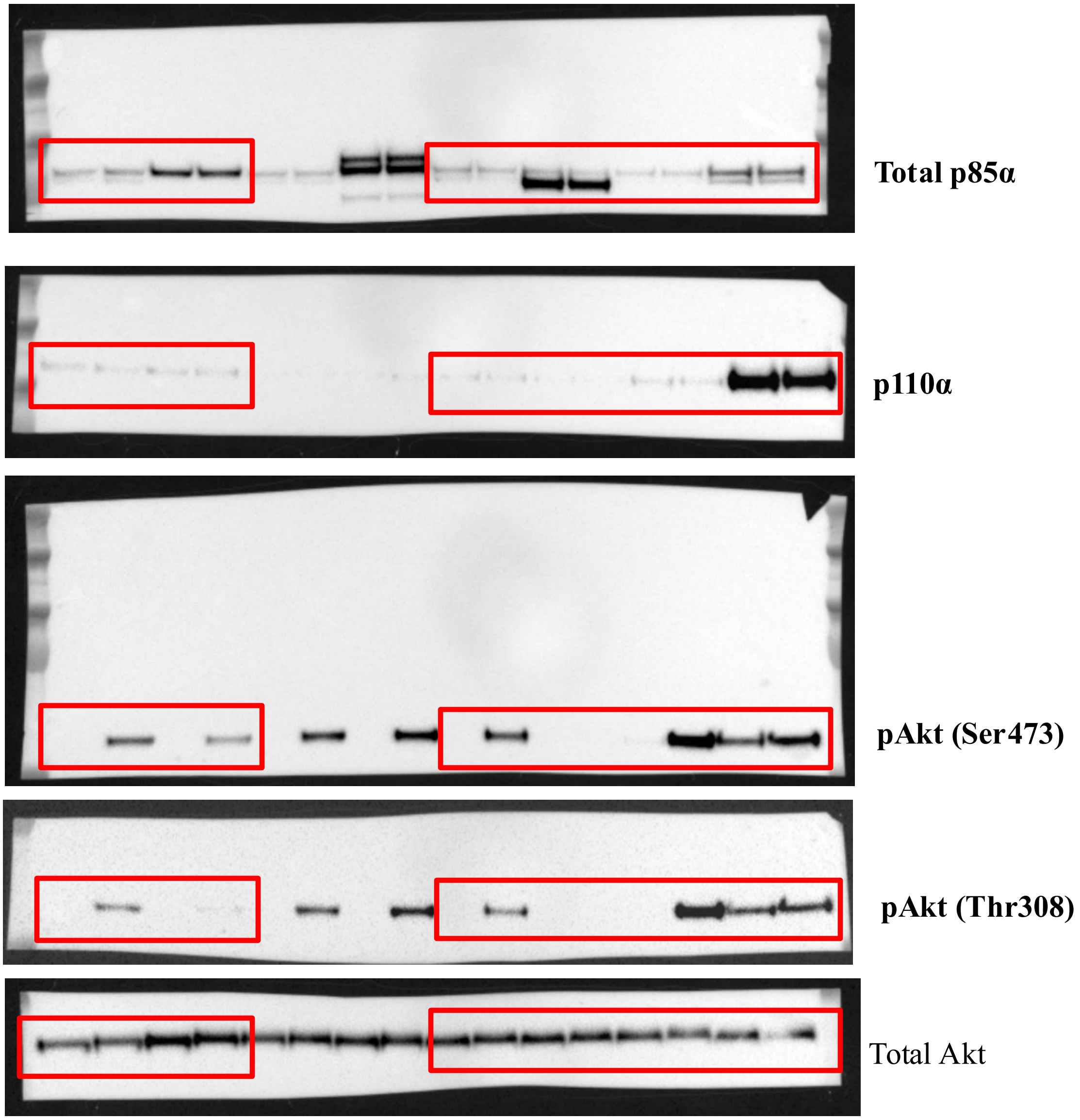

Figure 2A – Replicate 2

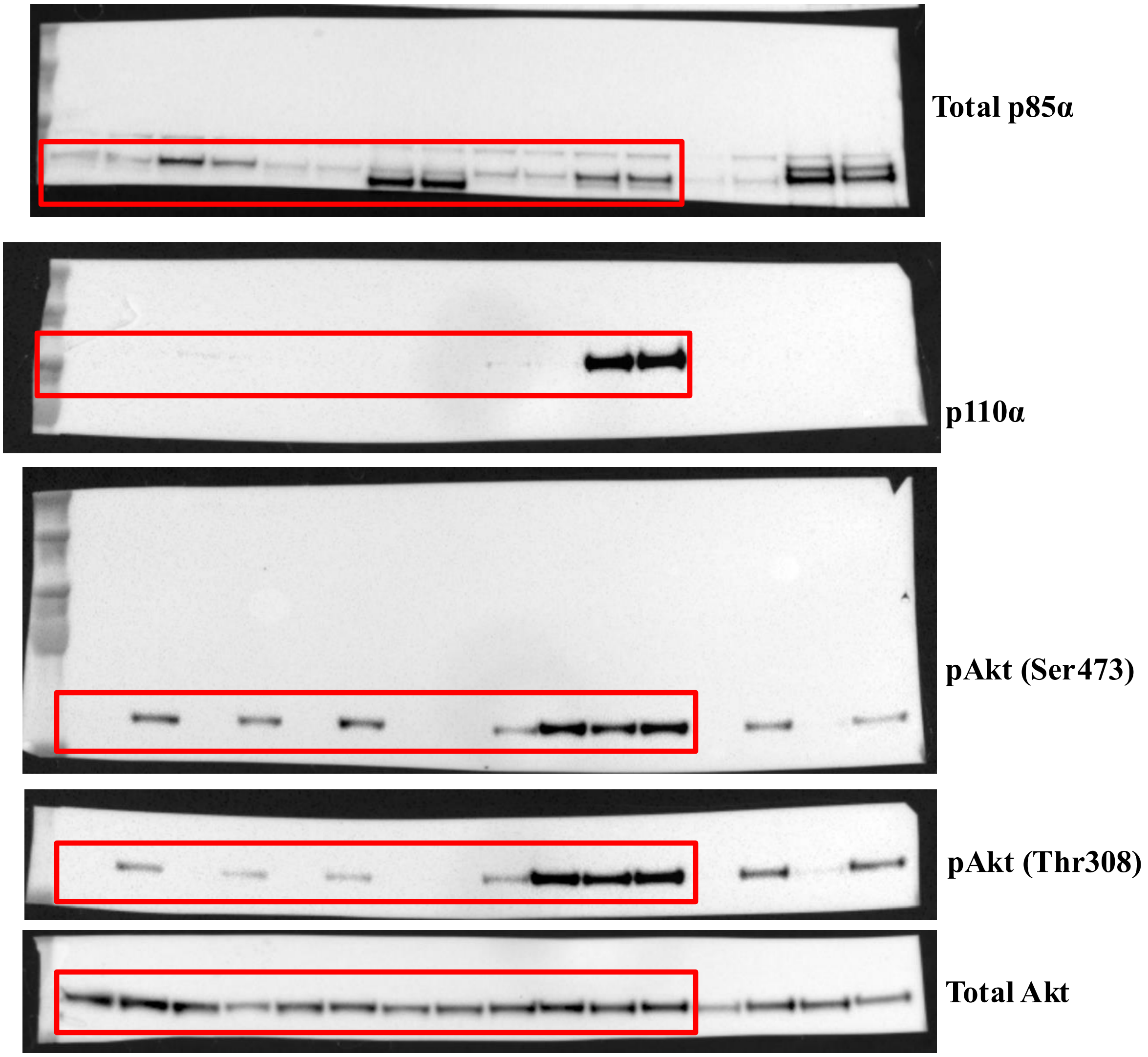

Supplement: Figure 2—source data 2. [file elife-94420-fig2-data2.pdf]
