# Supplementary material for: Paradoxical dominant negative activity of an immunodeficiency-associated activating PIK3R1 variant
Source: eLife. 2025 Jan 21;13:RP94420. doi: 10.7554/eLife.94420 (PMC11750134; doi:10.7554/eLife.94420)

Figure 2-figure supplement 3 – Images shown

(Doxycycline dose response: 0,0.02, 0.03, 0.045, 0.065, 0.1ug/mL)

Control

Total p85α

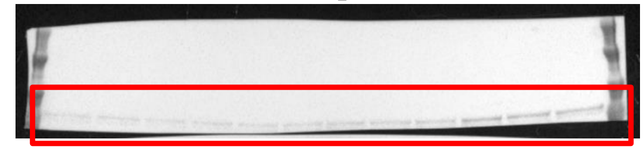

pAkt (Ser473)

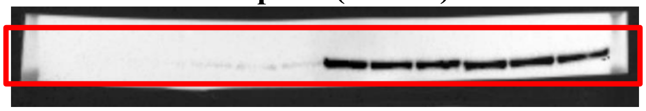

Total Akt

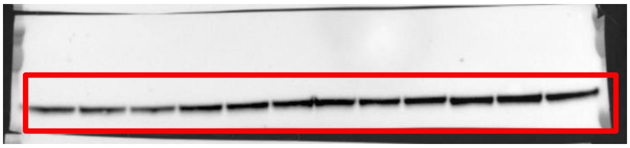

p85α ΔEx11

Total p85α

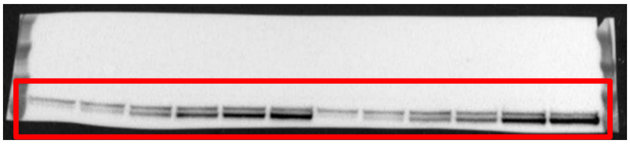

pAkt (Ser473)

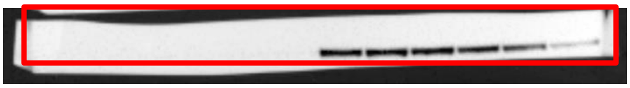

Total Akt

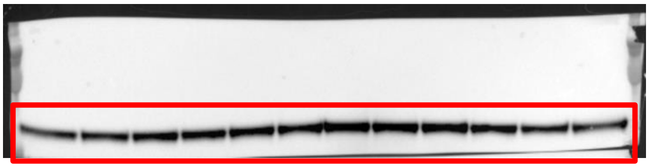

p85α WT

Total p85α

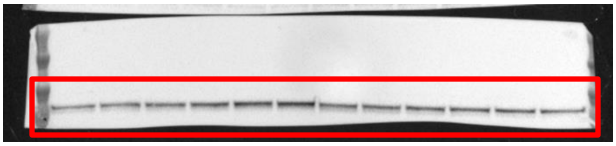

pAkt (Ser473)

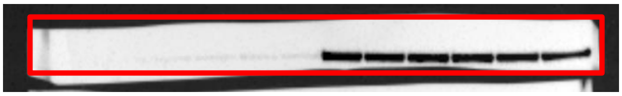

Total Akt

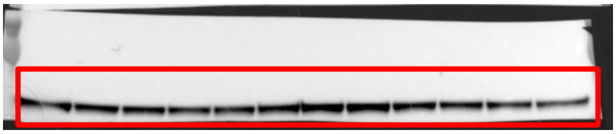

Figure 2-figure supplement 3 – Replicate but with wider doxycycline dose range (0,0.0001,0.001,0.01,0.1,1 ug/mL)

Control

Total p85α

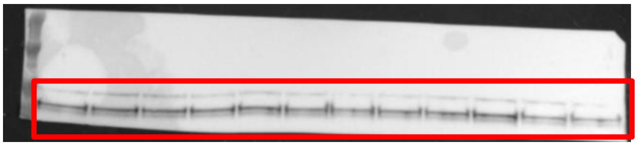

pAkt (Ser473)

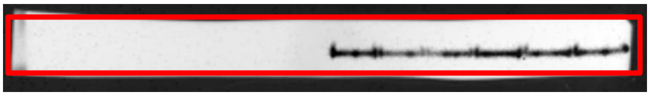

Total Akt

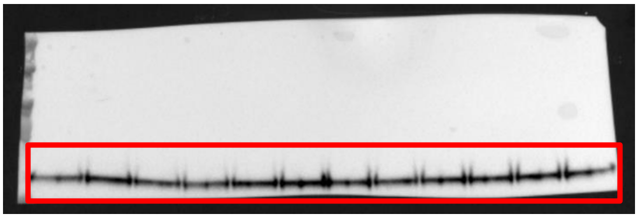

p85α ΔEx11

Total p85α

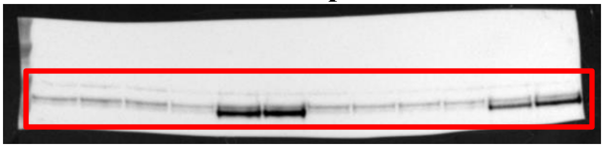

pAkt (Ser473)

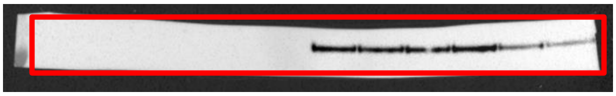

Total Akt

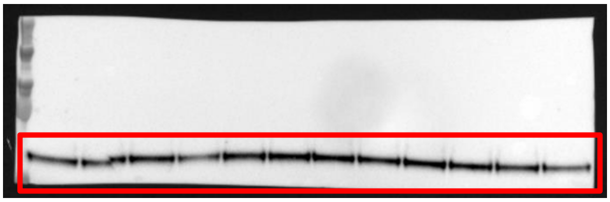

p85α WT

Total p85α

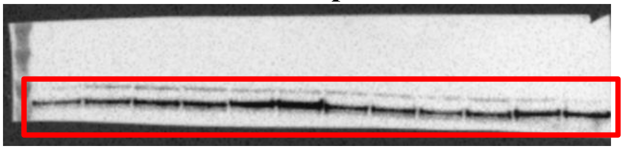

pAkt (Ser473)

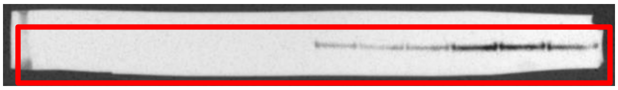

Total Akt

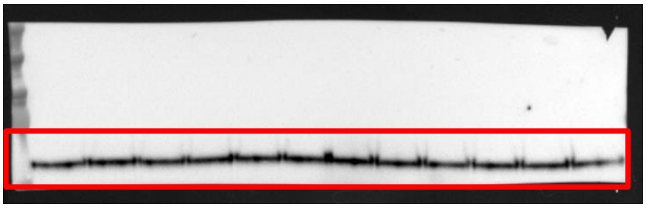

Supplement: Figure 2—figure supplement 3—source data 2. [file elife-94420-fig2-figsupp3-data2.pdf]
