# Supplementary material for: Paradoxical dominant negative activity of an immunodeficiency-associated activating PIK3R1 variant
Source: eLife. 2025 Jan 21;13:RP94420. doi: 10.7554/eLife.94420 (PMC11750134; doi:10.7554/eLife.94420)

Figure 4 – Images Shown

Total p85α IP:p110a

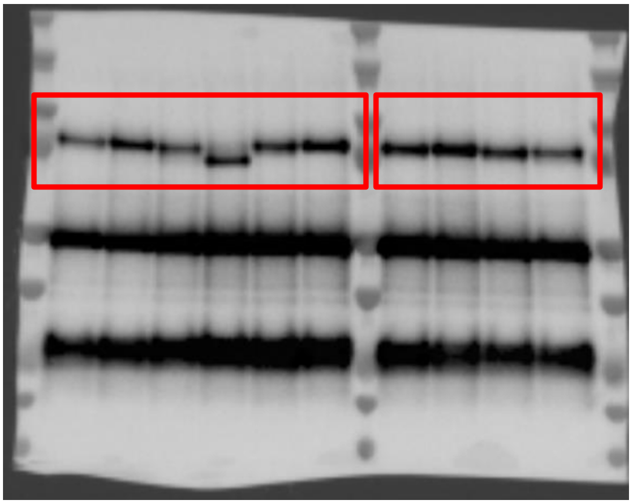

p110α

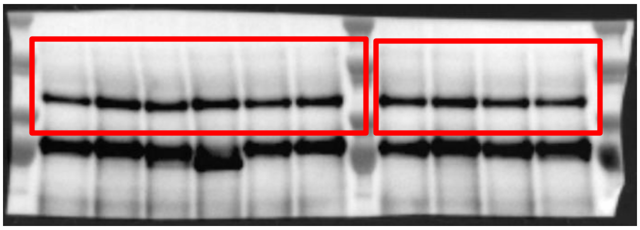

Total cell lysates

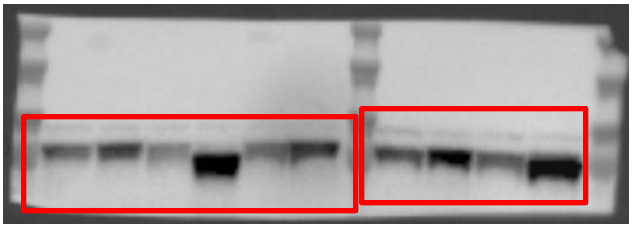

p110α

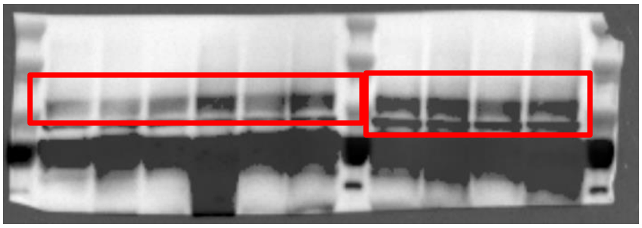

β-actin

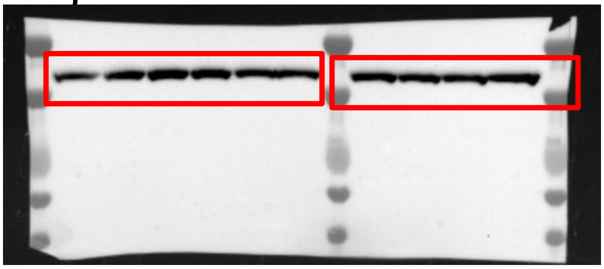

Supernatant

p85α

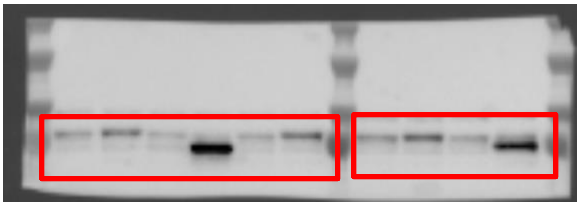

p110α

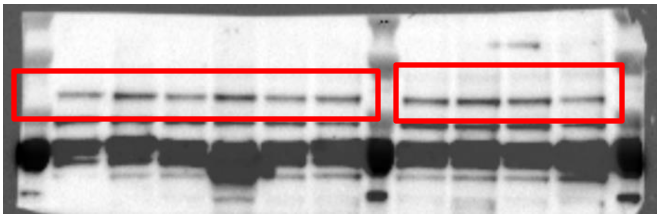

β-actin

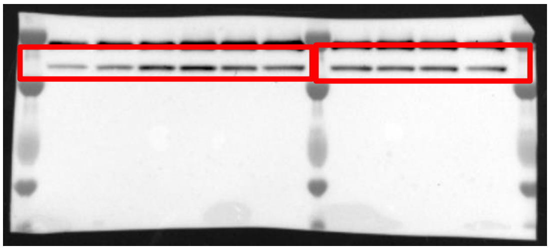

Figure 4 – Replicate 1

IP: p110α

p85α

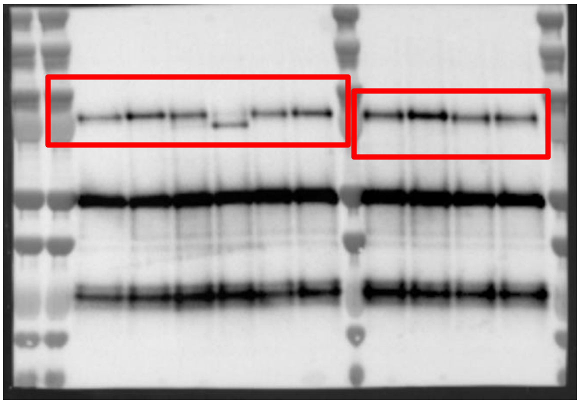

p110α

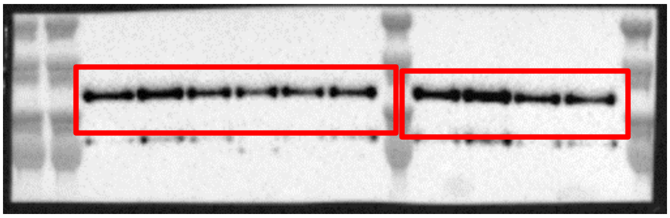

Total cell lysates

p85α

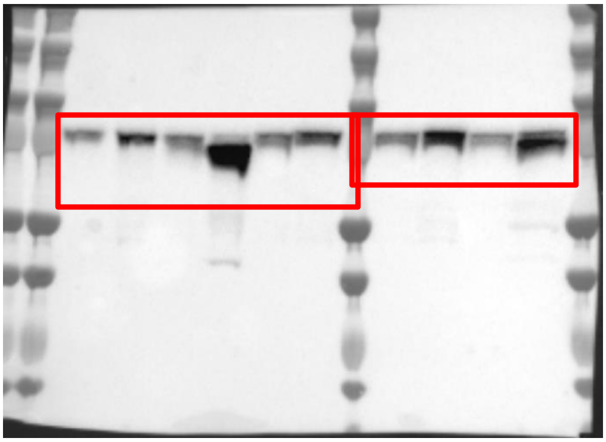

p110α

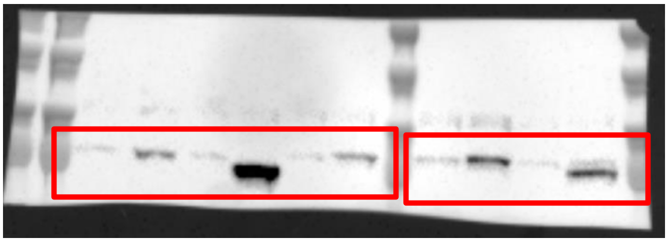

β-actin

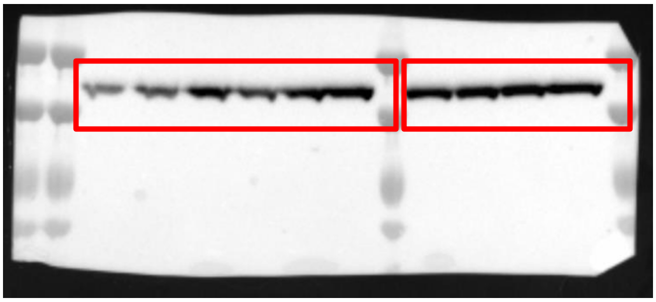

Supernatant

p85α

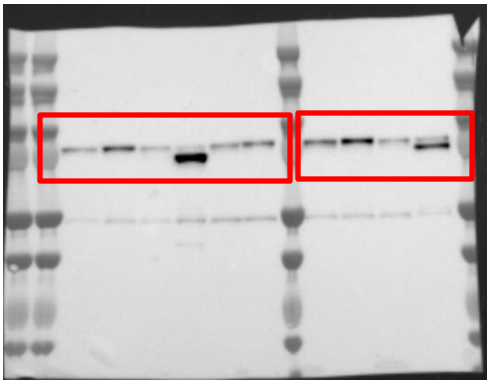

p110α

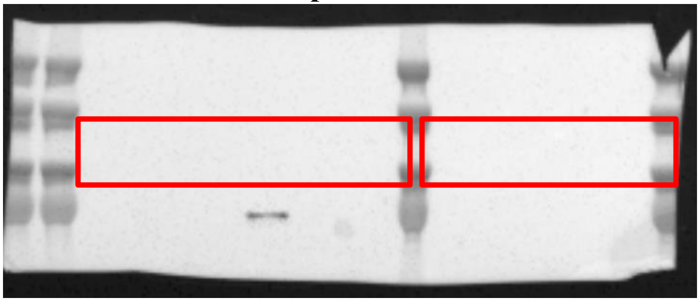

β-actin

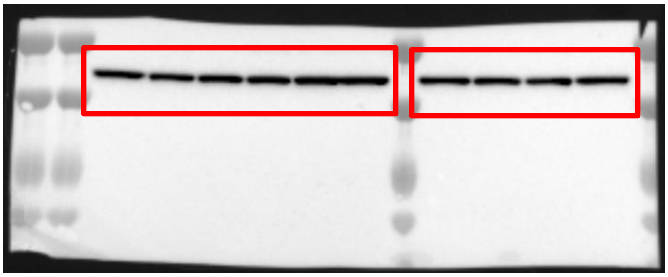

Figure 4 – Replicate 2

IP: p110α

p85α

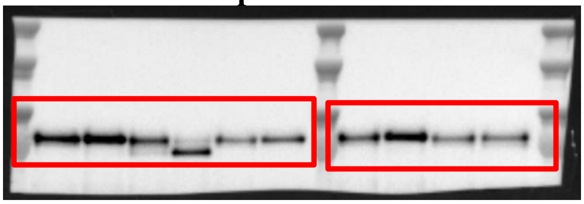

p110α

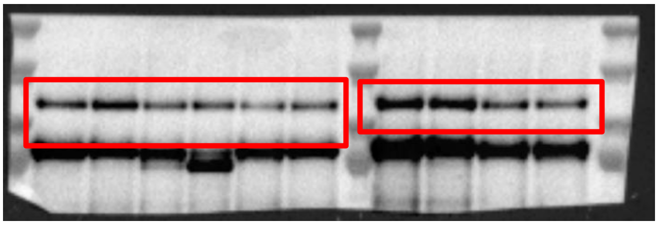

Total cell lysates

p85α

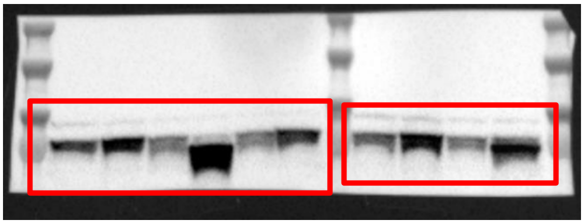

p110α

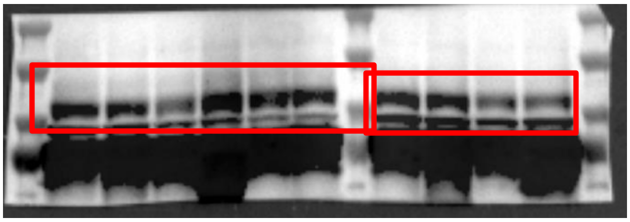

β-actin

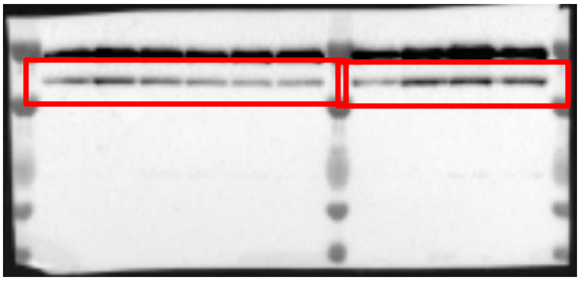

Supernatant

p85α

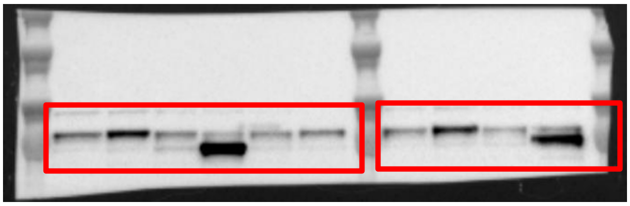

p110α

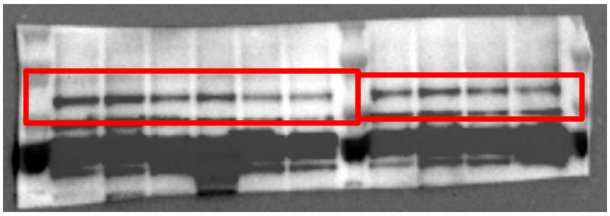

β-actin

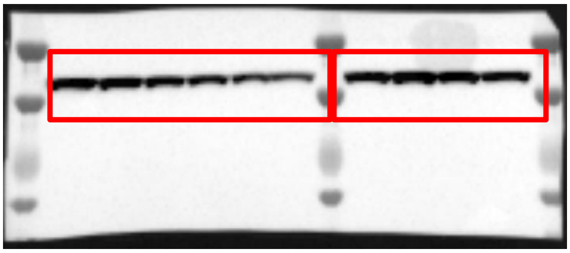

Supplement: Figure 4—source data 2. [file elife-94420-fig4-data2.pdf]
