# Supplementary figures and images for: Paradoxical dominant negative activity of an immunodeficiency-associated activating PIK3R1 variant (part 2 of 2)
Source: eLife. 2025 Jan 21;13:RP94420. doi: 10.7554/eLife.94420 (PMC11750134; doi:10.7554/eLife.94420)

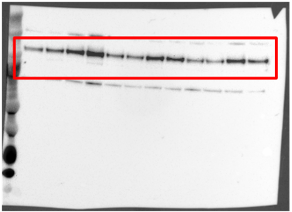

Supplement: Figure 5—source data 1. [file elife-94420-fig5-data1.zip › Figure_5_source_data_1/Fig5A_lysate_p110alpha_replicate_1_source_image_1.png]

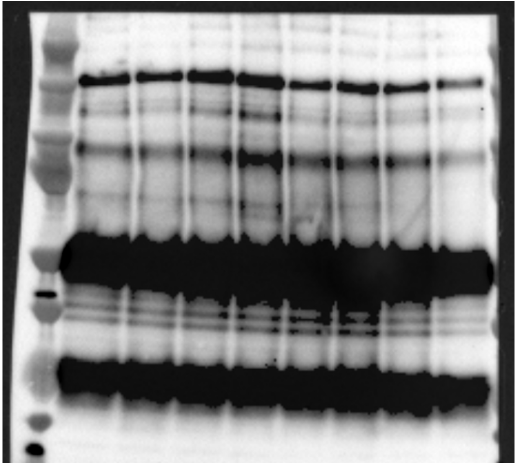

Supplement: Figure 5—source data 1. [file elife-94420-fig5-data1.zip › Figure_5_source_data_1/Fig5A_IP_IRS1_replicate_1_source_image_2.png]

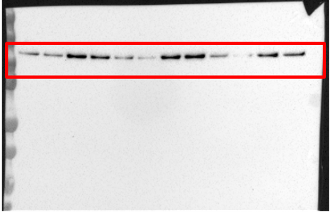

Supplement: Figure 5—source data 1. [file elife-94420-fig5-data1.zip › Figure_5_source_data_1/Fig5A_supernatant_p110alpha_replicate_2_source_image_1.png]

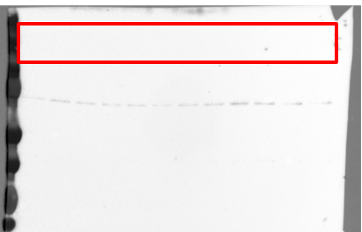

Supplement: Figure 5—source data 1. [file elife-94420-fig5-data1.zip › Figure_5_source_data_1/Fig5A_supernatant_IRS1_replicate_2_source_image_1.png]

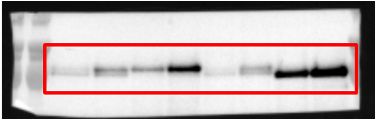

Supplement: Figure 5—source data 1. [file elife-94420-fig5-data1.zip › Figure_5_source_data_1/Fig5A_IP_p85alpha_source_image_2.png]

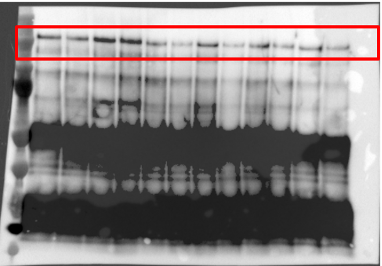

Supplement: Figure 5—source data 1. [file elife-94420-fig5-data1.zip › Figure_5_source_data_1/Fig5A_IP_IRS1_replicate_1_source_image_1..png]

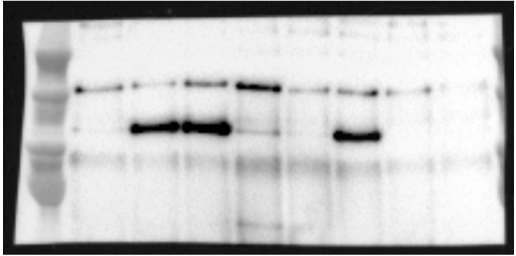

Supplement: Figure 5—source data 1. [file elife-94420-fig5-data1.zip › Figure_5_source_data_1/Fig5A_IP_p110alpha_replicate_1_source_image_2.png]

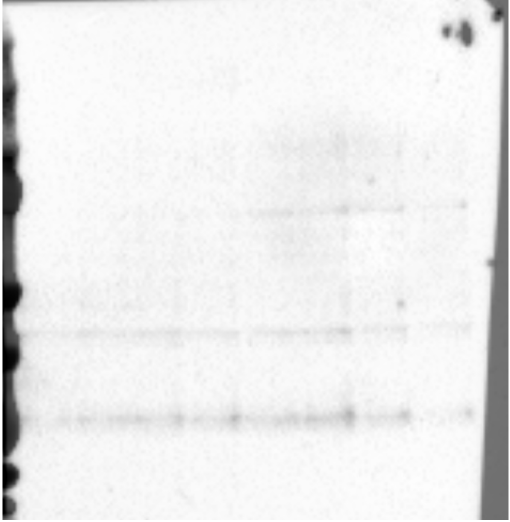

Supplement: Figure 5—source data 1. [file elife-94420-fig5-data1.zip › Figure_5_source_data_1/Fig5A_supernatant_IRS1_replicate_2_source_image_2.png]

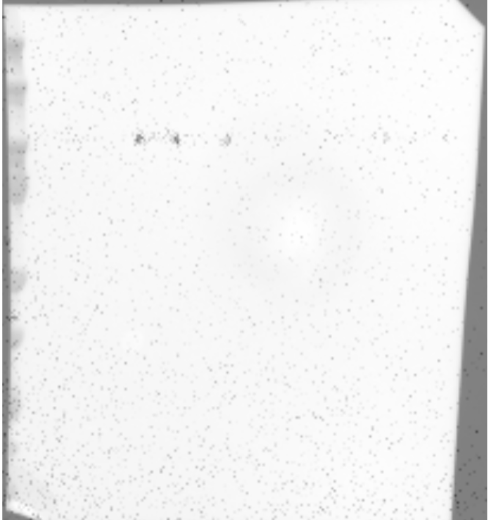

Supplement: Figure 5—source data 1. [file elife-94420-fig5-data1.zip › Figure_5_source_data_1/Fig5A_supernatant_p110alpha_replicate_2_source_image_2.png]

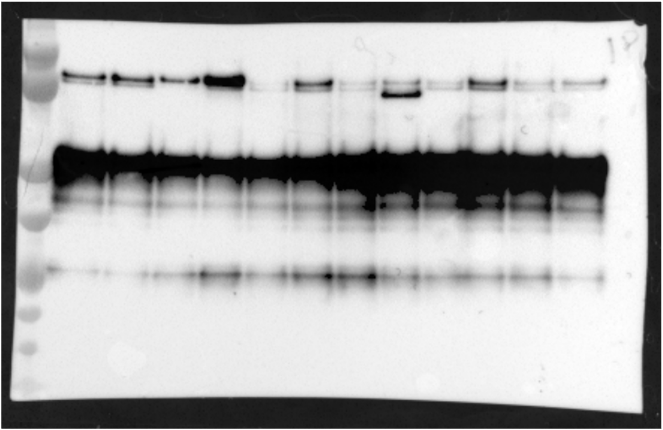

Supplement: Figure 5—source data 1. [file elife-94420-fig5-data1.zip › Figure_5_source_data_1/Fig5A_IP_p85alpha_source_image_1.png]

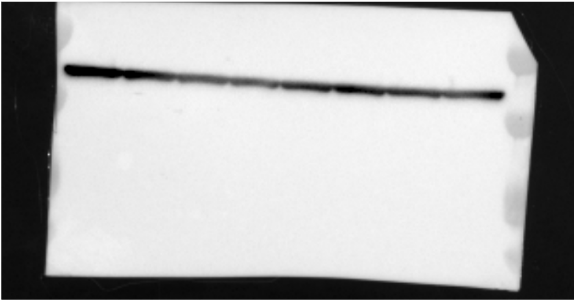

Supplement: Figure 5—source data 1. [file elife-94420-fig5-data1.zip › Figure_5_source_data_1/Fig5A_supernatant_beta_actin_source_image_2.png]

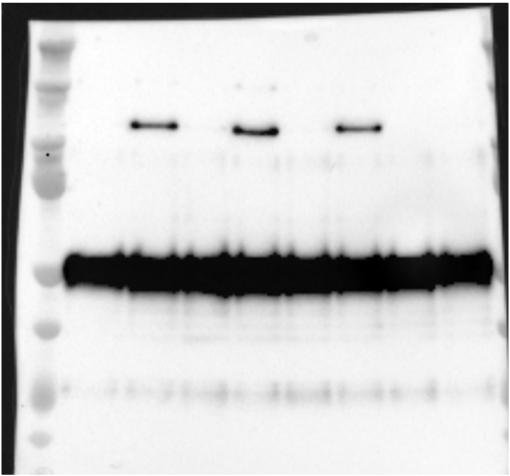

Supplement: Figure 5—source data 1. [file elife-94420-fig5-data1.zip › Figure_5_source_data_1/Fig5A_IP_p110alpha_replicate_2_source_image_2.png]

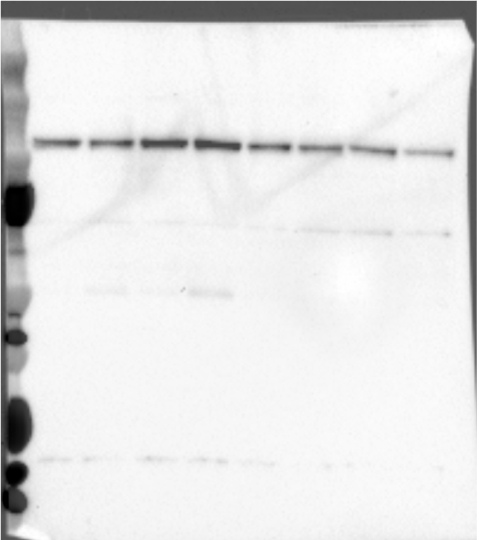

Supplement: Figure 5—source data 1. [file elife-94420-fig5-data1.zip › Figure_5_source_data_1/Fig5A_supernatant_p110alpha_replicate_1_source_image_2.png]

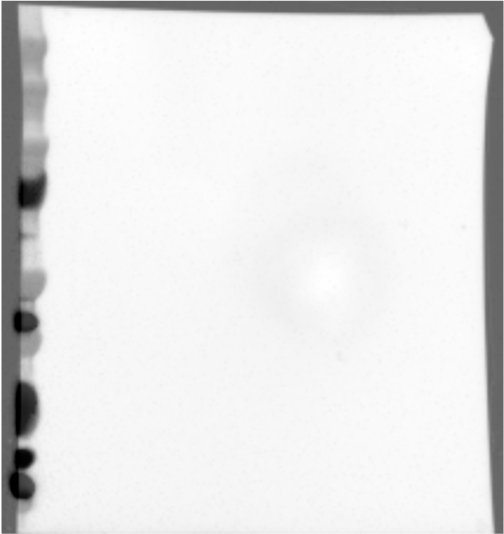

Supplement: Figure 5—source data 1. [file elife-94420-fig5-data1.zip › Figure_5_source_data_1/Fig5A_supernatant_IRS1_replicate_1_source_image_2.png]

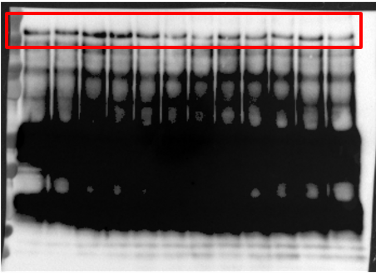

Supplement: Figure 5—source data 1. [file elife-94420-fig5-data1.zip › Figure_5_source_data_1/Fig5A_IP_IRS1_replicate_2_source_image_1.png]

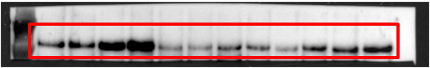

Supplement: Figure 5—source data 1. [file elife-94420-fig5-data1.zip › Figure_5_source_data_1/Fig5A_IP_IRS1_source_image_1.png]

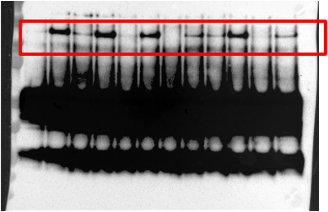

Supplement: Figure 5—source data 1. [file elife-94420-fig5-data1.zip › Figure_5_source_data_1/Fig5A_IP_p110alpha_replicate_2_source_image_1.png]

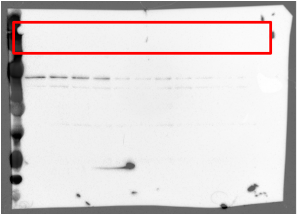

Supplement: Figure 5—source data 1. [file elife-94420-fig5-data1.zip › Figure_5_source_data_1/Fig5A_supernatant_IRS1_replicate_1_source_image_1.png]

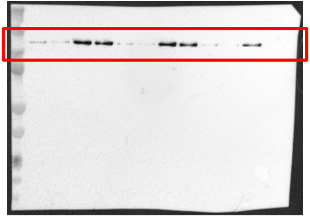

Supplement: Figure 5—source data 1. [file elife-94420-fig5-data1.zip › Figure_5_source_data_1/Fig5A_supernatant_p110alpha_replicate_1_source_image_1.png]

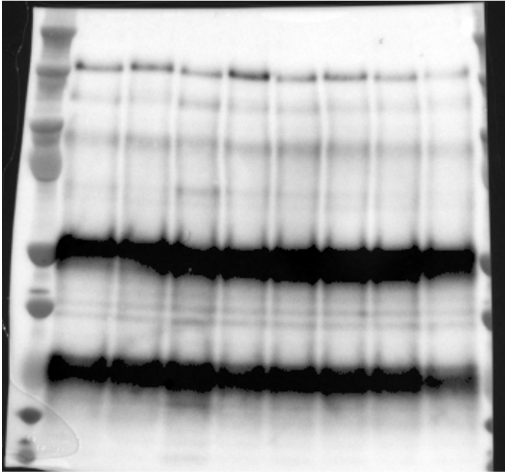

Supplement: Figure 5—source data 1. [file elife-94420-fig5-data1.zip › Figure_5_source_data_1/Fig5A_IP_IRS1_replicate_2_source_image_2.png]

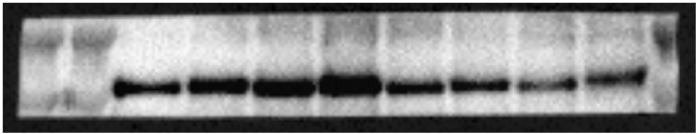

Supplement: Figure 5—source data 1. [file elife-94420-fig5-data1.zip › Figure_5_source_data_1/Fig5A_IP_IRS1_source_image_2.png]

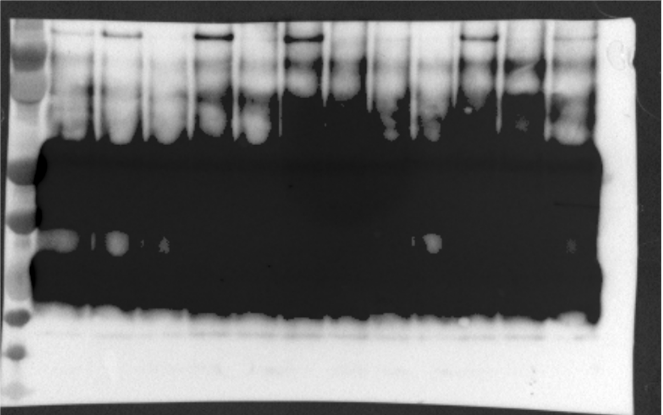

Supplement: Figure 5—source data 1. [file elife-94420-fig5-data1.zip › Figure_5_source_data_1/Fig5A_IP_p110alpha_source_image_1.png]

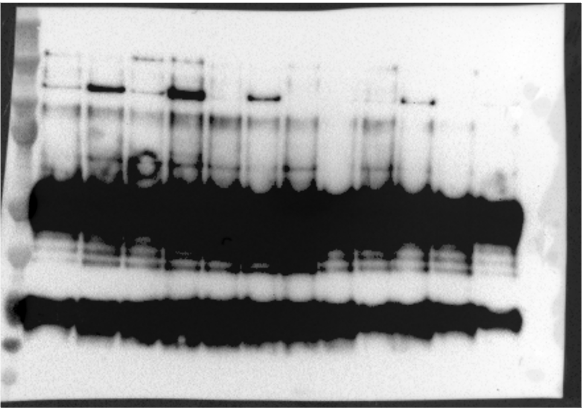

Supplement: Figure 5—source data 1. [file elife-94420-fig5-data1.zip › Figure_5_source_data_1/Fig5A_IP_p110alpha_replicate_1_source_image_1..png]

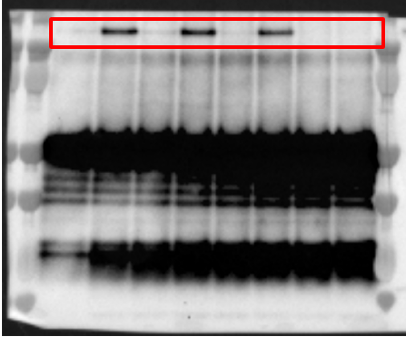

Supplement: Figure 5—source data 1. [file elife-94420-fig5-data1.zip › Figure_5_source_data_1/Fig5A_IP_p110alpha_source_image_2.png]

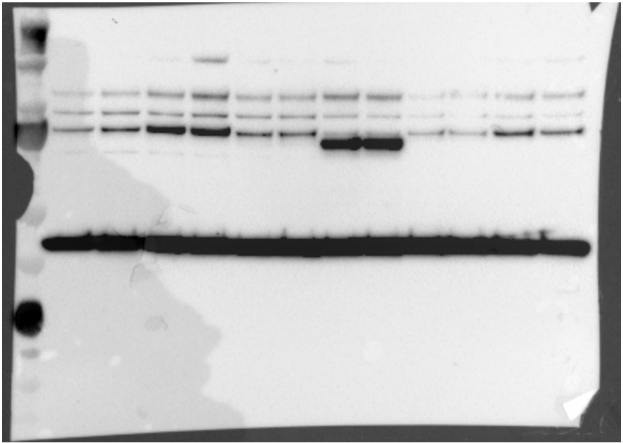

Supplement: Figure 5—source data 1. [file elife-94420-fig5-data1.zip › Figure_5_source_data_1/Fig5A_lysate_p85alpha_source_image_1.png]

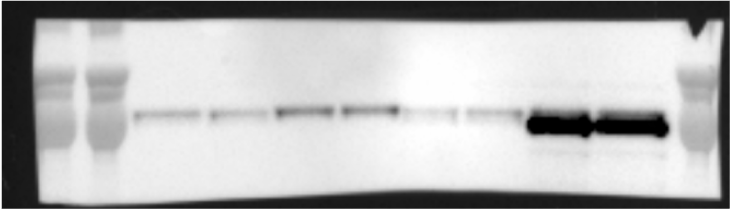

Supplement: Figure 5—source data 1. [file elife-94420-fig5-data1.zip › Figure_5_source_data_1/Fig5A_lysate_p85alpha_source_image_2.png]

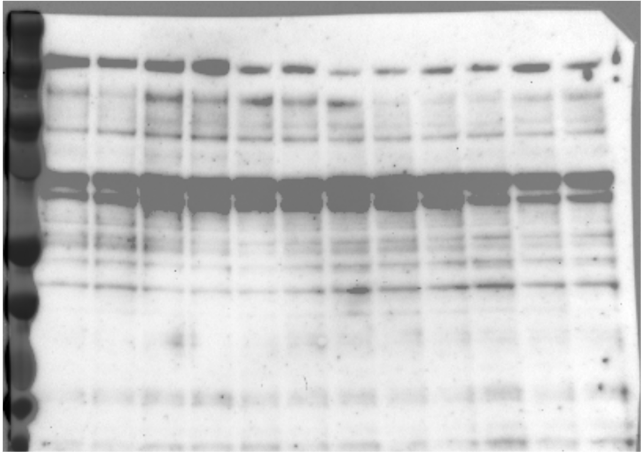

Supplement: Figure 5—source data 1. [file elife-94420-fig5-data1.zip › Figure_5_source_data_1/Fig5A_lysate_IRS1_replicate_2_source_image_1.png]

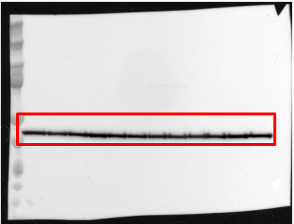

Supplement: Figure 5—source data 1. [file elife-94420-fig5-data1.zip › Figure_5_source_data_1/Fig5A_lysate_beta_actin_replicate_1_source_image_1.png]

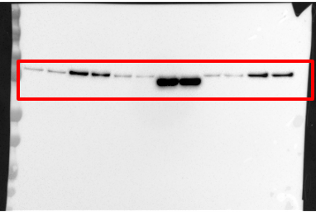

Supplement: Figure 5—source data 1. [file elife-94420-fig5-data1.zip › Figure_5_source_data_1/Fig5A_supernatant_p85alpha_replicate_2_source_image_1.png]

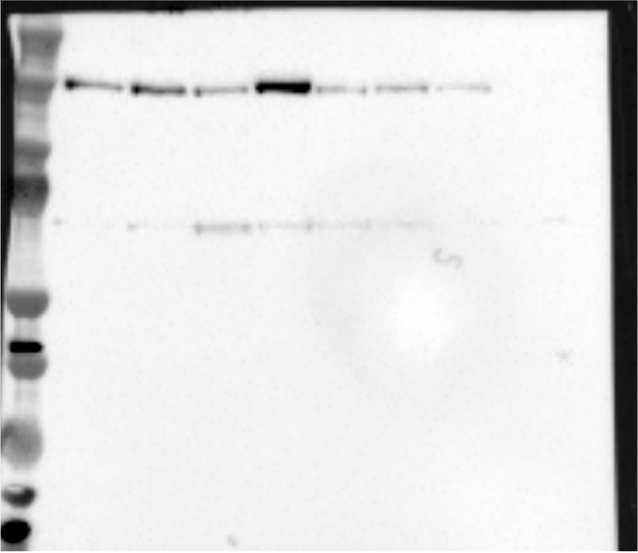

Supplement: Figure 5—source data 1. [file elife-94420-fig5-data1.zip › Figure_5_source_data_1/Fig5A_lysate_IRS1_replicate_2_source_image_2.png]

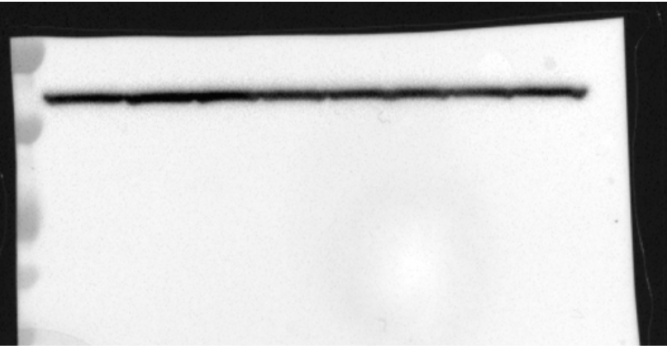

Supplement: Figure 5—source data 1. [file elife-94420-fig5-data1.zip › Figure_5_source_data_1/Fig5A_lysate_beta_actin_replicate_1_source_image_2.png]

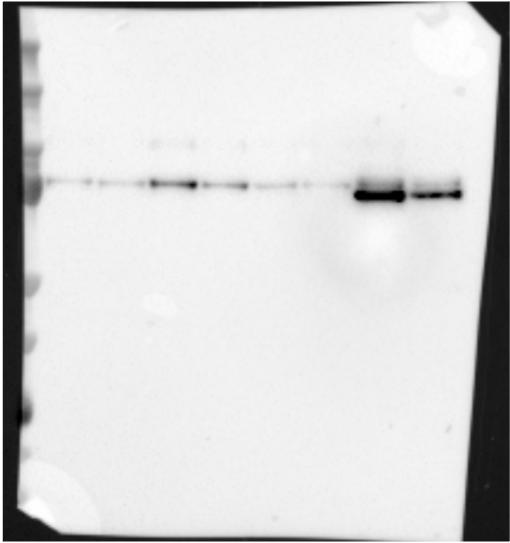

Supplement: Figure 5—source data 1. [file elife-94420-fig5-data1.zip › Figure_5_source_data_1/Fig5A_supernatant_p85alpha_replicate_2_source_image_2.png]

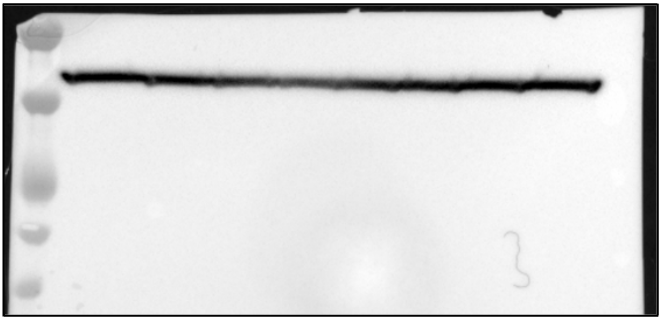

Supplement: Figure 5—source data 1. [file elife-94420-fig5-data1.zip › Figure_5_source_data_1/Fig5A_lysate_beta_actin_replicate_2_source_image_2.png]

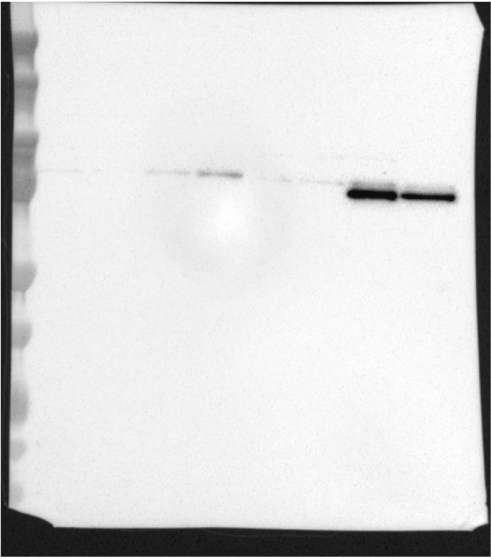

Supplement: Figure 5—source data 1. [file elife-94420-fig5-data1.zip › Figure_5_source_data_1/Fig5A_supernatant_p85alpha_replicate_1_source_image_2.png]

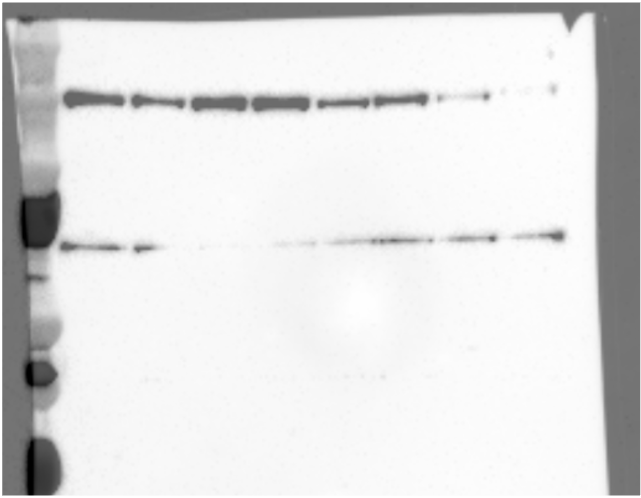

Supplement: Figure 5—source data 1. [file elife-94420-fig5-data1.zip › Figure_5_source_data_1/Fig5A_lysate_IRS1_replicate_1_source_image_2.png]

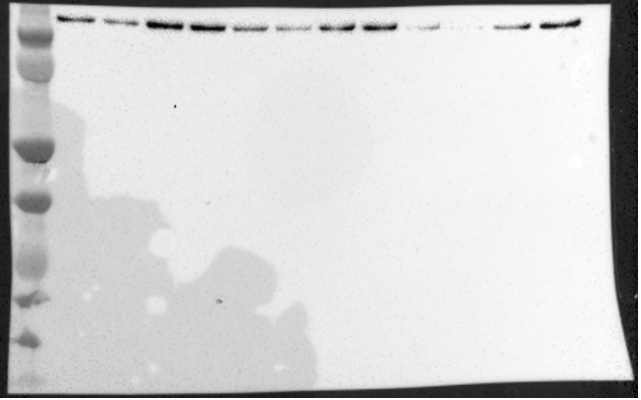

Supplement: Figure 5—source data 1. [file elife-94420-fig5-data1.zip › Figure_5_source_data_1/Fig5A_supernatant_p110alpha_source_image_1.png]

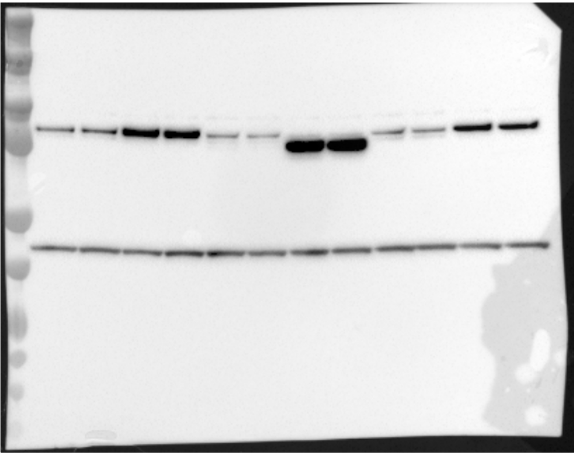

Supplement: Figure 5—source data 1. [file elife-94420-fig5-data1.zip › Figure_5_source_data_1/Fig5A_lysate_beta_actin_replicate_2_source_image_1.png]

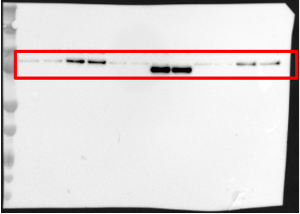

Supplement: Figure 5—source data 1. [file elife-94420-fig5-data1.zip › Figure_5_source_data_1/Fig5A_supernatant_p85alpha_replicate_1_source_image_1.png]

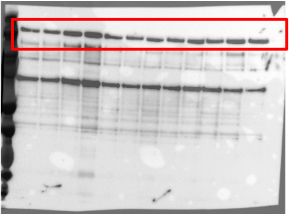

Supplement: Figure 5—source data 1. [file elife-94420-fig5-data1.zip › Figure_5_source_data_1/Fig5A_lysate_IRS1_replicate_1_source_image_1.png]

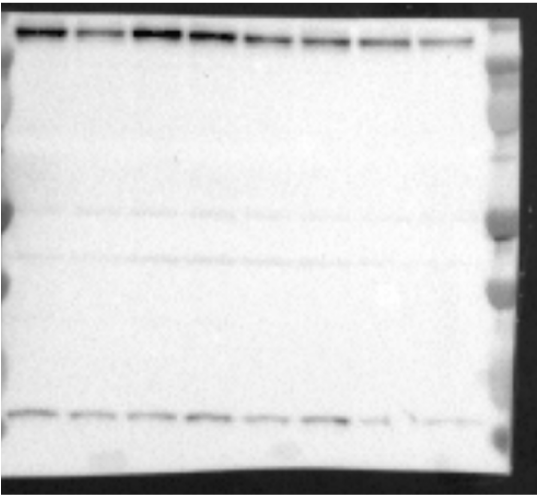

Supplement: Figure 5—source data 1. [file elife-94420-fig5-data1.zip › Figure_5_source_data_1/Fig5A_supernatant_p110alpha_source_image_2.png]

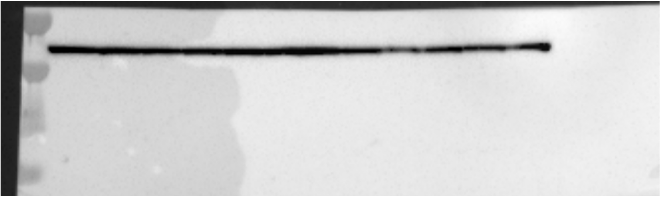

Supplement: Figure 6—source data 1. [file elife-94420-fig6-data1.zip › Figure_6_source_data_1/Fig6A_supernatant_beta_actin_source_image_1.png]

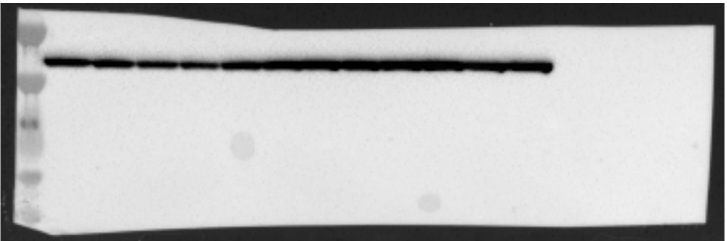

Supplement: Figure 6—source data 1. [file elife-94420-fig6-data1.zip › Figure_6_source_data_1/Fig6A_lysate_beta_actin_source_image_1.png]

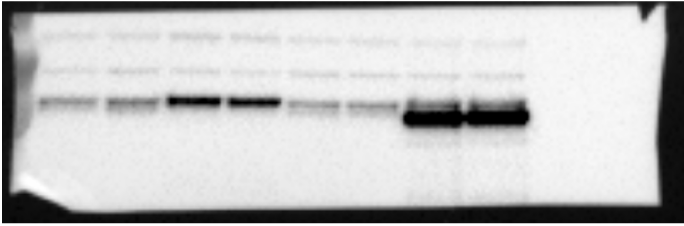

Supplement: Figure 6—source data 1. [file elife-94420-fig6-data1.zip › Figure_6_source_data_1/Fig6A_supernatant_p85alpha_replicate_2_source_image_1.png]

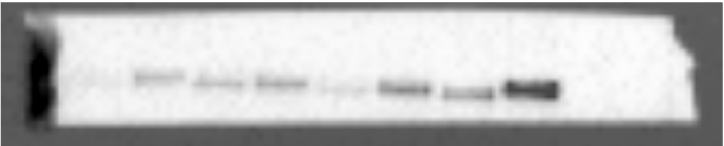

Supplement: Figure 6—source data 1. [file elife-94420-fig6-data1.zip › Figure_6_source_data_1/Fig6A_supernatant_IRS2_replicate_2_source_image_1.png]

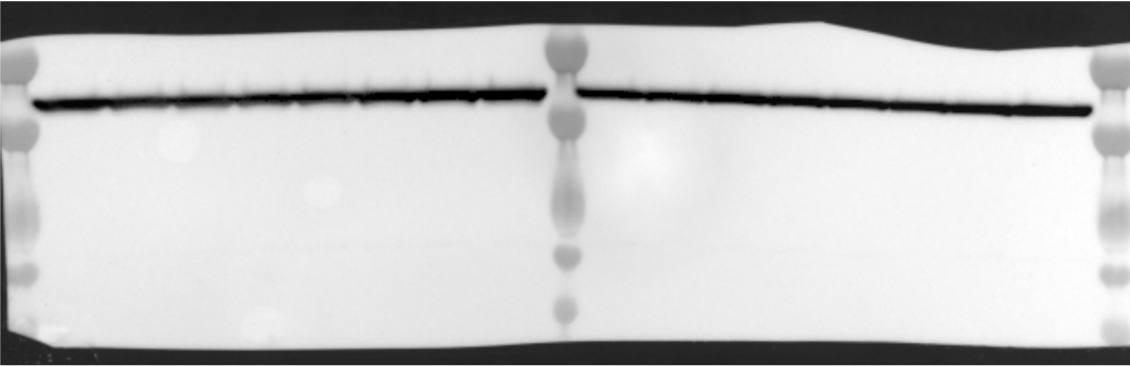

Supplement: Figure 6—source data 1. [file elife-94420-fig6-data1.zip › Figure_6_source_data_1/Fig6A_lysate&supernatant_beta_actin_source_image_2.png]

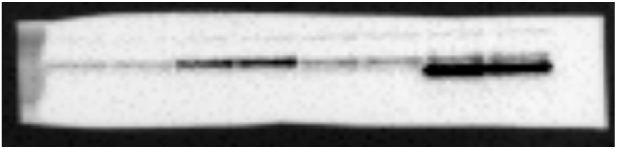

Supplement: Figure 6—source data 1. [file elife-94420-fig6-data1.zip › Figure_6_source_data_1/Fig6A_supernatant_p85alpha_replicate_1_source_image_2.png]

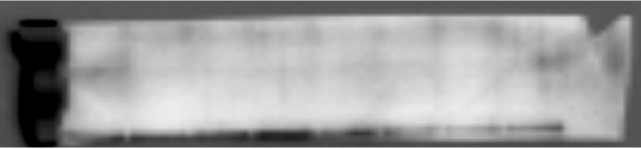

Supplement: Figure 6—source data 1. [file elife-94420-fig6-data1.zip › Figure_6_source_data_1/Fig6A_supernatant_IRS2_replicate_1_source_image_2.png]

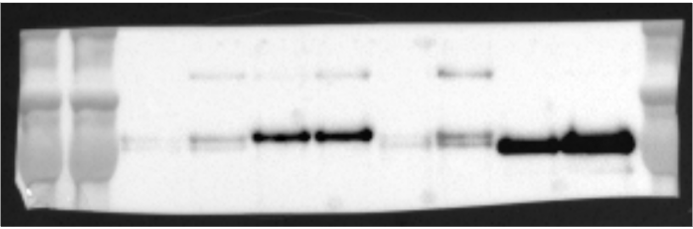

Supplement: Figure 6—source data 1. [file elife-94420-fig6-data1.zip › Figure_6_source_data_1/Fig6A_IP_p85alpha_source_image_2.png]

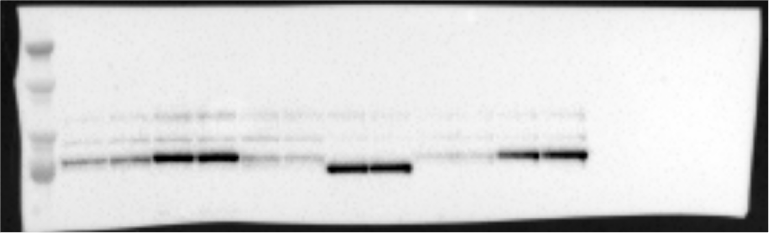

Supplement: Figure 6—source data 1. [file elife-94420-fig6-data1.zip › Figure_6_source_data_1/Fig6A_supernatant_p85alpha_replicate_1_source_image_1.png]

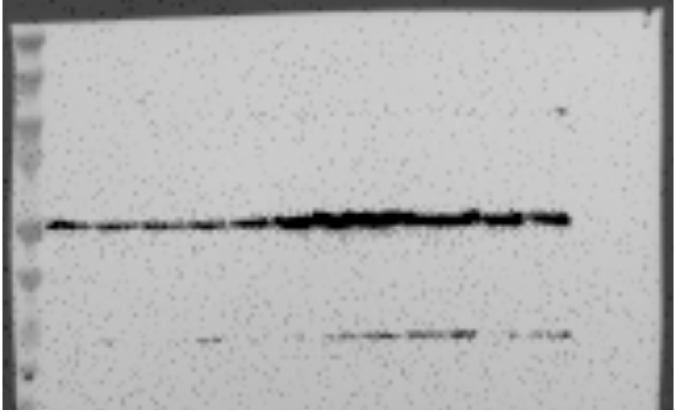

Supplement: Figure 6—source data 1. [file elife-94420-fig6-data1.zip › Figure_6_source_data_1/Fig6A_supernatant_IRS2_replicate_1_source_image_1.png]

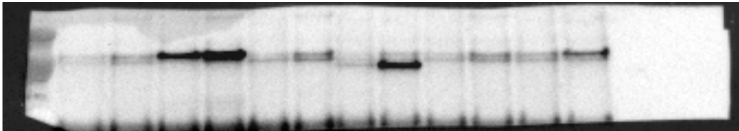

Supplement: Figure 6—source data 1. [file elife-94420-fig6-data1.zip › Figure_6_source_data_1/Fig6A_IP_p85alpha_source_image_1.png]

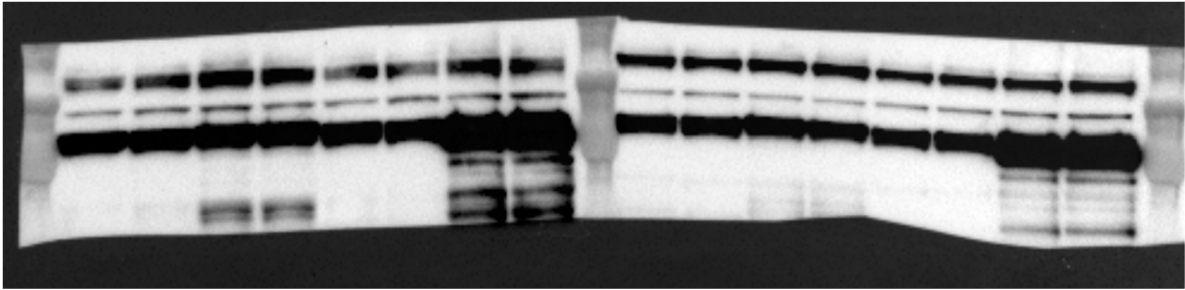

Supplement: Figure 6—source data 1. [file elife-94420-fig6-data1.zip › Figure_6_source_data_1/Fig6A_lysate&supernatant_p110alpha_source_image_2.png]

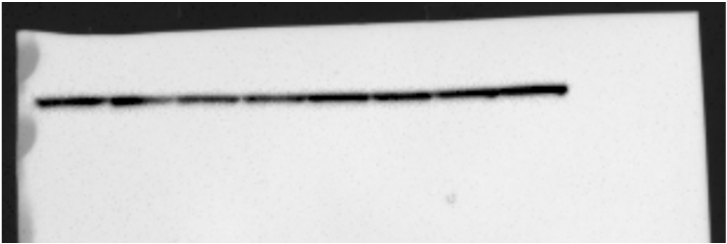

Supplement: Figure 6—source data 1. [file elife-94420-fig6-data1.zip › Figure_6_source_data_1/Fig6A_supernatant_beta_actin_replicate_2_source_image_1.png]

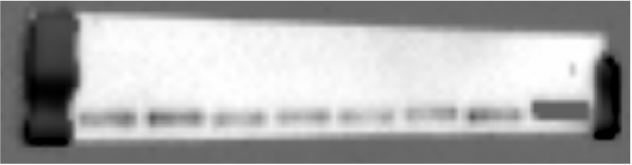

Supplement: Figure 6—source data 1. [file elife-94420-fig6-data1.zip › Figure_6_source_data_1/Fig6A_lysate_IRS2_source_image_2.png]

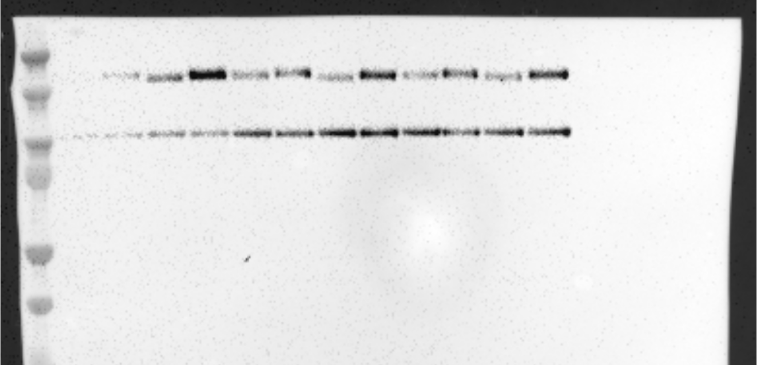

Supplement: Figure 6—source data 1. [file elife-94420-fig6-data1.zip › Figure_6_source_data_1/Fig6A_lysate_IRS2_replicate_1_source_image_1.png]

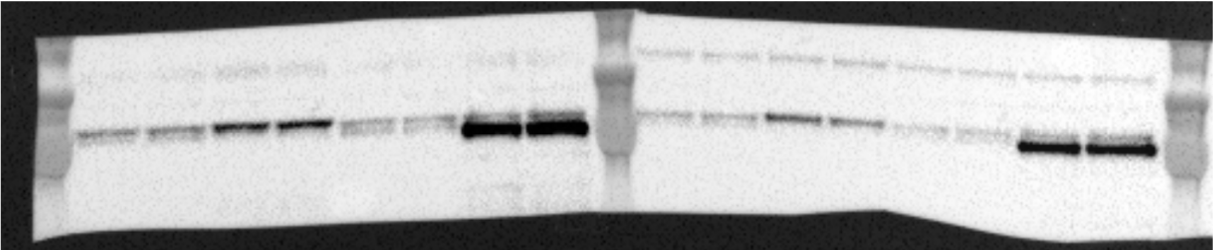

Supplement: Figure 6—source data 1. [file elife-94420-fig6-data1.zip › Figure_6_source_data_1/Fig6A_lysate&supernatant_p85alpha_source_image_2.png]

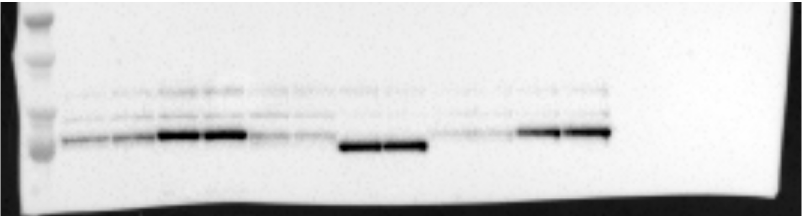

Supplement: Figure 6—source data 1. [file elife-94420-fig6-data1.zip › Figure_6_source_data_1/Fig6A_lysate_p85alpha_replicate_1_source_image_1.png]

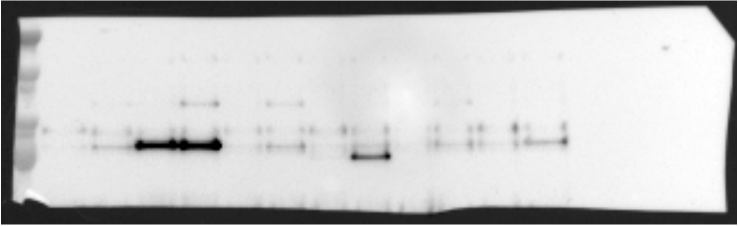

Supplement: Figure 6—source data 1. [file elife-94420-fig6-data1.zip › Figure_6_source_data_1/Fig6A_IP_p85alpha_replicate_1_source_image_1.png]

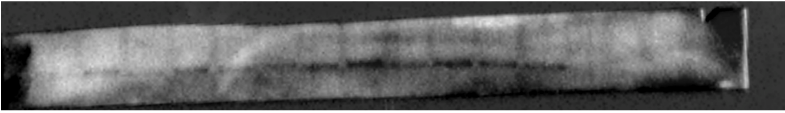

Supplement: Figure 6—source data 1. [file elife-94420-fig6-data1.zip › Figure_6_source_data_1/Fig6A_lysate_IRS2_source_image_1.png]

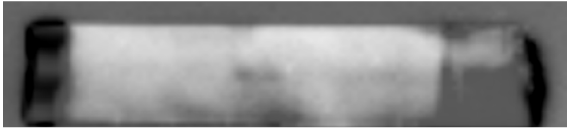

Supplement: Figure 6—source data 1. [file elife-94420-fig6-data1.zip › Figure_6_source_data_1/Fig6A_lysate_IRS2_replicate_1_source_image_2.png]

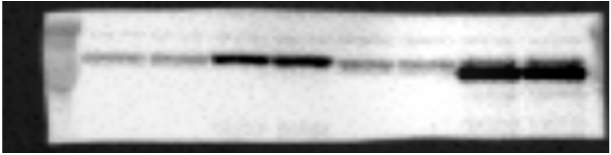

Supplement: Figure 6—source data 1. [file elife-94420-fig6-data1.zip › Figure_6_source_data_1/Fig6A_lysate_p85alpha_replicate_1_source_image_2.png]

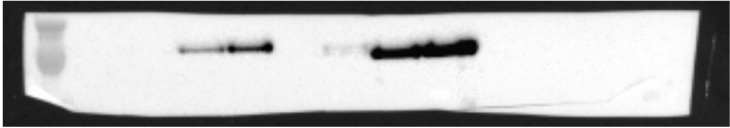

Supplement: Figure 6—source data 1. [file elife-94420-fig6-data1.zip › Figure_6_source_data_1/Fig6A_IP_p85alpha_replicate_1_source_image_2.png]

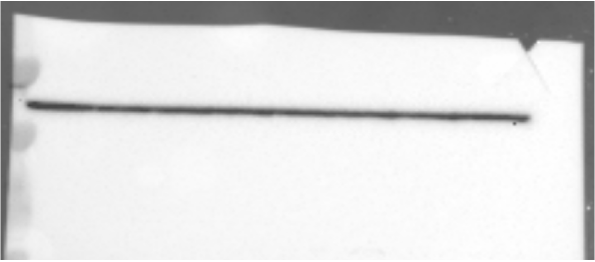

Supplement: Figure 6—source data 1. [file elife-94420-fig6-data1.zip › Figure_6_source_data_1/Fig6A_supernatant_beta_actin_replicate_1_source_image_2.png]

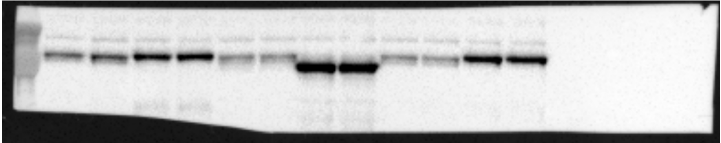

Supplement: Figure 6—source data 1. [file elife-94420-fig6-data1.zip › Figure_6_source_data_1/Fig6A_supernatant_p85alpha_source_image_1.png]

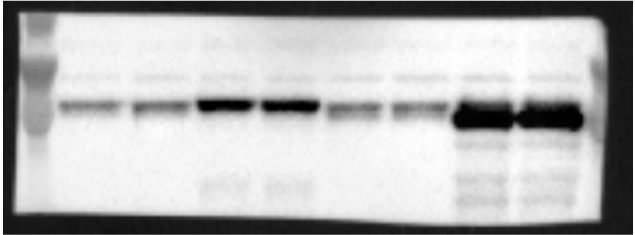

Supplement: Figure 6—source data 1. [file elife-94420-fig6-data1.zip › Figure_6_source_data_1/Fig6A_lysate_p85alpha_replicate_2_source_image_1.png]

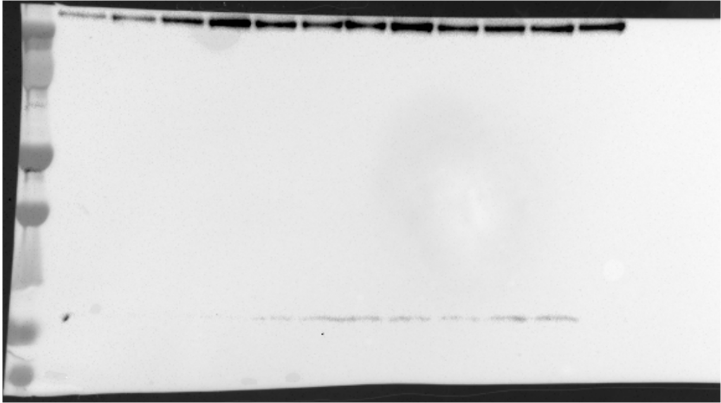

Supplement: Figure 6—source data 1. [file elife-94420-fig6-data1.zip › Figure_6_source_data_1/Fig6A_supernatant_p110alpha_source_image_1.png]

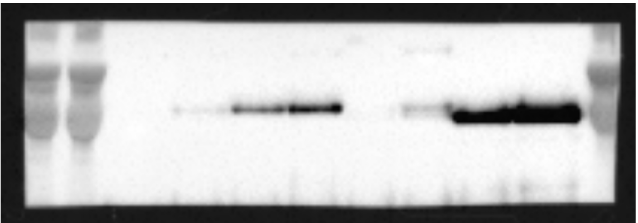

Supplement: Figure 6—source data 1. [file elife-94420-fig6-data1.zip › Figure_6_source_data_1/Fig6A_IP_p85alpha_replicate_2_source_image_1.png]

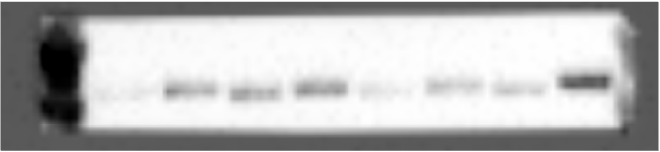

Supplement: Figure 6—source data 1. [file elife-94420-fig6-data1.zip › Figure_6_source_data_1/Fig6A_lysate_IRS2_replicate_2_source_image_1.png]

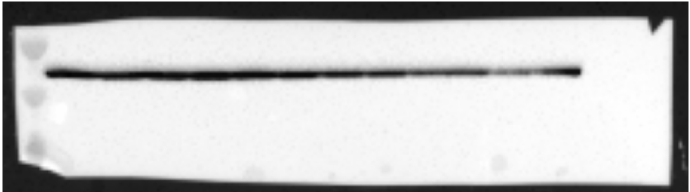

Supplement: Figure 6—source data 1. [file elife-94420-fig6-data1.zip › Figure_6_source_data_1/Fig6A_supernatant_beta_actin_replicate_1_source_image_1.png]

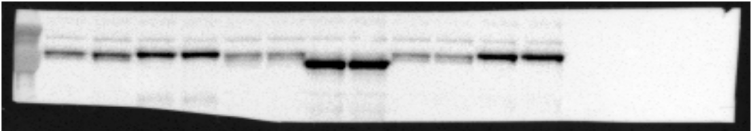

Supplement: Figure 6—source data 1. [file elife-94420-fig6-data1.zip › Figure_6_source_data_1/Fig6A_lysate_p85alpha_source_image_1.png]

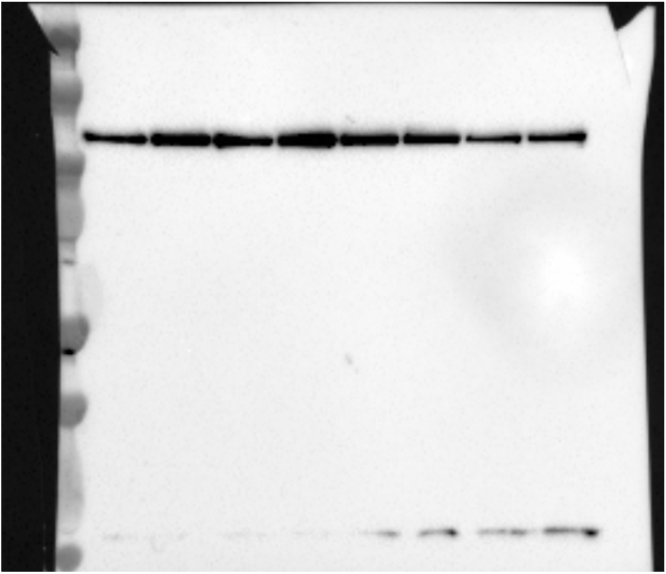

Supplement: Figure 6—source data 1. [file elife-94420-fig6-data1.zip › Figure_6_source_data_1/Fig6A_supernatant_p110alpha_replicate_1_source_image_2.png]

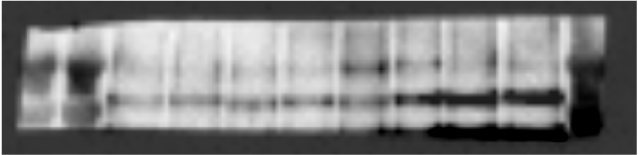

Supplement: Figure 6—source data 1. [file elife-94420-fig6-data1.zip › Figure_6_source_data_1/Fig6A_IP_IRS2_replicate_2_source_image_1.png]

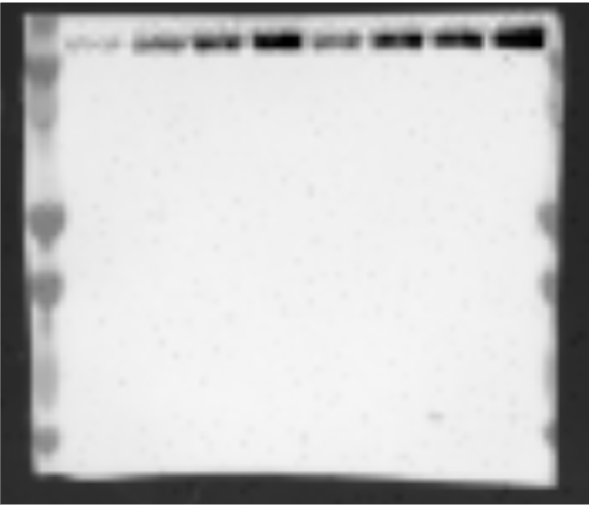

Supplement: Figure 6—source data 1. [file elife-94420-fig6-data1.zip › Figure_6_source_data_1/Fig6A_lysate_p110alpha_replicate_2_source_image_1.png]

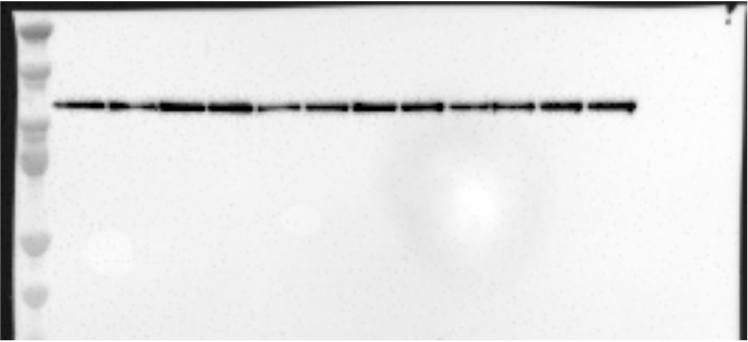

Supplement: Figure 6—source data 1. [file elife-94420-fig6-data1.zip › Figure_6_source_data_1/Fig6A_supernatant_p110alpha_replicate_1_source_image_1.png]

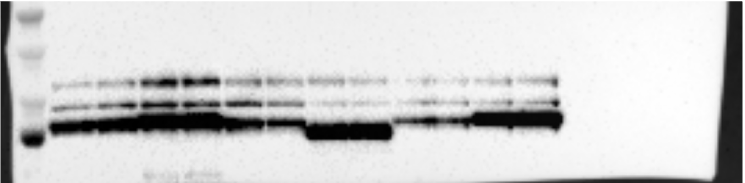

Supplement: Figure 6—source data 1. [file elife-94420-fig6-data1.zip › Figure_6_source_data_1/Fig6A_lysate_p110alpha_replicate_1_source_image_1.png]

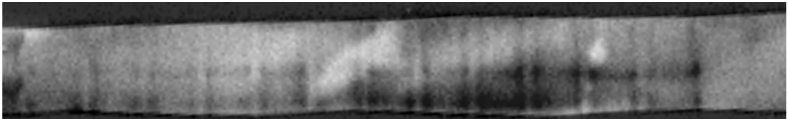

Supplement: Figure 6—source data 1. [file elife-94420-fig6-data1.zip › Figure_6_source_data_1/Fig6A_IP_IRS2_source_image_1.png]

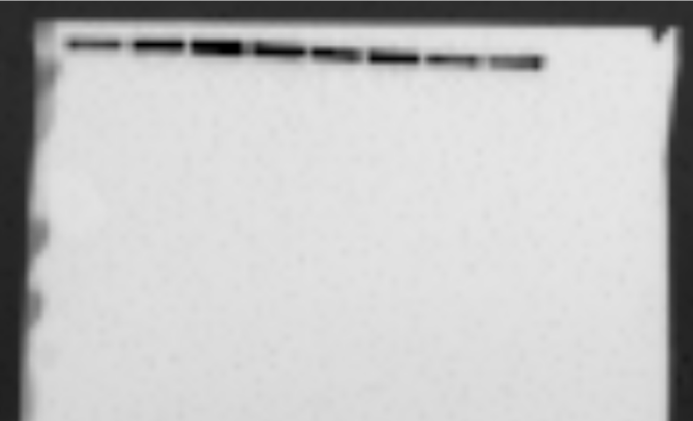

Supplement: Figure 6—source data 1. [file elife-94420-fig6-data1.zip › Figure_6_source_data_1/Fig6A_supernatant_p110alpha_replicate_2_source_image_1.png]

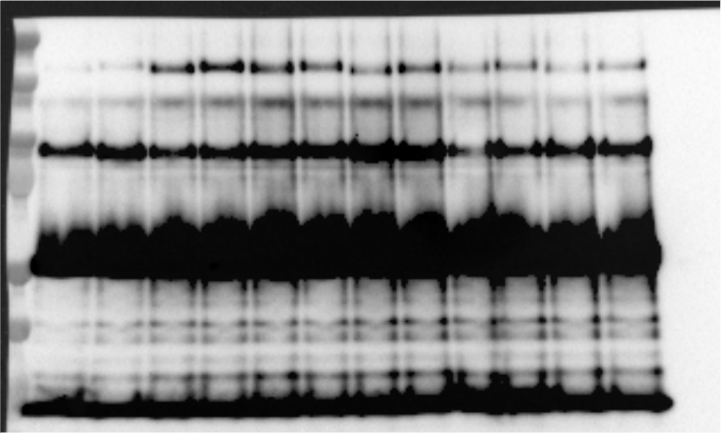

Supplement: Figure 6—source data 1. [file elife-94420-fig6-data1.zip › Figure_6_source_data_1/Fig6A_IP_IRS2_replicate_1_source_image_1.png]

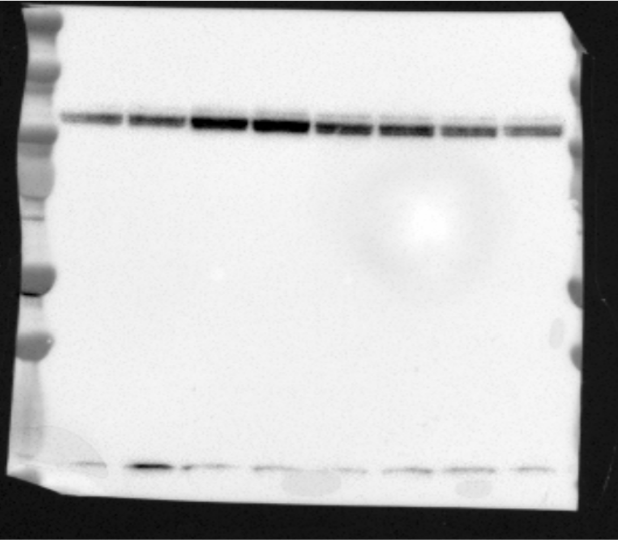

Supplement: Figure 6—source data 1. [file elife-94420-fig6-data1.zip › Figure_6_source_data_1/Fig6A_lysate_p110alpha_replicate_1_source_image_2.png]

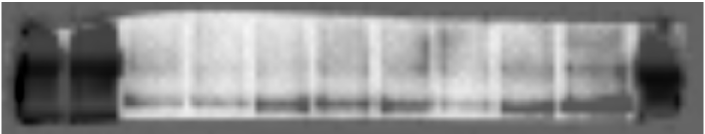

Supplement: Figure 6—source data 1. [file elife-94420-fig6-data1.zip › Figure_6_source_data_1/Fig6A_IP_IRS2_source_image_2.png]

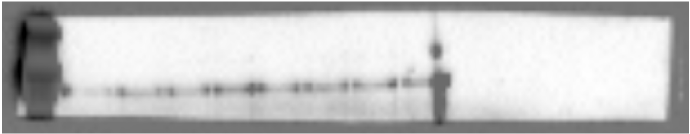

Supplement: Figure 6—source data 1. [file elife-94420-fig6-data1.zip › Figure_6_source_data_1/Fig6A_IP_IRS2_replicate_1_source_image_2.png]

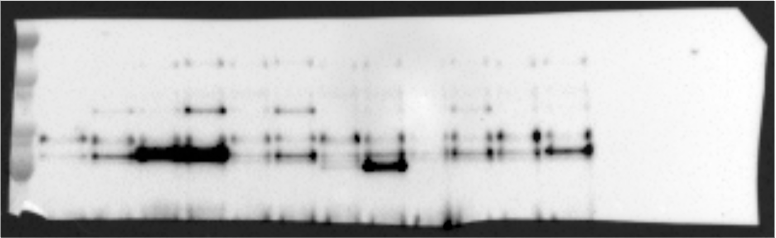

Supplement: Figure 6—source data 1. [file elife-94420-fig6-data1.zip › Figure_6_source_data_1/Fig6A_IP_p110alpha_replicate_1_source_image_1.png]

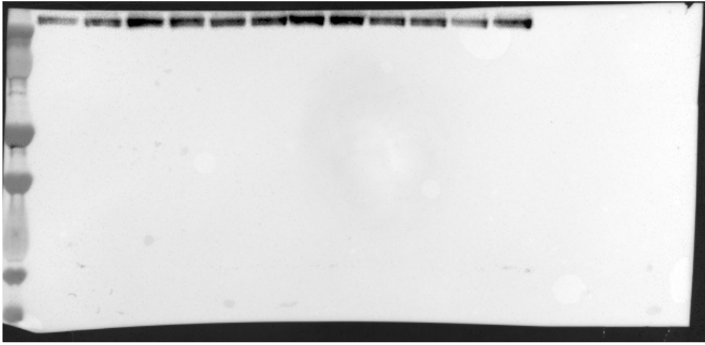

Supplement: Figure 6—source data 1. [file elife-94420-fig6-data1.zip › Figure_6_source_data_1/Fig6A_lysate_p110alpha_source_image_1.png]

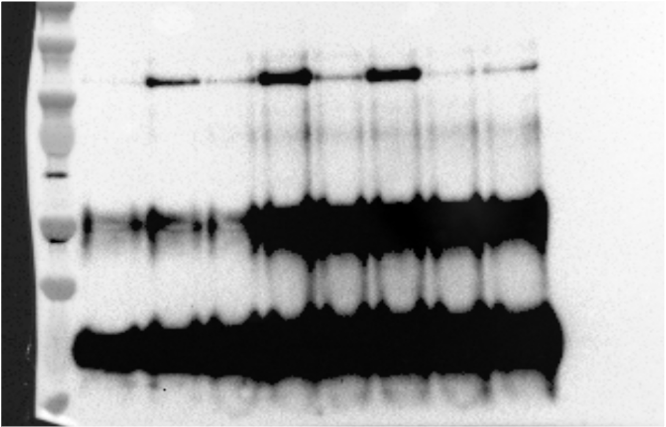

Supplement: Figure 6—source data 1. [file elife-94420-fig6-data1.zip › Figure_6_source_data_1/Fig6A_IP_p110alpha_replicate_1_source_image_2.png]

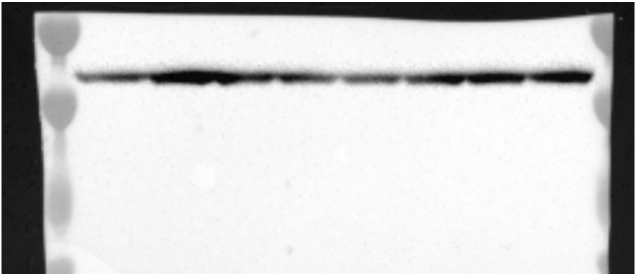

Supplement: Figure 6—source data 1. [file elife-94420-fig6-data1.zip › Figure_6_source_data_1/Fig6A_lysate_beta_actin_replicate_2_source_image_1.png]

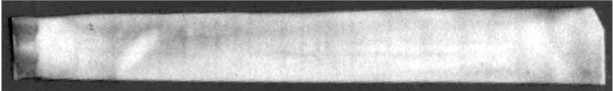

Supplement: Figure 6—source data 1. [file elife-94420-fig6-data1.zip › Figure_6_source_data_1/Fig6A_supernatant_IRS2_source_image_1.png]

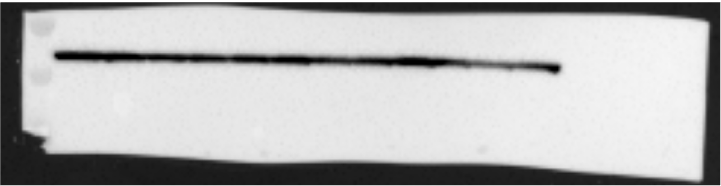

Supplement: Figure 6—source data 1. [file elife-94420-fig6-data1.zip › Figure_6_source_data_1/Fig6A_lysate_beta_actin_replicate_1_source_image_1.png]

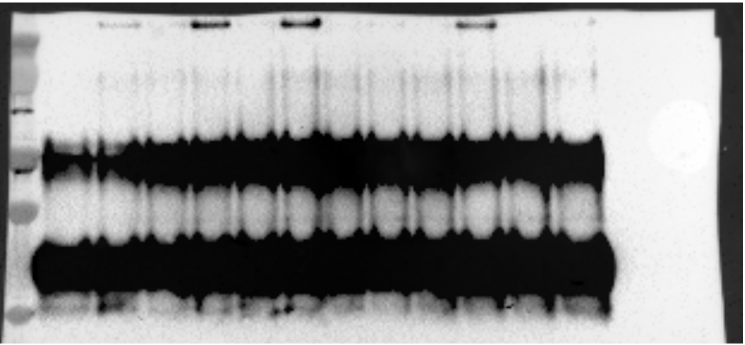

Supplement: Figure 6—source data 1. [file elife-94420-fig6-data1.zip › Figure_6_source_data_1/Fig6A_IP_p110alpha_source_image_1.png]

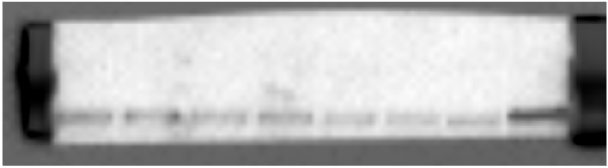

Supplement: Figure 6—source data 1. [file elife-94420-fig6-data1.zip › Figure_6_source_data_1/Fig6A_supernatant_IRS2_source_image_2.png]

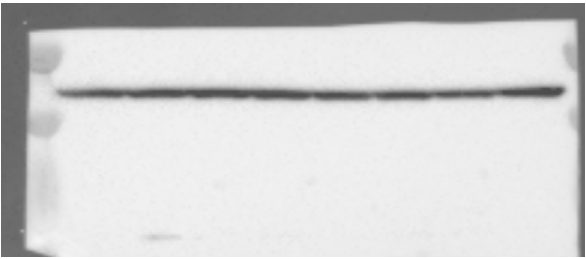

Supplement: Figure 6—source data 1. [file elife-94420-fig6-data1.zip › Figure_6_source_data_1/Fig6A_lysate_beta_actin_replicate_1_source_image_2.png]

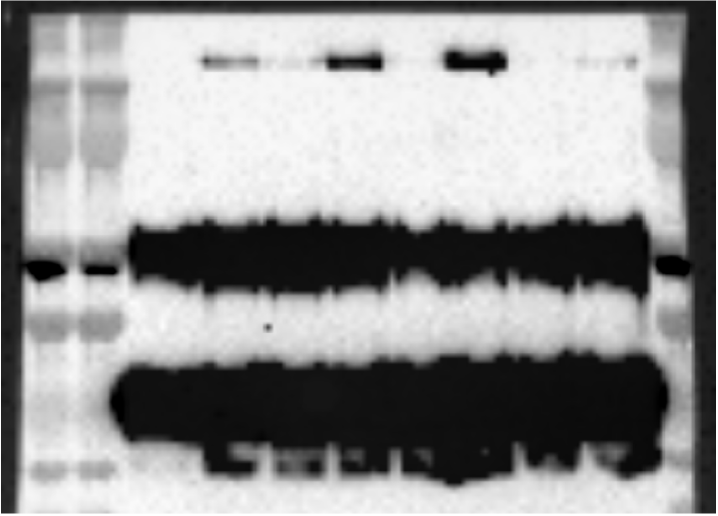

Supplement: Figure 6—source data 1. [file elife-94420-fig6-data1.zip › Figure_6_source_data_1/Fig6A_IP_p110alpha_source_image_2.png]

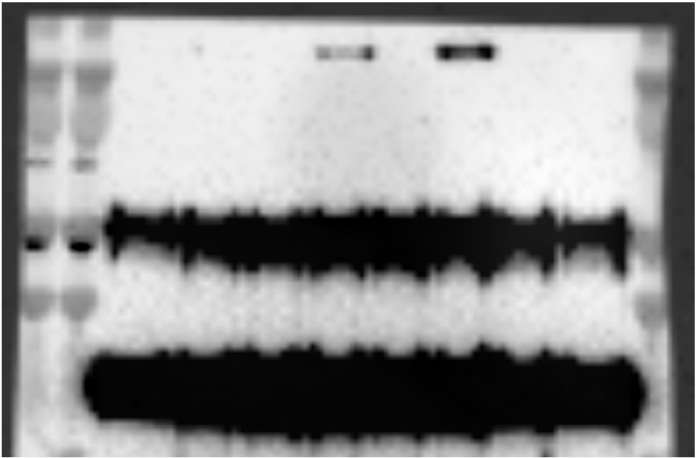

Supplement: Figure 6—source data 1. [file elife-94420-fig6-data1.zip › Figure_6_source_data_1/Fig6A_IP_p110alpha_replicate_2_source_image_1.png]
