# Supplementary material for: Paradoxical dominant negative activity of an immunodeficiency-associated activating PIK3R1 variant
Source: eLife. 2025 Jan 21;13:RP94420. doi: 10.7554/eLife.94420 (PMC11750134; doi:10.7554/eLife.94420)

Figure 5A – Images Shown

IP: IRS1

Total p85α

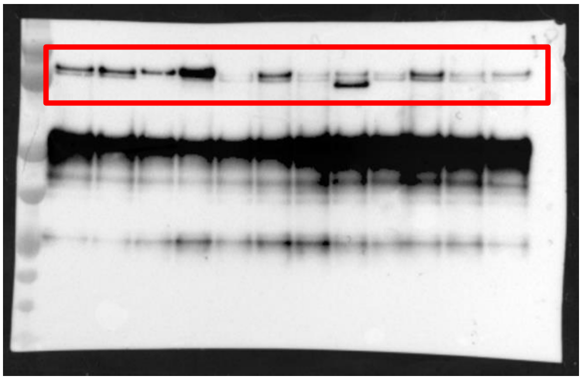

p110α

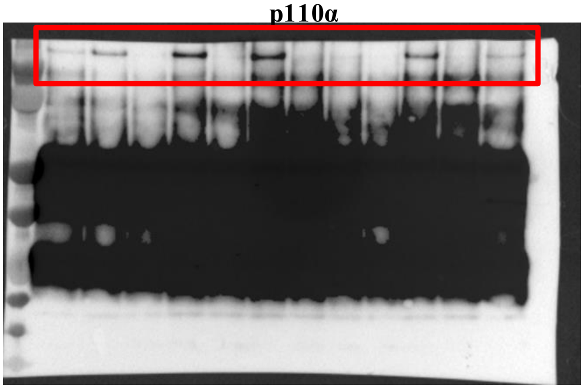

IRS1

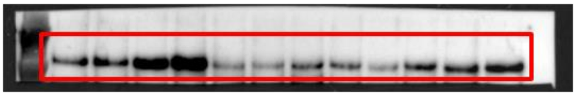

Supernatant

Total p85α

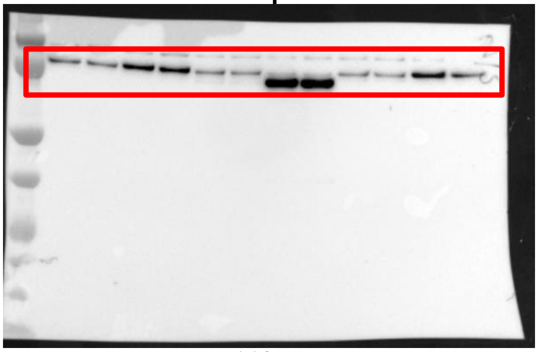

p110α

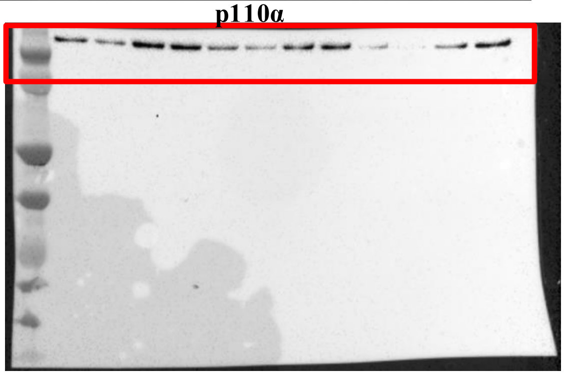

IRS1

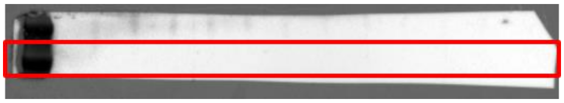

Total cell lysates

Total p85α

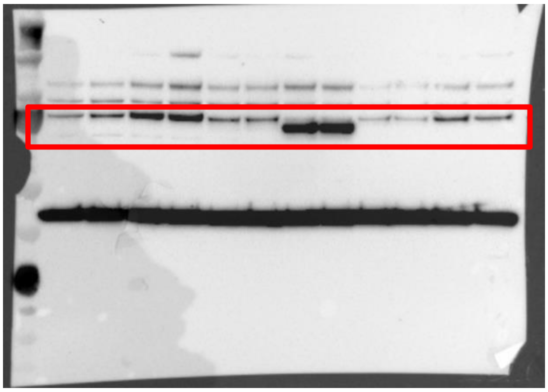

IRS1

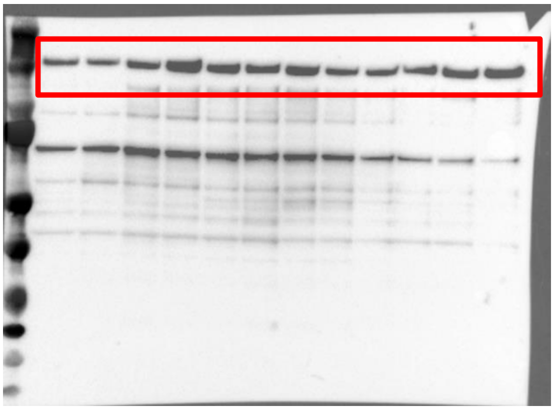

p110α

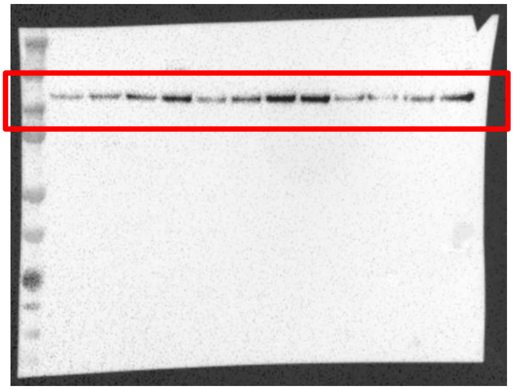

β-actin

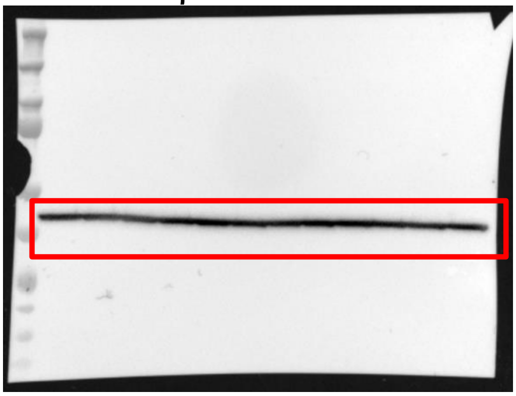

Figure 5A – Images Shown ctd

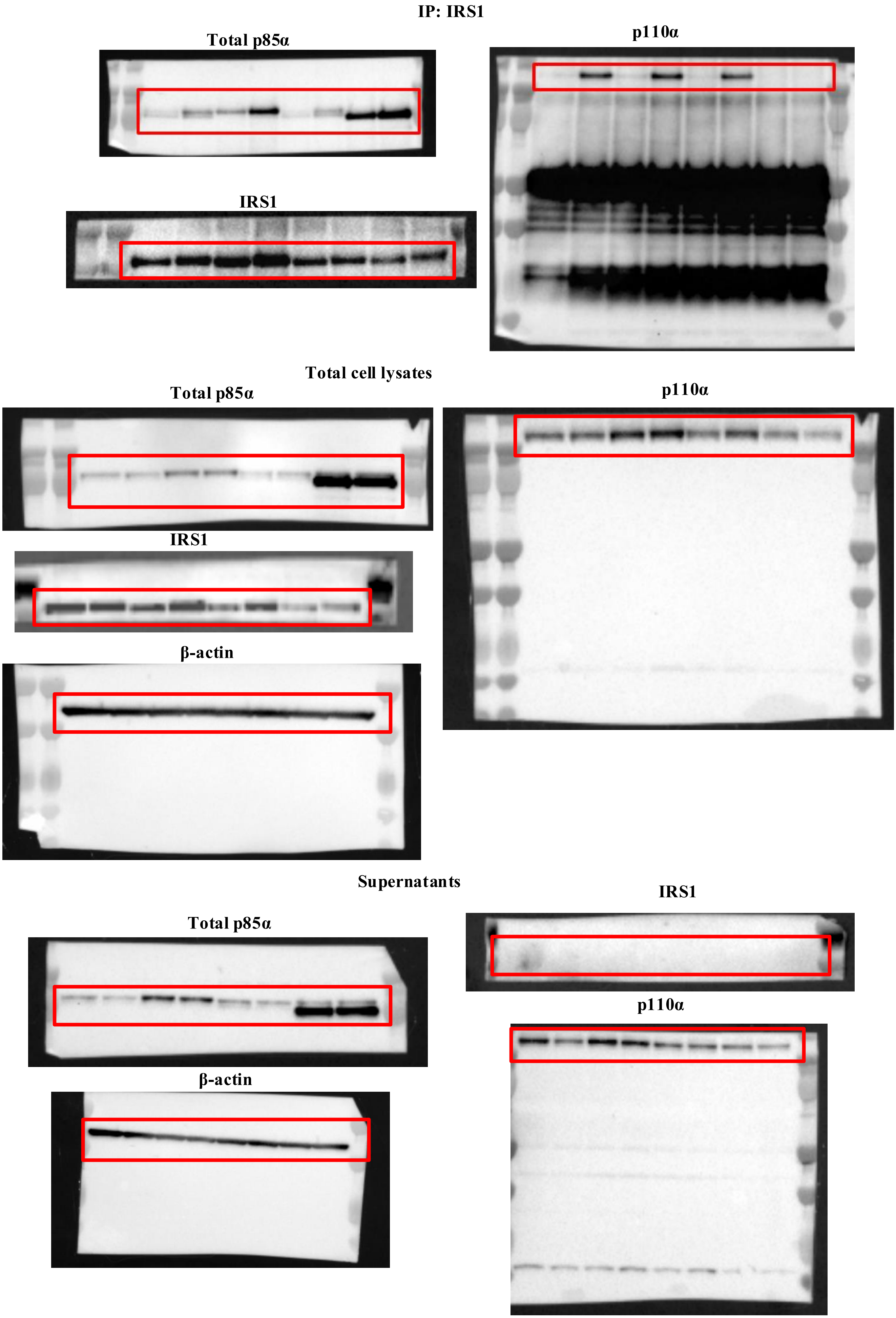

Figure 5A – Replicate 1

IP: IRS1

Total p85α

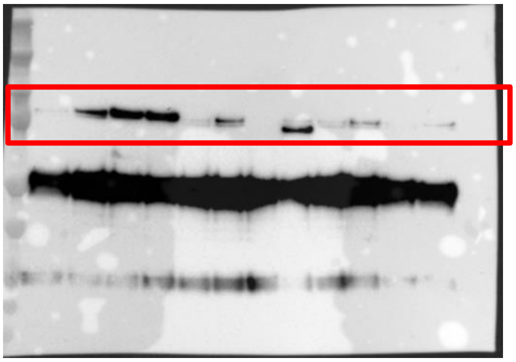

p110α

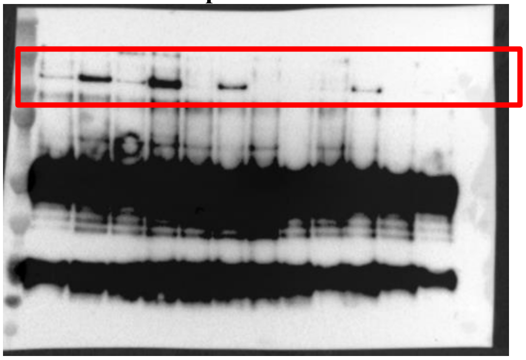

IRS1

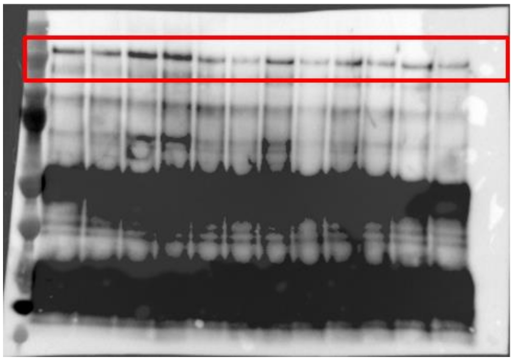

Total cell lysates

Total p85α

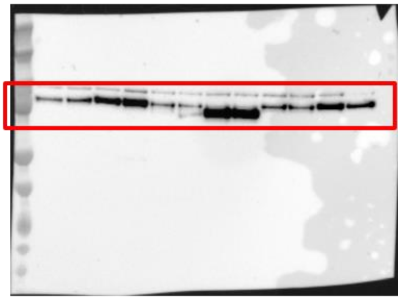

p110α

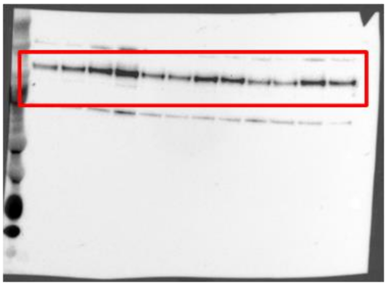

IRS1

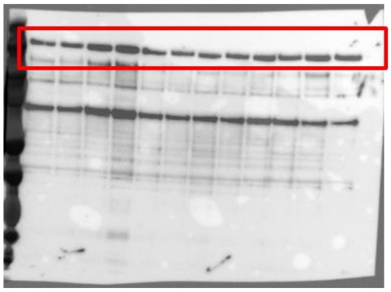

β-actin

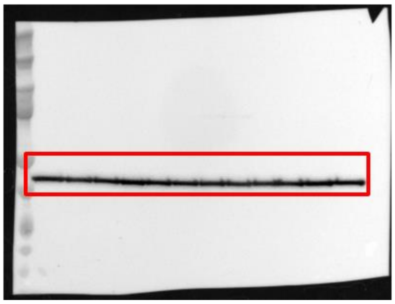

Supernatant

Total p85α

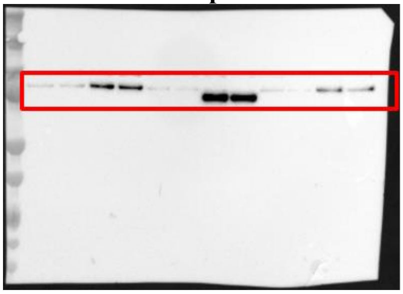

p110α

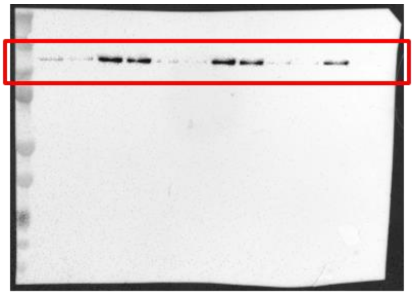

IRS1

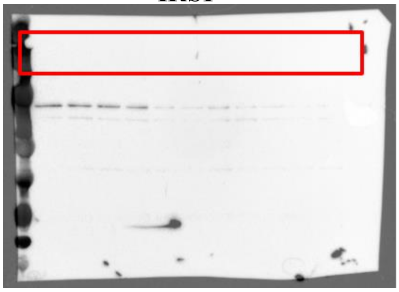

Figure 5A Replicate 1 ctd

(Note that p85a WT + DOX -/+ insulin samples are transposed compared to other replicates)

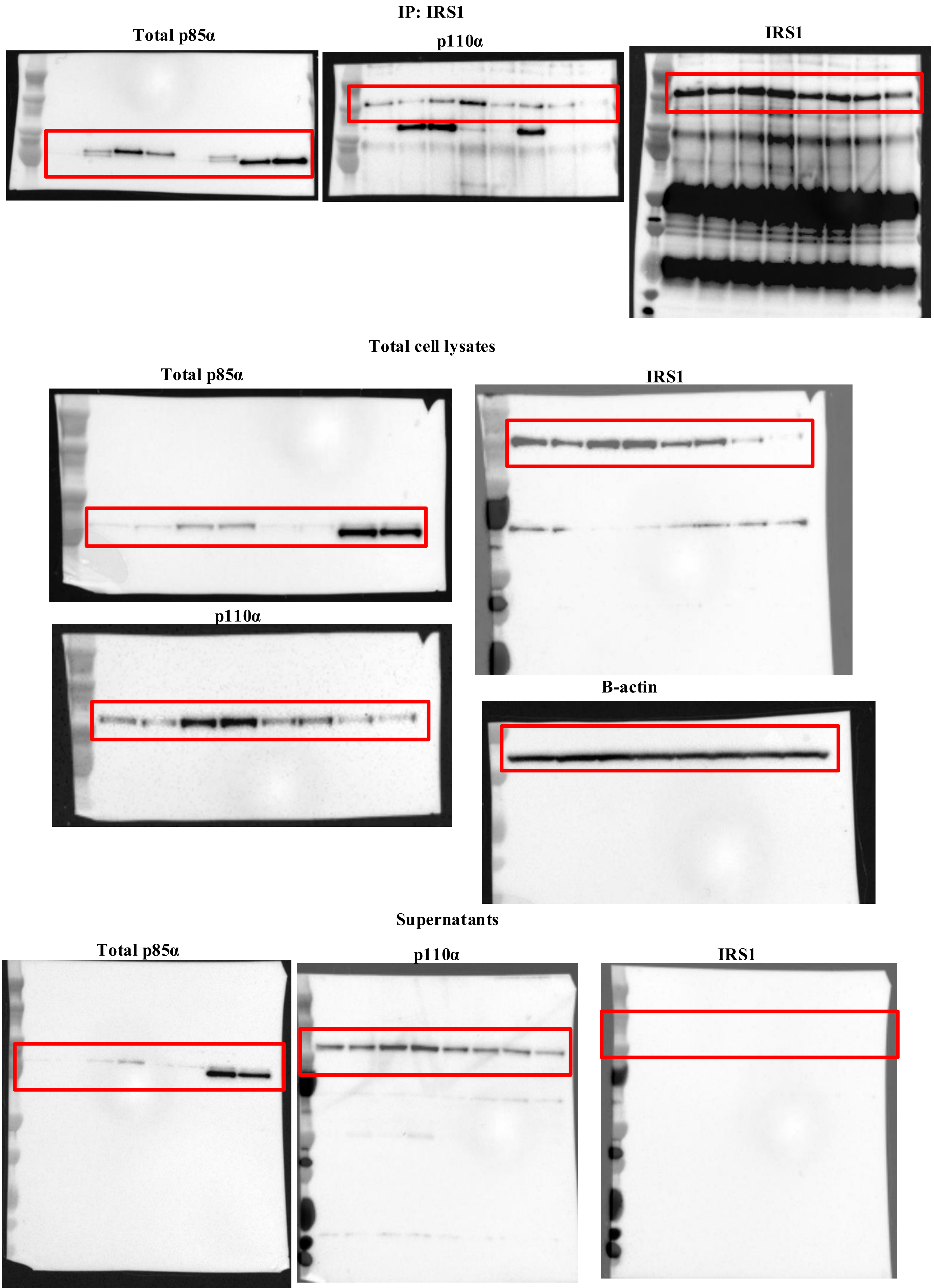

Figure 5A – Replicate 2

IP: IRS1

Total p85α

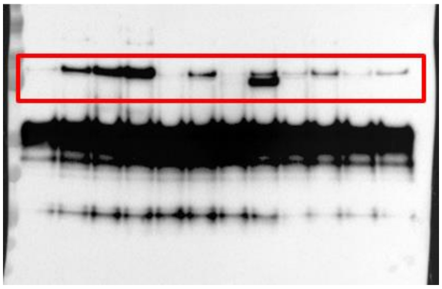

p110α

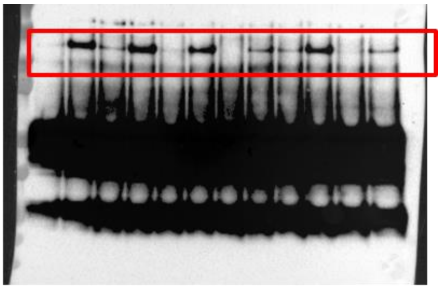

IRS1

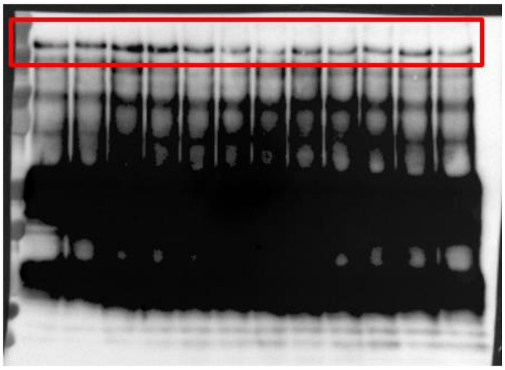

Total cell lysates

Total p85α

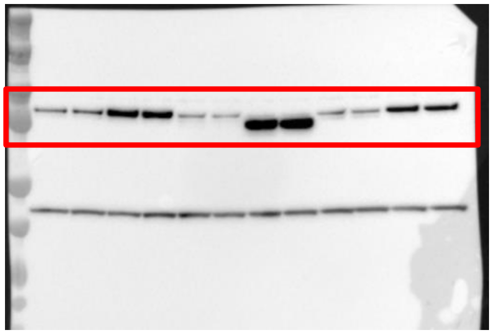

p110α

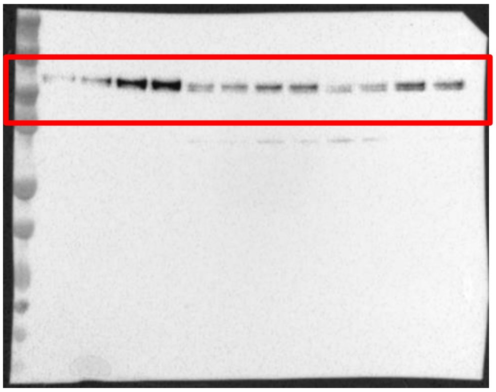

IRS1

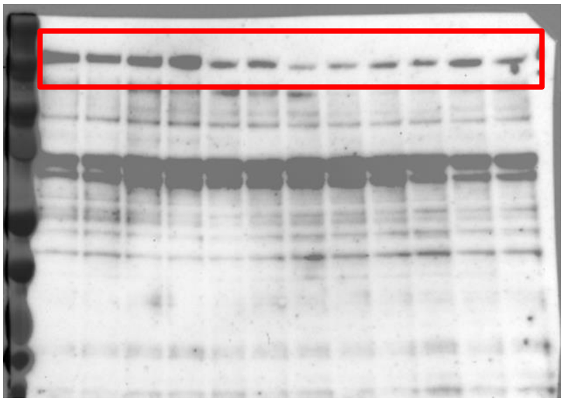

β-actin

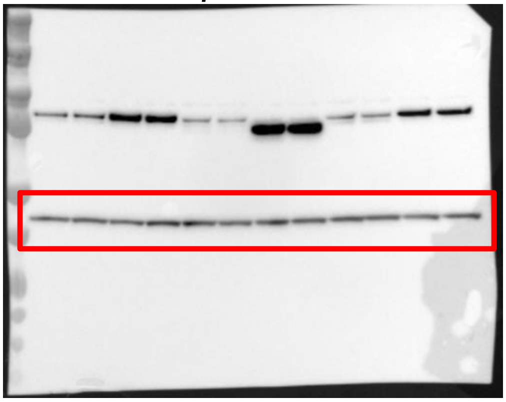

Supernatant

Total p85α

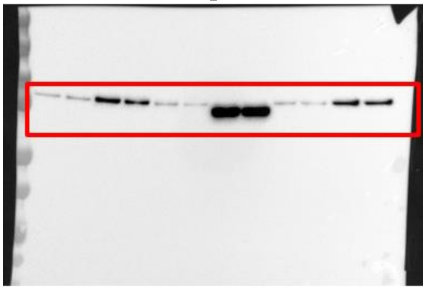

p110α

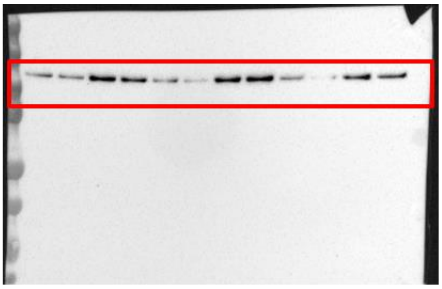

IRS1

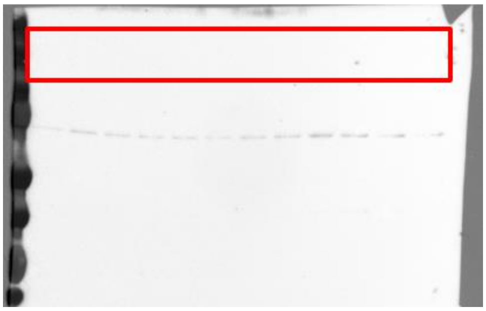

Figure 5A – Replicate 2 ctd

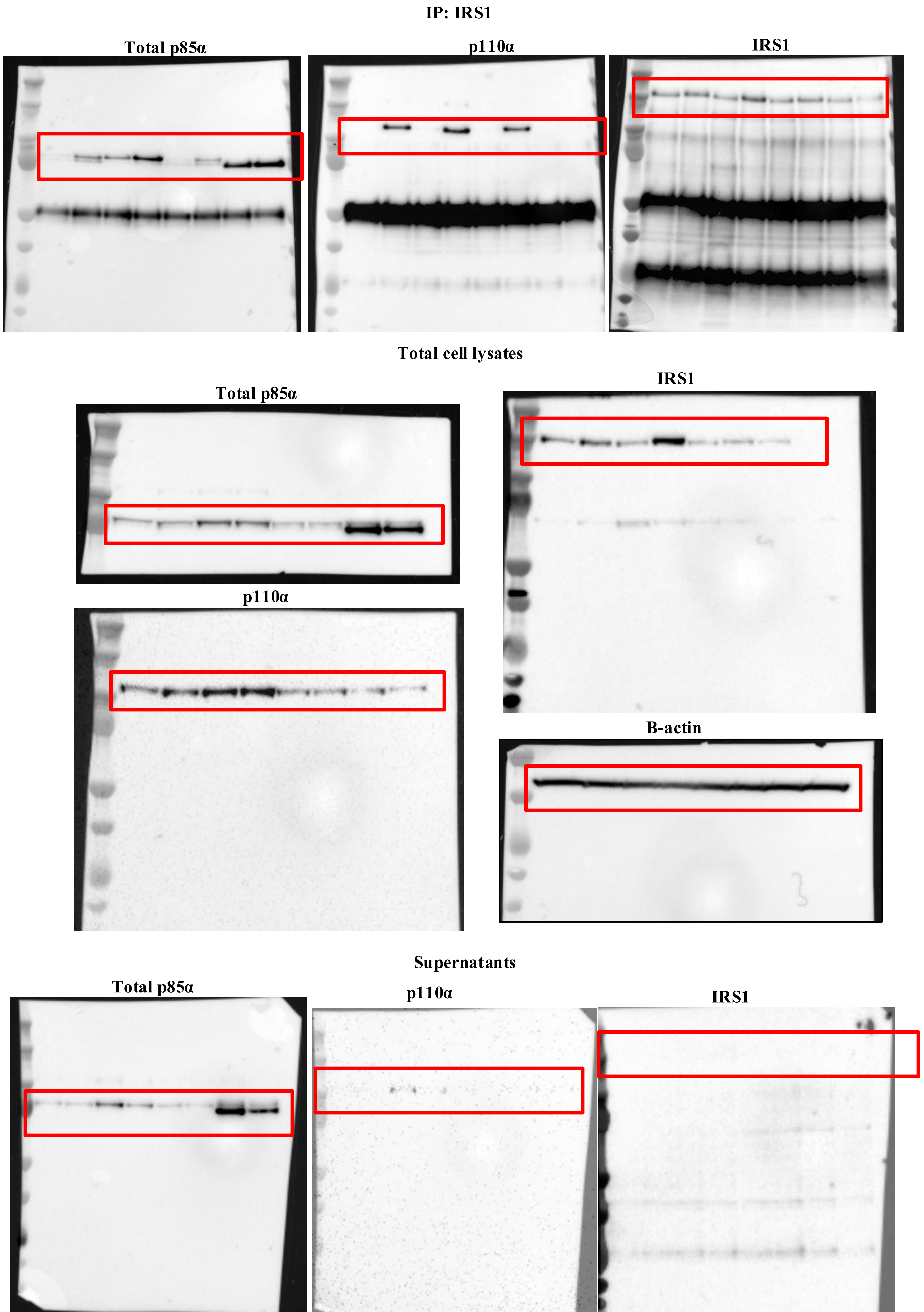

Supplement: Figure 5—source data 2. [file elife-94420-fig5-data2.pdf]
