# Supplementary material for: Paradoxical dominant negative activity of an immunodeficiency-associated activating PIK3R1 variant
Source: eLife. 2025 Jan 21;13:RP94420. doi: 10.7554/eLife.94420 (PMC11750134; doi:10.7554/eLife.94420)

Figure 6A – Images Shown

IP: IRS2

Total p85α

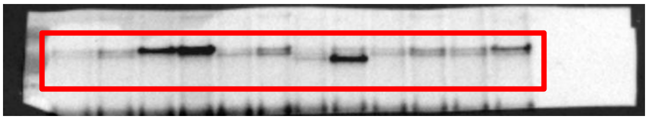

IRS2

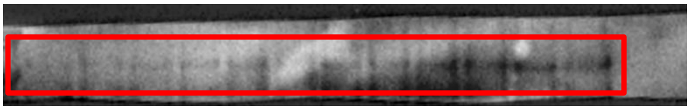

p110α

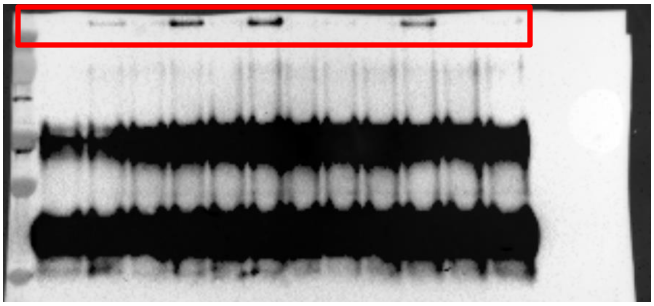

Total cell lysates

Total p85α

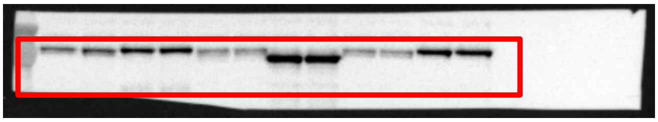

β-actin

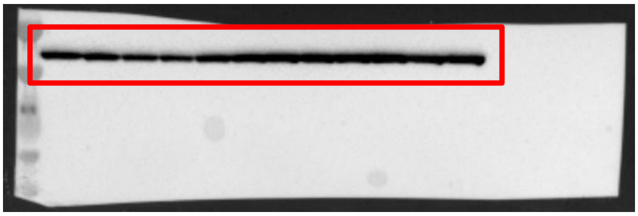

p110α

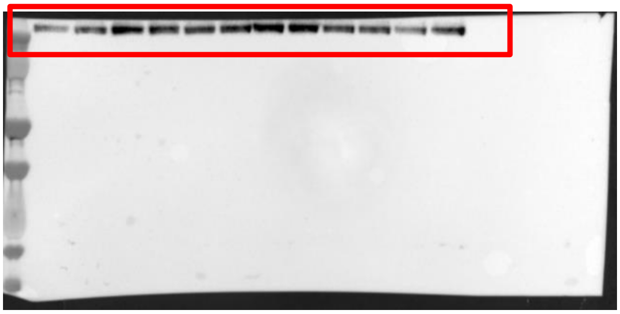

IRS2

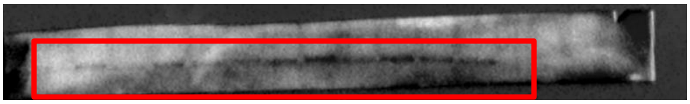

Supernatant

Total p85α

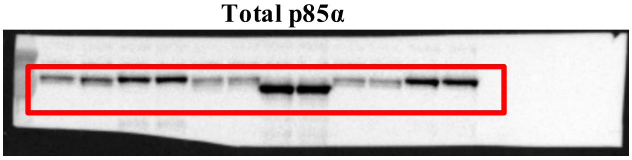

β-actin

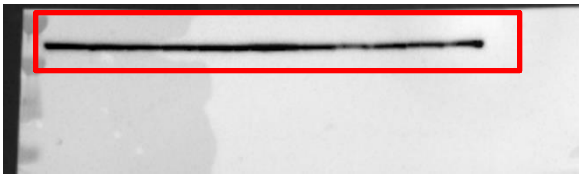

p110α

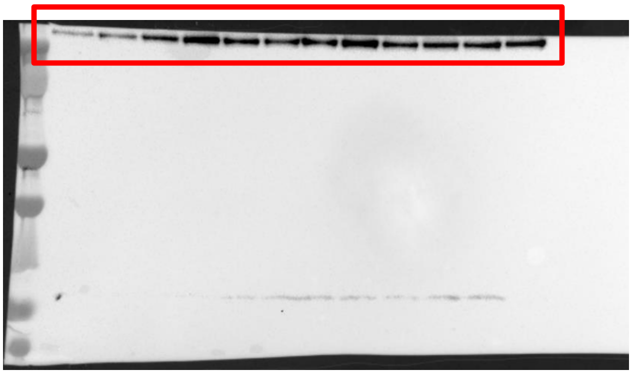

IRS2

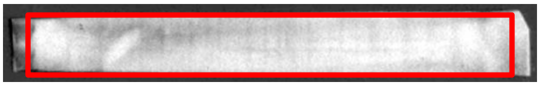

Figure 6A – Images Shown ctd

IP: IRS2

Total p85α

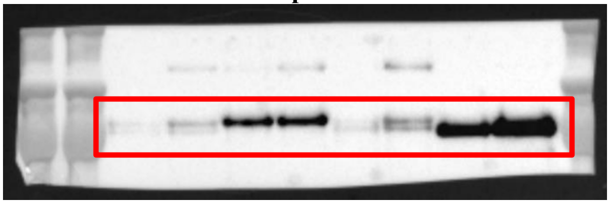

p110α

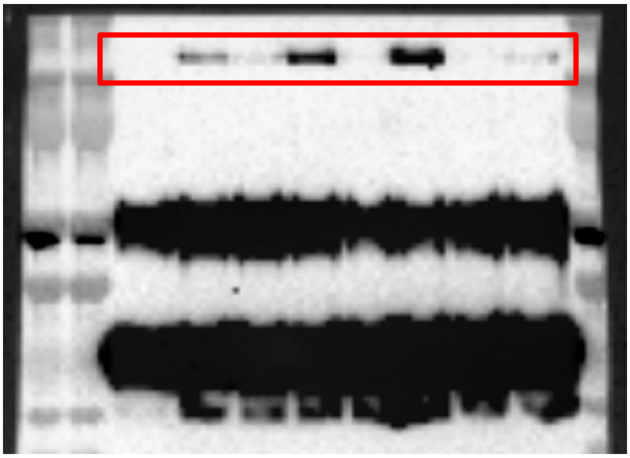

IRS2

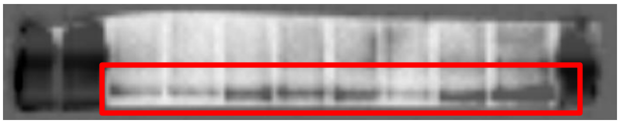

Total cell lysates

Supernatant

Total p85α

Total p85a

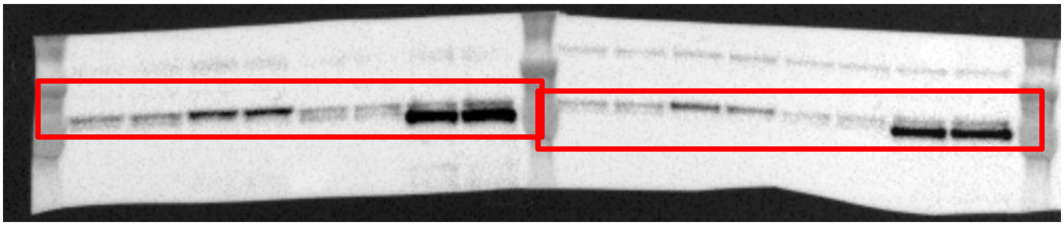

IRS2

IRS2

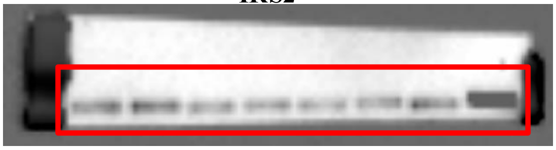

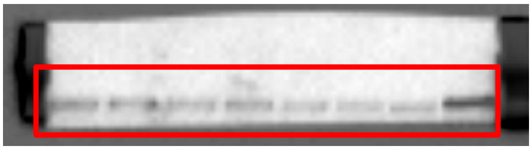

p110α

Total p110a

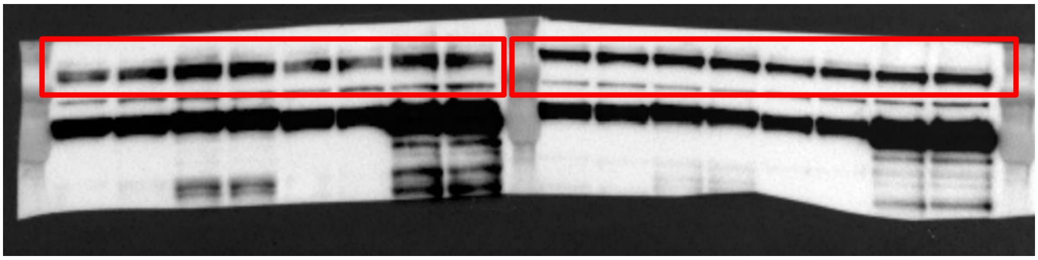

B-actin

B-actin

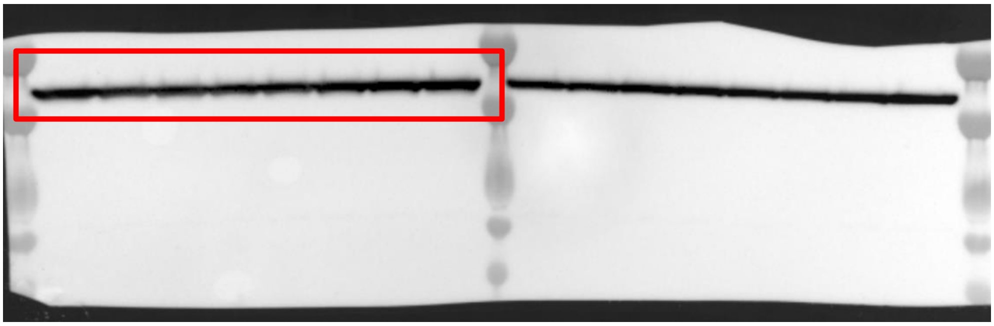

Figure 6A – Replicate 1

IP: IRS2

Total p85α

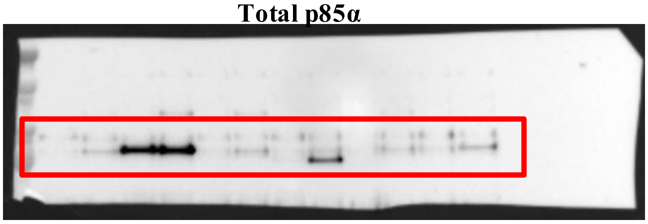

IRS2

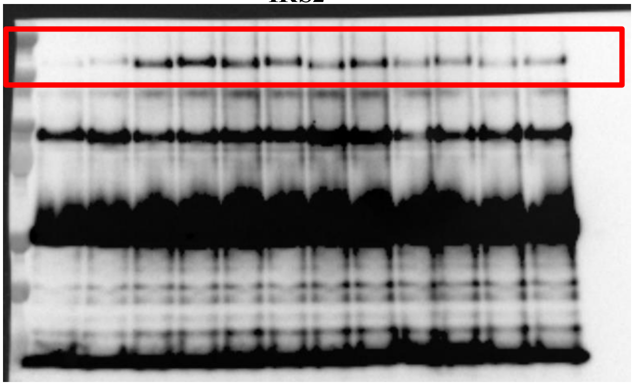

p110α

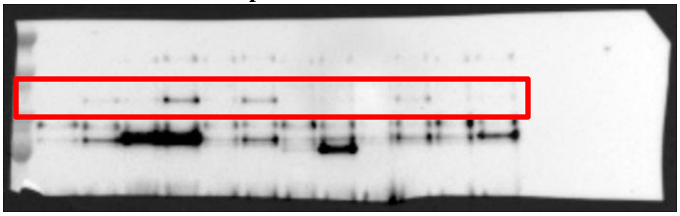

Total cell lysates

Total p85α

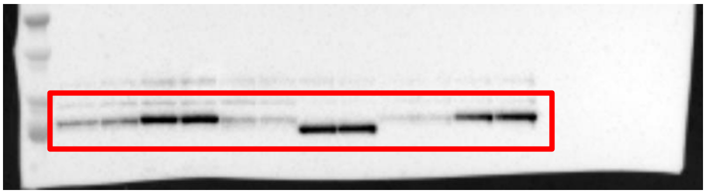

β-actin

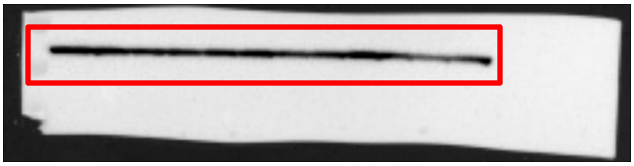

p110α

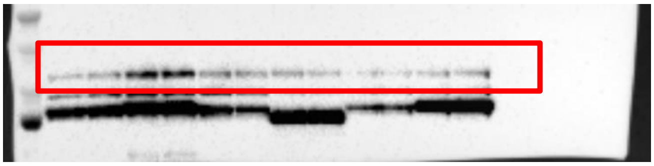

IRS2

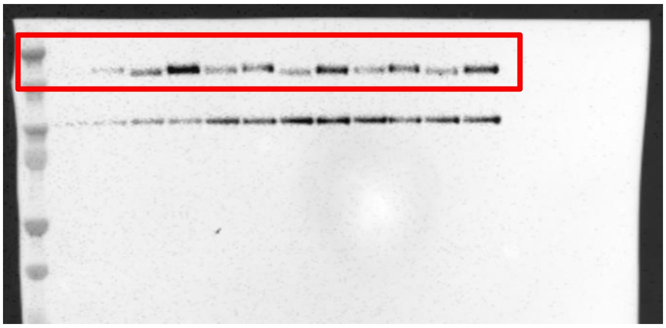

Supernatant

Total p85α

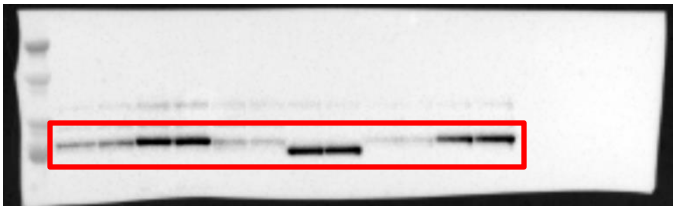

β-actin

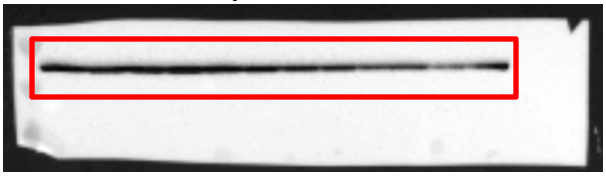

p110α

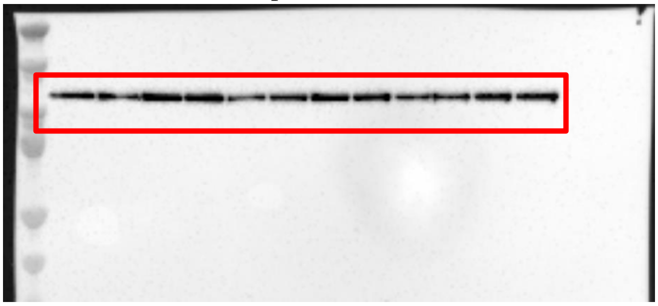

IRS2

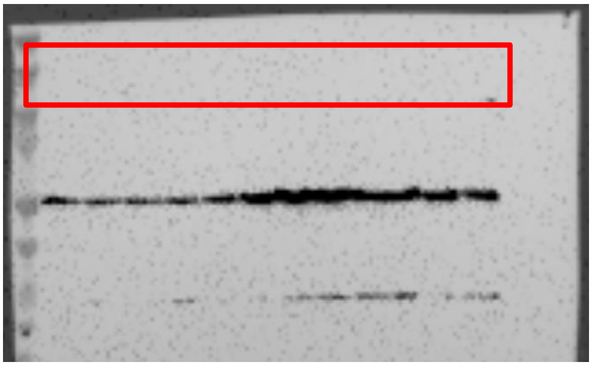

Figure 6A – Replicate 1 ctd

IP: IRS2

Total p85α

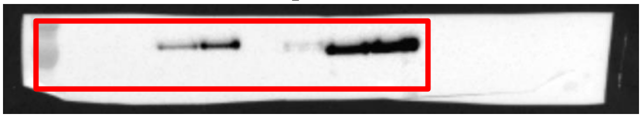

IRS2

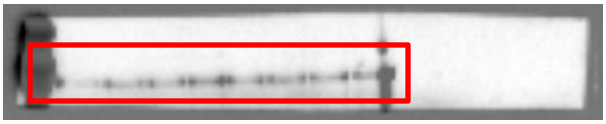

p110α

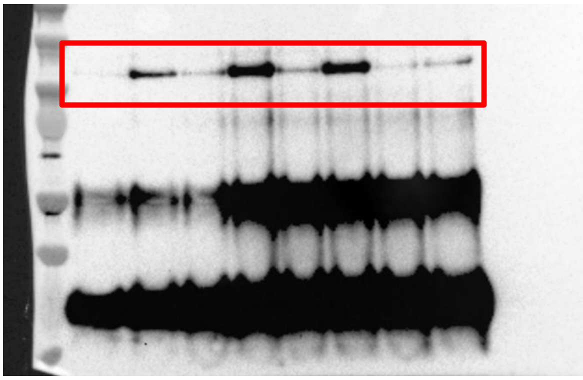

Total cell lysates

Total p85α

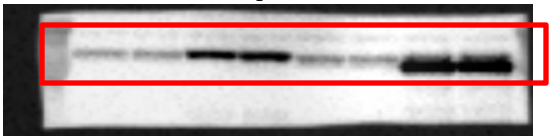

IRS2

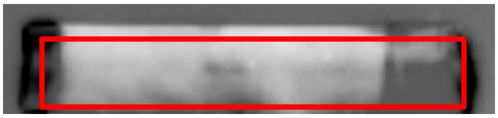

B-actin

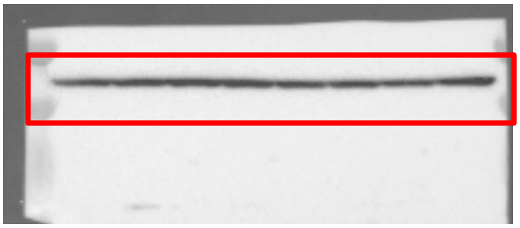

p110α

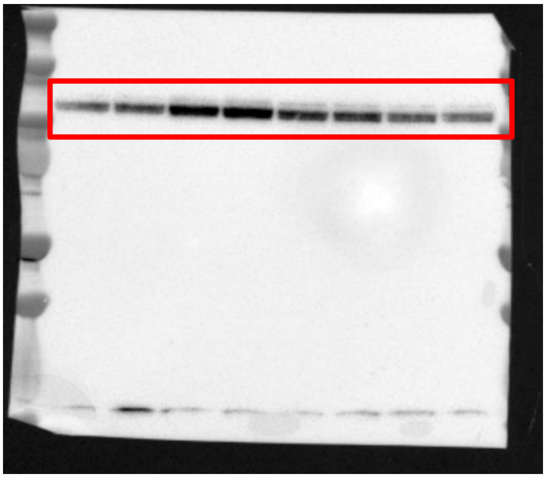

Supernatants

Total p85α

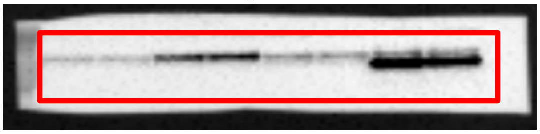

IRS2

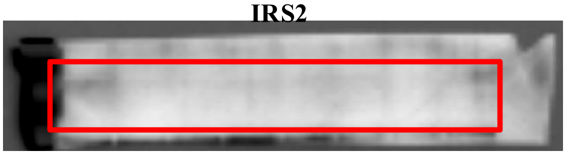

β-actin

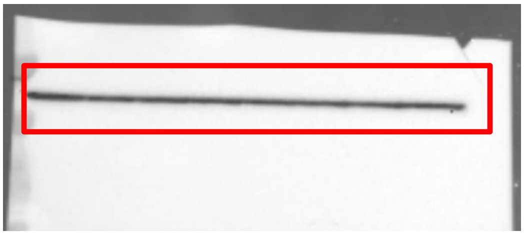

p110α

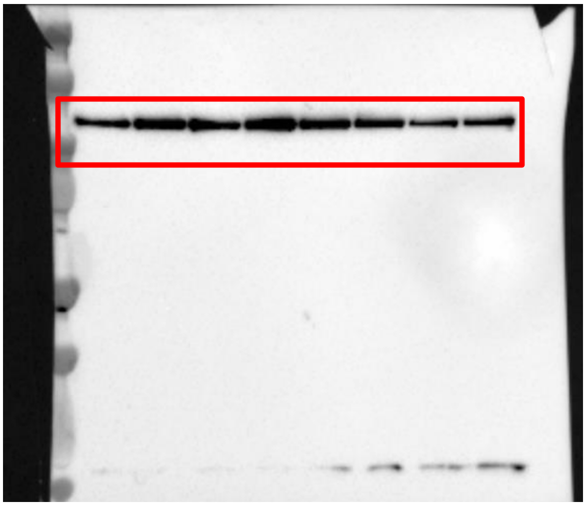

Figure 6A – Replicate 2

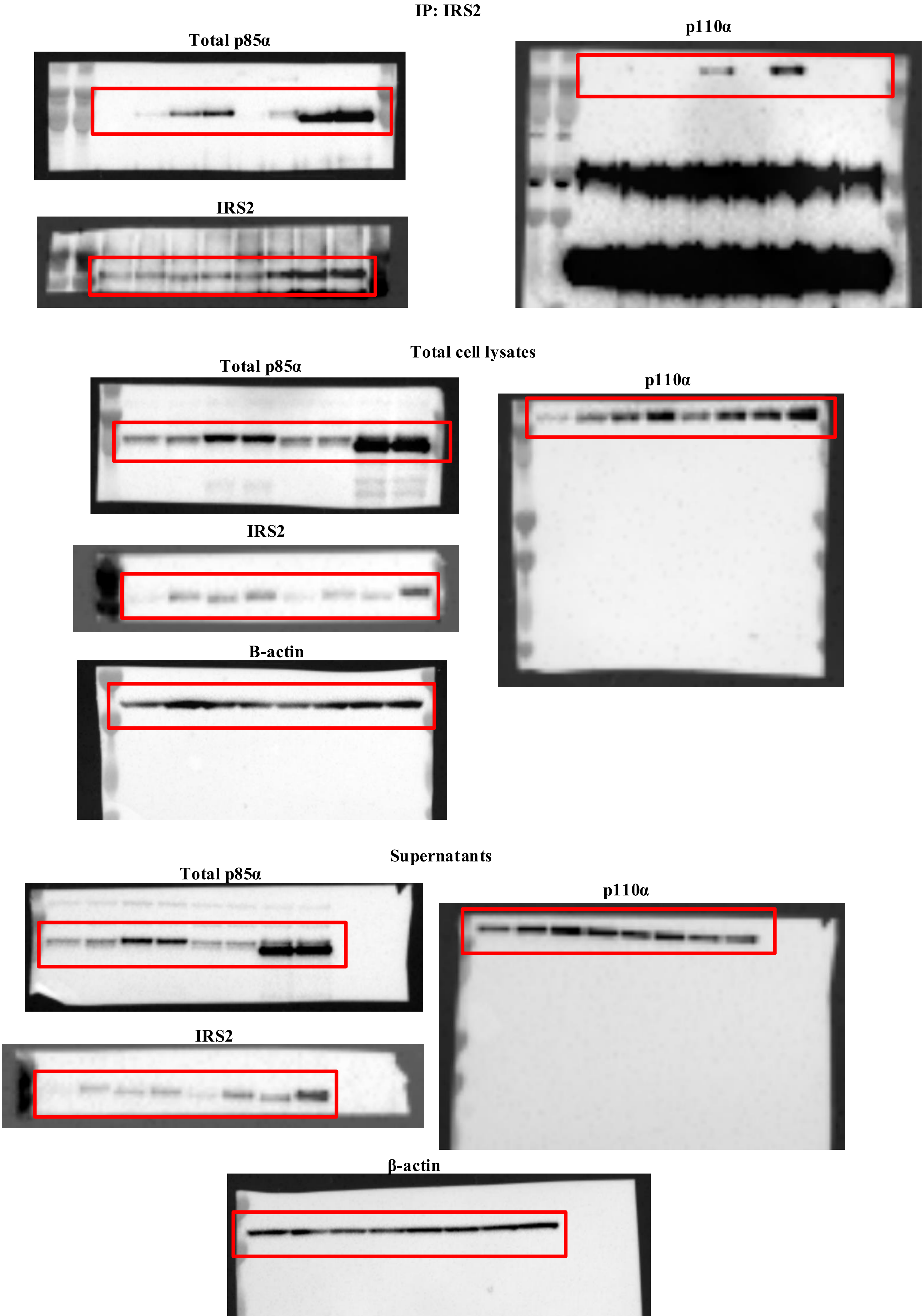

Supplement: Figure 6—source data 2. [file elife-94420-fig6-data2.pdf]
